# Advanced immunophenotyping of lymphocyte and monocyte subsets in healthy Australian adults using a novel spectral flow cytometry panel

**DOI:** 10.3389/fimmu.2025.1577206

**Published:** 2025-07-22

**Authors:** Ainsley R. Davies, Kristy Kwong, Zhijia Yu, Koula E. M. Diamand, Fei-Ju Li, Laurensia Kannitha, Sidra A. Ali, Abolfazl Amjadipour, Ann-Maree Padarin, Michael Devoy, Harpreet Vohra, Bahar Miraghazadeh, Simon H. Jiang, Anne Brüstle, Nicolas Cherbuin, Christopher J. Nolan, Matthew C. Cook, Elizabeth E. Gardiner, Stuart Read, Euan McNaughton, Katrina L. Randall

**Affiliations:** ^1^ Canberra Clinical Phenomics Service, John Curtin School of Medical Research, The Australian National University, Canberra, ACT, Australia; ^2^ Phenomics Translation Initiative, John Curtin School of Medical Research, Australian Phenomics Facility, The Australian National University, Canberra, ACT, Australia; ^3^ Department of Medicine, University of Cambridge, Cambridge, United Kingdom; ^4^ ANU Centre for Therapeutic Discovery, John Curtin School of Medical Research, Australian Phenomics Facility, The Australian National University, Canberra, ACT, Australia; ^5^ Cytometry, Histology and Advanced Spatial Multiomics, John Curtin School of Medical Research, The Australian National University, Canberra, ACT, Australia; ^6^ Division of Genome Science and Cancer, John Curtin School of Medical Research, The Australian National University, Canberra, ACT, Australia; ^7^ Division of Immunology and Infectious Disease, John Curtin School of Medical Research, The Australian National University, Canberra, ACT, Australia; ^8^ Department of Renal Medicine, The Canberra Hospital, Canberra, ACT, Australia; ^9^ National Centre for Epidemiology and Population Health, Australian National University, Canberra, ACT, Australia; ^10^ School of Medicine and Psychology, The Australian National University, Canberra, ACT, Australia; ^11^ South Australian Health and Medical Research Institute, Adelaide, SA, Australia; ^12^ Department of Immunology, and Department of Immunopathology, ACT Pathology, Canberra Health Services, Canberra, ACT, Australia

**Keywords:** spectral flow cytometry, lymphocyte, PBMC, immunophenotyping, autoimmunity, T cell, B cell

## Abstract

**Introduction:**

Lymphocytes play pivotal roles in disease pathogenesis and can be used as potential biomarkers for various immunological conditions. Yet, current flow cytometry methods used in clinical settings are often only capable of measuring between four to eight distinct lymphocyte populations. The purpose of our study was to measure many lymphocyte and monocyte populations from a single sample, with the long-term aim of validating our assay for diagnostic use in the Australian regulatory environment.

**Methods:**

We designed and optimised a novel 30-colour lymphocyte immunophenotyping panel tailored for use on a 3-laser (V-B-R) spectral flow cytometer. This panel measures over 50 lymphocyte and monocyte populations.

**Results:**

In this report we present data derived from 148 healthy individuals.

**Discussion:**

This lays the groundwork for future clinical application of spectral flow cytometry tests and offers a more comprehensive approach to lymphocyte and monocyte analysis with future implications for disease diagnosis and monitoring.

## Introduction

Flow cytometry is a powerful tool for measuring cellular characteristics in complex mixtures, such as human blood, and is invaluable in deciphering immune phenotypes (1). Rheumatoid arthritis (RA), multiple sclerosis (MS), and systemic lupus erythematosus (SLE) exhibit distinctive alterations in T-helper (Th) subsets, underscoring the importance of detailed analyses for accurate diagnosis (2–5). Additionally, phenotypic characterisation of patients with inborn errors of immunity has been facilitated by comprehensive analysis of lymphocyte subsets (6–8). However, contemporary methodologies using flow cytometry face inefficiencies, particularly in diagnosing diseases characterised by alterations in lymphocyte subsets. The current application of flow cytometry in diagnostic pathology is confined primarily to blood malignancies (9), acquired immune deficiency disease (10), and counting stem cell numbers before haematopoietic stem cell transplantation (11). Expanding the capabilities of clinical flow cytometry panels is advantageous, as it not only facilitates comparative analyses of specific subpopulations, but also conserves precious cell samples by reducing the need for multiple panels. This extends both the research and clinical applicability.

Clinical diagnostic flow cytometry panels typically detect eight or fewer antigens. For example, lymphocyte panels most commonly detect the cell surface markers CD45, CD3, CD19, CD4, CD8, CD16 and CD56 (12). Flow cytometry entered into clinical laboratories in the mid-1980s during the HIV pandemic to monitor CD4 T cell counts, using small panels on early conventional cytometers (10). Over time these panels have been adapted for other purposes, but advancements in technology have far outpaced the capabilities of the clinical tests. To address this limitation we set out to create a diagnostic-quality lymphocyte panel that utilises spectral flow cytometry which could be validated as an in-house diagnostic assay in the Australian regulatory framework. In contrast to conventional cytometry, spectral cytometry uses many more detectors to capture the entire spectrum of light emitted from a sample. Because of the detailed information gathered from the entire light spectrum, fluorophores that have unique broad spectra but similar peak emission wavelengths can be used together. Since many more dyes can be used simultaneously, this technology allows for the detection of a greater number of antigens in a single sample. However, as the number of fluorophores in a panel increases, so does the potential for data variability arising from non-biological factors, such as day-to-day fluorophore variation, and sensitivity to environmental conditions like time and temperature. To ensure that our panel accurately captures biological variation while minimising non-biological variability, we focused on rigorous optimisation and careful analysis. This approach was essential for producing high-resolution data with minimal error, allowing for reliable comparisons across experimental batches.

Our panel was initially designed to encompass T cell, B cell and natural killer (NK) cell populations, and underwent iterative refinements, ultimately focusing on T cell and B cell populations. We undertook rounds of optimisation, carefully considering antibody selection, staining optimisation and performing stability analyses. The aim was to develop a panel to measure as many markers as possible on our machine, and to quantitate those lymphocyte subsets in healthy donors from our region of Canberra, Australia to establish healthy lymphocyte frequencies and aid validation of an immune phenotyping assay which could provide results for clinical use.

## Materials and methods

### Ethics

Healthy blood donors were recruited through multiple individual studies. These were approved by the Australian National University (ANU) and ACT Health (Government) Human Research Ethics Committees (HRECs). The studies included Our Health In Our Hands (ACT Health 2019.ETH.00081, ANU HREC 2020/047), National Platelet Referral Centre (ANU HREC 2022/372), Centre for Personalised Immunology (ACT Health HREC ETH.1.15.015 and ETH.1.16.011, ANU HREC 2016/071) and Canberra Clinical Phenomics (ACT Health HREC 2023.ETH.00027). Written informed consent was obtained from all participants. While the recruitment of each donor varied between studies, common requirements included being over 18 years of age and the absence of them reporting an infectious illness on the day of donation.

### Blood collection and PBMC processing

Blood was collected into Acid Citrate Dextrose (ACD)-coated tubes, mixed by inversion and kept at room temperature (RT) before processing. Blood was processed via Ficoll-Paque (Cytiva 17144002) separation using Leucosep tubes (Greiner Bio-one 227289). 30 mL of blood was added to the pre-filled Leucosep tube, and tubes were centrifuged for 15 minutes at 800*g* at 21°C with no brake. The buffy coat containing peripheral blood mononuclear cells (PBMCs) was isolated with a sterile transfer pipette and moved to a clean 50 mL tube (ThermoFisher 339652). Cells were washed by filling the tube with phosphate buffered saline (PBS) and centrifuging for 5 minutes at 400*g* and 21°C with brake applied. Cells were counted via trypan blue exclusion using a Luna II Automated Cell Counter (*In Vitro* Technologies LOGL40002).

Washing and centrifugation steps were repeated after counting, and cells were resuspended in freezing media (90% heat-inactivated fetal calf serum (HI-FCS) (Sigma-Aldrich F9423) and 10% dimethyl sulfoxide (Sigma-Aldrich 472301)). Cells were resuspended in freezing media at 5 x 10^6^ cells/mL, and then aliquoted to 1 mL cryovials (ThermoFisher 377224). Cryovials were placed in a Mr. Frosty controlled-rate freezing apparatus (Nalgene 5100-0001) and moved immediately to the -80°C freezer.

### PBMC thawing

Wash medium (RPMI (Thermo Fisher 11875093) with 10% v/v HI-FCS (Sigma-Aldrich F9423)) was pre-warmed to 37°C. Once cryovials were thawed but still cool, cells were moved into a 15 mL tube, where the wash medium was added dropwise to a total volume of 10 mL. Tubes were centrifuged for 5 minutes at 400*g* at 21°C with the brakes turned on. Supernatant was decanted and the cell pellet was resuspended in 1 mL of warm FACS wash (1X PBS with 2.5% v/v FCS and 0.1% sodium azide (Australian Chemical Reagents 3902)). and counted on the Luna II Automated Cell Counter (*In Vitro* Technologies LOGL40002). After counting, the cell suspension was recentrifuged and resuspended to 1 x 10^7^ cells/mL.

### Antibody titration

2.5 x 10^5^ cells were plated in the wells of a 96-well U-bottom plate (Corning CLS3797). In the wells of another plate, antibody was mixed with FACS wash to create a 1:12.5 dilution then serially diluted 2-fold until reaching 1:200 dilution. Cells were centrifuged for 5 minutes at 400*g* at 21°C, supernatant was flicked off. 25 µL of each antibody dilution was mixed with cells for 30 minutes on ice. A stain of the highest concentration was used to create single-colour stained controls, and all other titration samples were stained with viability dye. Ghost Dye v450 diluted to 1:4000 (determined by titration) in PBS was added to the cells and incubated for 20 minutes at RT. Cells were fixed with eBioscience Foxp3/Transcription Factor Staining Buffer Set (ThermoFisher 00-5523-00) following kit instructions or fixed with 1% paraformaldehyde in PBS for 20 minutes at RT.

### Antibody staining

When staining cells with the full 30-colour panel, 2 x 10^6^ cells were plated into wells of a 96-well U-bottom plate (Corning CLS3797). Wells were topped up to 120 μL with FACS wash, and centrifuged for 5 minutes at 400*g* at 21°C.

Antibody cocktails were prepared within 1 hour of use. Optimal staining concentrations were determined for a final staining volume of 50 µL split into two layers (description of sequential staining described in Results section). The first antibody layer contained three antibodies (CCR6 PE-Dazzle 594, TCRγδ Alexa Fluor 660, and CXCR5 BV421) diluted in Brilliant Stain Buffer (BD 566349), to a volume of 15 μL per well. The first 3-colour layer was added to the cell pellet, mixed, the plate was covered and kept at RT for 30 minutes in the dark. This was a 3.3X cocktail, which was diluted to 1X when the second layer is added on top.

The remaining 26 antibodies (excluding viability dye) and True-Stain Monocyte Blocker (Biolegend 426101) were diluted in Brilliant Stain Buffer to a volume of 35 µL per well. The 26-colour layer was added directly to the well, mixed and the plate was stored for another 30 minutes at RT. This final staining volume was 50 μL and a 1X concentration. Wells were topped up to 120 μL with FACS wash, and centrifuged for 5 minutes at 400*g* at 21°C. An aliquot of ViaDye Red (Cytek R0-00008) was thawed and diluted 1:5000 in 1X PBS. Samples were stained with 50 µL of diluted ViaDye Red and incubated at RT in the dark for 20 minutes.

At this point of the staining process, single-colour control wells were prepared using either 2.5 x 10^5^ PBMCs or CompBead Plus compensation beads (BD 560497), depending on which was more suitable for each antibody. To determine if beads were a suitable control particle, both the median fluorescence intensity (MFI) and normalised emission spectra were measured. Beads had to pass two criteria; MFI needed to be as bright or brighter than the fully-stained sample and the normalised emission spectra had to be identical to the normalised emission spectra of the equivalent cell control.

Controls were stained with 25 µL of antibody mixture and incubated at RT in the dark for 20 minutes. Samples and single-colour controls were topped up to 120 µL with FACS wash, and centrifuged for 5 minutes at 400*g* at 21°C.

1X fixation buffer was created by diluting 4% paraformaldehyde (ThermoFisher J19943.K2) 1:4 with 1X PBS. All wells were fixed with a volume of 100 µL per well and incubated at RT in the dark for 20 minutes. Wells were topped up to 200 µL with FACS wash, and centrifuged for 5 minutes at 400*g* at 21°C. This wash step was repeated, and finally samples were resuspended in FACS wash in the plate to prepare for plate-loader acquisition.

### Control and sample acquisition

This panel was designed for acquisition on the Cytek Northern Lights (Cytek Biosciences, Inc., Fremont, California) operating in the Australian National University Cytometry, Histology and Advanced Spatial Multiomics (CHASM) facility at the John Curtin School of Medical Research. This machine is equipped with three solid-state lasers (violet, blue and red), 38 detection channels (V1-V16, B1-B14, R1-R8), and three scatter channels (FSC, SSC-A, SSC-B). The machine used SpectroFlo software (Version 3.3.0) for acquisition, spectral unmixing, and data export.

Before acquisition, the machine was allowed to warm up for 30 minutes. Instrument QC was run daily using SpectroFlo QC beads (CYTEK). Reference controls were then acquired, recording a minimum of 100,000 cells or 10,000 beads, or the entire volume of the well (whichever was reached first). Cytek Assay Settings (CAS) were used with only changes to FSC and SSC to ensure cells and beads were on scale. Unmixing was performed within SpectroFlo, and the similarity matrix was checked to ensure that the similarity index calculated was <28. If similarity was >28, controls were scrutinised to identify possible contamination or dye degradation.

Once unmixing had been performed, sample spectra were recorded. For all samples the contents of the well, or 1.5 x 10^6^ cells were recorded, whichever was reached first. Both raw and unmixed FCS files were exported at the end of each experiment.

### Manual analysis

FlowJo™ v10.8 Software (BD Life Sciences) was used for FCS file analysis. Raw FCS files of single-colour controls were imported into Flow-Jo. Gating on the positive-stained population of cells or beads, the MFI in every detection channel was calculated. This was normalised in two stages; first to background (unstained), and second to the peak channel, resulting in a normalised emission spectra. Normalised emission spectra on each batch were compared to previous batches to identify any variation in emission of individual fluorophores.

In a new workspace file, the unmixed sample files were imported into FlowJo. A series of gates were applied to clean the data, including a time gate (to exclude pressure-related variations), two gates to exclude doublets, a gate to exclude any remaining red blood cells, two gates to exclude antibody aggregates, and a gate to exclude debris. The single cell events remaining in this gate were exported as a new FCS file and were analysed in a new FlowJo workspace. This minimised file size allowing faster computation. For manual analyses, a series of two-dimensional gates were applied to the data to segregate T and B cell populations. Population frequencies were recorded as a frequency of a relevant parent or grandparent population. Frequencies were exported as csv files, and graphical visualisation was performed using the ggplot2 package (13) in R Studio.

### High dimensional analysis

After gating on Live CD45^+^ cells, data from each fluorescent parameter were carefully scaled using min/max, width-basis and positive decade settings to ensure that negative populations were unimodal and normally distributed (14). Uniform Manifold Approximation and Projection (UMAP) (15) was performed using the UMAP_R plugin for FlowJo or the Spectre package (16) in R Studio. We set both tools to calculate nearest neighbours = 15 with distance measurement = Euclidean and minimum distance = 0.5. All fluorescent parameters were included in dimensionality reduction except for CD45 BV570 and Viability ViaDye Red.

## Results

### Panel design, marker and fluorophore selection

In the initial phase of panel design, we selected the lymphocyte populations we aimed to detect, guided by the clinical relevance of these cells in disease states. This included T cell subsets (αβ T cells, γδ T cells, CD4/CD8, naïve, central memory, effector memory, terminal effector memory expressing RA (TEMRA) T cells, T-follicular helper (Tfh) cells, Th subsets (Th1, Th2, Th17, Th1/17), exhausted T cells, regulatory T cells (Tregs)), B cell subsets (transitional, naïve, memory, unswitched memory, plasmablasts and plasma cells, atypical B cells, κ-light chain vs. λ-light chain usage) and NK cells. We then selected 31 cell surface markers that would allow detection of these populations.

The selection of antibodies followed a systematic procedure. Initially, bright fluorophores were assigned to antigens expressed at lower levels, while dim fluorophores were allocated to highly expressed antigens. Subsequently, in instances where the emission spectra of fluorophores exhibited high similarity, antibodies were designated for mutually exclusive antigens. The CYTEK Similarity Index is a metric that can be used to determine how alike a pair of emission spectra are. This index ranges from 0 (distinct) to 1 (identical). As previously described, similarity indices of 0.98 or less were deemed suitable for use (17, 18). For instance, CXCR5 and CD16 were respectively assigned to BV421 and Super Bright 436, with a similarity index of 0.97. Another illustration involves CD20 and CD56, which were allocated to redFluor 710 and cFluor R720, displaying a similarity index of 0.94.

The Complexity Index is another metric created by CYTEK to evaluate fluorophore combinations. This index considers the similarity of a combination of spectral signatures. A higher Complexity Index suggests that there are multiple highly similar fluorophores within a panel, and so the index can be used to predict the success of a certain combination (18). We used the CYTEK Cloud tool during our initial panel design in November 2022 and received a Complexity Index of 61.54. Subsequently, the CYTEK online tool underwent updates to incorporate new emission spectra data. In August 2023, the Complexity Index of the same panel was calculated at 247.69, and at the current time of writing (December 2024) the Complexity Index of the panel is 139.31. It became clear through the optimisation process (next section) that this combination of 31 colours would be unsuitable for practical use. Through the experiments described below, the final panel was determined, striking a balance between detecting as many populations as possible while minimising spread to maintain excellent population resolution. The final fluorophore selections are shown in **Table 1** (see the initial panel design in **Supplementary Table 1**).

**Table 1:** Final 30 colour panel design.

Laser	Peak channel	Marker	Fluorophore	Supplier	Cat	Clone	Ab host species	Ab Isotype	Research Resource Identifiers (RRIDs)	Dilution factor	μg per test
405nm	V1	CXCR5	BV421	Invitrogen	404-9185-41	MU5UBEE	Mouse	IgG2b κ	AB_2925549	25	0.1
	V2	CD16	Super Bright 436	Invitrogen	62-0168-41	eBioCB16	Mouse	IgG1 κ	AB_2688189	25	0.024
	V3	CD20	cFluor v450	Cytek	R7-20015	2H7	Mouse	IgG2b κ	AB_3669052	100	N/A
	V5	CD11c	BV480	BD	566184	B-ly6	Mouse	IgG1 κ	AB_2739581	12.5	0.8
	V5	CD4	cFluor V505	Cytek	R7-20248	SK3	Mouse	IgG1 κ	AB_3669053	400	N/A
	V7	IgG	BV510	BD	563247	G18-145	Mouse	IgG1 κ	AB_2738093	50	0.05
	V8	CD45	BV570	BioLegend	304034	HI30	Mouse	IgG1 κ	AB_2563426	200	0.025
	V10	Igκ	BV605	BD	752959	G20-193	Mouse	IgG1 κ	AB_2917914	100	0.1
	V11	CCR7	BV650	BioLegend	353233	G043H7	Mouse	IgG2a κ	AB_2563867	12.5	0.4
	V13	CD24	BV711	BioLegend	311135	ML5	Mouse	IgG2a κ	AB_2566578	25	0.2
	V14	PD-1	BV750	BioLegend	329965	EH12.2H7	Mouse	IgG1 κ	AB_2810505	12.5	0.8
	V15	CD45RA	BV785	BioLegend	304139	HI100	Mouse	IgG2b κ	AB_2561369	300	0.0067
488nm	B1	IgM	BB515	BD	564622	G20-127	Mouse	IgG1 κ	AB_2738869	100	0.1
	B2	CD57	cFluor B532	Cytek	RC-00127	HNK-1	Mouse	IgM	N/A	25	N/A
	B3	CD14	cFluor B548	Cytek	R7-20116	63D3	Mouse	IgG1 κ	AB_3669054	50	N/A
	B4	CD21	PE	BD	561768	B-ly4	Mouse	IgG1 κ	AB_10897839	50	0.025
	B6	CCR6	PE-Dazzle 594	BioLegend	353429	G034E3	Mouse	IgG2b κ	AB_2564232	100	0.025
	B7	CD19	PE-Fire 640	BioLegend	302273	HIB19	Mouse	IgG1 κ	AB_2860772	100	0.025
	B8	CD8	PerCP	Tonbo	67-0087-T025	SK1	Mouse	IgG1 κ	AB_2621917	50	0.025
	B9	Igλ	cFluor B690	Cytek	R7-20260	1-155-2	Mouse	IgG1 κ	AB_3669056	25	N/A
	B10	CD25	cFluor BYG710	Cytek	RC-00103	BC96	Mouse	IgG1	N/A	12.5	0.024
	B12	IgD	cFluor BYG750	Cytek	RC-00521	IgD26	Mouse	IgG1	N/A	200	N/A
	B13	CXCR3	PE-Cy7	BioLegend	353719	G025H7	Mouse	IgG1 κ	AB_11219383	100	0.1
	B14	CD38	PE-Fire 810	BioLegend	397225	S17015F	Mouse	IgG2a κ	AB_2894562	200	0.05
640nm	R1	IgA	APC	Miltenyi	130-113-998	IS11-8E10	Mouse	IgG1 κ	AB_2733421	200	N/A
	R2	CD127	AF647	BioLegend	351317	A019D5	Mouse	IgG1 κ	AB_10896063	200	0.025
	R3	TCRgd	AF660	BioLegend	331239	B1	Mouse	IgG1 κ	AB_2892398	25	0.4
	R7	Viability	ViaDye Red	Cytek	R7-60008	N/A	N/A	N/A	N/A	5000	N/A
	R7	CD3	APC-Cy7	Tonbo	25-0038-T025	UCHT1	Mouse	IgG1 κ	AB_2621620	600	0.0083
	R8	CD27	APC-Fire 810	BioLegend	302863	O323	Mouse	IgG1 κ	AB_2894450	50	0.0083

N/A, not available.

### Antibody titration, spreading and fluorescence-minus-one controls

**Figure 1:**
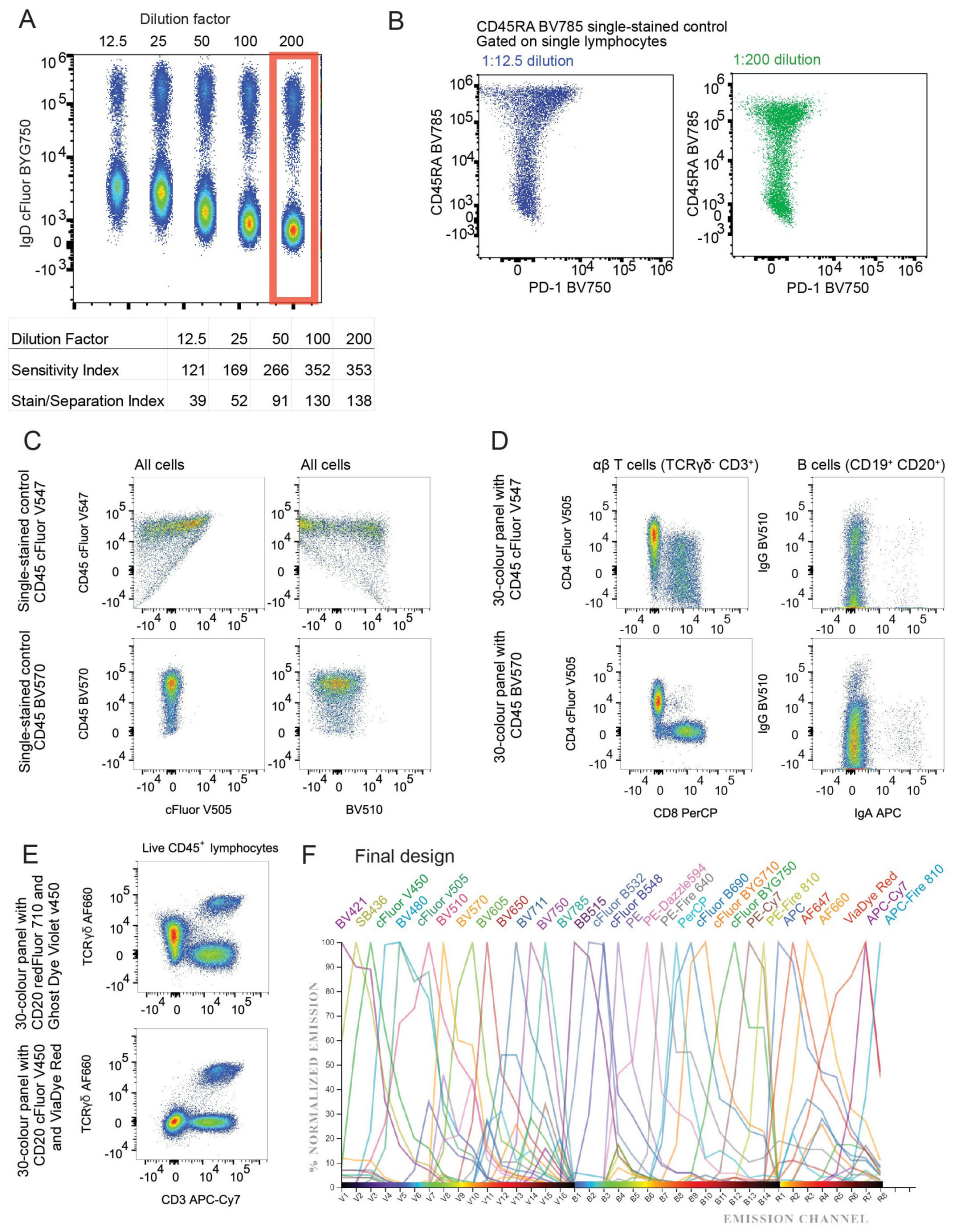
Design of 30 colour PBMC immunophenotyping panel: **(A)** Concatenated dot plot showing cells stained with increasing dilutions of IgD cFluor BYG750 and summary table of the sensitivity and stain indices calculated from this data. **(B)** Dot plots demonstrating spillover spreading error in an IgD cFluor BYG750 single-stained control, and the reduction of the error when diluted to 1:200. **(C)** Dot plots of single-stained controls demonstrating spreading error and skewing of the negative population caused by CD45 cFluor V547. Errors are not present in a single-stained control of CD45 BV570 antibody. **(D)** Improvement in CD4/CD8 and IgG/IgA population resolution in the fully stained sample when CD45 cFluor V547 was replaced with CD45 BV570. **(E)** Dot plot demonstrating spreading of the AF660 negative population caused by CD20 redFluor 710. This was corrected when CD20 was moved to cFluor v450. **(F)** Normalised emission spectra of the final panel design. generated using CYTEK Cloud (cloud.cytekbio.com/).

Antibodies were titrated to determine the concentration that would yield optimal staining characteristics, considering the following criteria: First, the MFI of positively stained events was compared to the MFI of negative events. Two slightly different metrics were calculated for each antibody concentration; stain/separation index (MFI-positive – MFI-negative/2 x SD-negative) (19) and sensitivity index (MFI-positive – MFI-negative/((84^th^ %-negative – MFI-negative)/0.995)) (20). The concentration producing the highest combined index has the best resolution between positive and negative events (**Figure 1A**). Second, this concentration was assessed to ensure consistent percentage of positive events within samples stained with higher antibody concentrations. Third, each concentration was assessed for detection in other channels.

The term *spillover-spreading error* refers to the spread of events visible after compensation or spectral unmixing has been applied to data (21). Ideally, the widths of the positive and negative populations should be the same, in order to achieve the highest possible sensitivity. After compensation, spillover-spreading error creates a positive population that spreads out in the shape of an umbrella, with wider spreading at events of higher fluorescence intensities. We assessed spreading by viewing plots of each single colour control against all other colours. At the optimal concentrations chosen from titration experiments, spillover-spreading error was minimal in most channels. In some colours, spreading was still noticeable. For example, **Figure 1B** shows CD45RA BV785 caused spreading into PD-1 BV750 when stained at a 1:12.5 dilution, but reduced spreading was observed when it was stained at a 1:200 dilution which reduced the MFI of positive events. Some antibody concentrations, such as CD45RA BV785, were chosen at a dilution factor that reduced this spreading error but still maintained the same positive event percentage. The dilution factor of antibodies in the final cocktail are listed in **Table 1**.

Despite using Ficoll-purified PBMCs, we opted to include CD45 in our panel to ensure the exclusive analysis of leukocytes. Our initial fluorophore choice for CD45 was cFluor V547. However, a substantial spreading error occurred into Super Bright 436, v450, BV480 and most noticeably, cFluor V505 and BV510 (**Figure 1C** top row), resulting in an obvious diagonal double-negative population. Recognising the challenge posed by such high spreading on a ubiquitous marker like CD45, we explored an alternative by testing CD45 on BV570 (Biolegend 304034). BV570 is also excited by the violet laser and peaks in the same detector (V8), but has a narrower emission peak. Minimal spreading into cFluor v505 and BV510 was observed (**Figure 1C** bottom row). Swapping the flurophore from cFluor V547 to BV570 reduced the calculated complexity index of the panel. In the fully-stained sample, this change improved population resolution of CD4^+^ and CD8^+^ T cells, and IgA^+^ and IgG^+^ B cells (**Figure 1D**).

During panel optimisation, we tested ViaDye Red (Cytek R0-00008) as another option for our viability dye, as there is low autofluorescence in the far red part of the spectrum. At the same time, we tested moving CD20 from redFluor 710 to cFluor v450 (Cytek R7-20015). This change improved the resolution of TCRγδ AF660 positive events (**Figure 1E**).

The resolution of cFluor R720 staining was negatively affected by spreading contributed by other fluorophores (**Supplementary Figure 1**). APC-Cy7, redFluor-710, BV711 and cFluor BYG750 are all contributing spread into the cFluor R720 channel. This made it difficult to detect real CD56 cFluor R720 positive events, which have an MFI of 1.2 x 10^4^ – barely higher than the events caused by spread. Due to space constraints in the panel, our decision was to remove CD56 from the panel entirely, and measure NK cells in future panel design attempts. This modification to the panel further reduced the calculated complexity index. The final 30-colour panel design (**Table 1**; **Figure 1F**), which is used throughout the remainder of our data, has a theoretical complexity index of 19.59 (CYTEK Cloud December 2024, **Supplementary Figure 2**). In practice, after acquisition of single-colour controls on SpectroFlo, this complexity index is reported to be approximately 23 (**Supplementary Figure 3**). This is significantly improved from the initial design with a theoretical complexity index of 139.31.

### Optimisation of staining procedure

**Figure 2:**
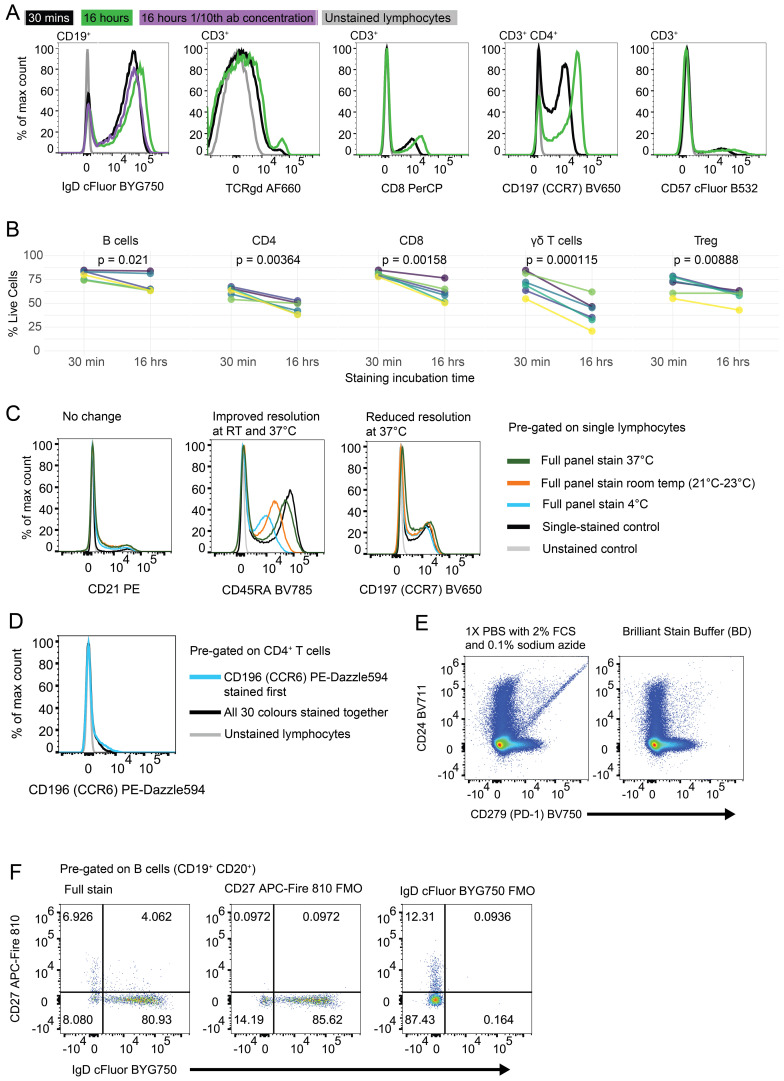
Optimisation of staining time, temperature and buffers: **(A)** Histograms of fully-stained PBMC populations which were incubated with antibody cocktail for 30 minutes (black line), 16 hours (green line) or 16 hours with 1/10^th^ of the antibody concentration (purple line). Unstained lymphocytes are shown in grey as a negative control. **(B)** Percentage of cells alive after 30 minutes or 16 hours of antibody staining. B cells (CD3^-^ CD19^+^ CD20^+^), CD4 T cells (CD3^+^ TCRγδ^-^ CD4^+^ CD8^-^), CD8 T cells (CD3^+^ TCRγδ^-^ CD4^-^ CD8^+^), γδ T cells (CD3^+^ TCRγδ^+^), Tregs (CD3^+^ TCRγδ^-^ CD4^+^ CD8^-^ CD127^-^ CD25^+^). p values produced by paired T test. Each coloured line represents the cells from a single healthy donor individual, repeated in both time conditions. **(C)** Histograms of fluorescence on single lymphocytes after PBMC samples were incubated with antibody cocktail at different temperatures; dark green: 37°C, orange: 21-23°C, blue: 4°C. Single stained control (black) and unstained control (grey) were incubated at 4°C. **(D)** Histogram of CD196 (CCR6) PE-Dazzle 594 fluorescence on CD4^+^ T cells. Blue line represents sample where CCR6 was stained in a primary layer and all other colours were stained in a secondary layer. Black line represents all 30 colours stained together in one layer. **(E)** Dot plots of Live CD45^+^ lymphocytes stained with Brilliant Violet fluorophores diluted in FACS wash (1X PBS with 2% FCS and 0.1% sodium azide) (left) or Brilliant Stain Buffer (BD) (right). Antibody cocktails were made in the listed buffer 2 hours before staining. Staining was performed for 30 minutes at 4°C. **(F)** Dot plots of CD19^+^ CD20^+^ B cells stained with all 30 colours (left plot), all except CD27 APC-Fire 810 (middle plot) or all except IgD cFluor BYG750 (right plot). Numbers represent frequency of events in the quadrant out of all CD19^+^ CD20^+^ B cells.

Extended antibody incubation times can improve resolution and sensitivity, while maintaining specificity, and concurrently reducing antibody costs and inter-experiment variability (22). To assess the impact of staining time on the resolution of our multicolour panel, we compared 30-minute incubation and 16-hour (overnight) incubation. We found improved resolution in signal from the majority of antibodies when cells were stained overnight (**Figure 2A**; **Supplementary Figure 4**). Notably, for IgD cFluor BYG750 reducing the antibody concentration to one-tenth of the “optimal” concentration when used in an overnight staining protocol, yielded comparable resolution to the 30-minute stain with the “optimal” concentration (**Figure 2A**). Other antibodies with improvement in resolution after overnight staining included TCRγδ AF660, CD8 PerCP, CD197 (CCR7) BV650 and CD57 cFluor B532 (**Figure 2A**). However, overnight staining resulted in a significant decrease in cell viability in both B and T cell populations (**Figure 2B**). We hypothesise this is due to the prior exposure of these cells to cryopreservation, including media with dimethyl sulfoxide, leaving cells more susceptible to death than the fresh murine cells used in previous studies (22). Prioritising cell viability, we opted to continue with the 30-minute staining procedure.

For cell fixation, we compared the eBioscience FoxP3 Transcription Factor Staining Buffer set (ThermoFisher 00-5523-00) to a solution of 1% paraformaldehyde in 1X PBS (ThermoFisher J19943.K2). This comparison resulted in two samples exhibiting distinct scatter properties on FSC and SSC (**Supplementary Figure 5**). This resulted in slightly different lymphocyte and monocyte frequencies. The paraformaldehyde-treated cells demonstrated a slightly higher percentage of B cells (17.27% compared to 16% in the kit-fixed sample) and lower percentage of T cells (62.86% compared to 64.54% in the kit-fixed sample). As 1% paraformaldehyde was used as a fixative in multiple recent OMIPs (23–25), and it did not appear to perform inferiorly to a commercial kit, we were confident to continue with this fixative.

To determine the optimal staining temperature, we evaluated three different methods; staining at 4°C (fridge), 21-23°C (RT), and 37°C (heated bead bath). All methods were tested using the same antibody cocktail, with 50 µL per well, and a 30-minute staining duration (**Figure 2C**; **Supplementary Figures 6**, **7**). Compared to samples stained at 4°C, those stained at RT exhibited no change in resolution in twelve antibodies (**Figure 2C** left plot), and improved resolution in thirteen out of the antibodies tested (**Figure 2C** middle plot). Staining at 37^°^C resulted in an increase of non-specific background staining in seven antibodies tested (**Figure 2C** right plot), and a slight decrease in viable cells (**Supplementary Figure 7**). We concluded that for this combination of antibodies, staining at RT is optimal.

Staining antibodies in sequential steps has been demonstrated to improve the resolution of some populations (24, 26). We found that three antibodies (TCRγδ AF660, CD196 (CCR6) PE-Dazzle 594, and CXCR5 BV421) showed slightly improved resolution when stained in a primary layer with a 30-minute incubation, and the remainder of the antibodies added in a secondary layer subsequently (**Figure 2D**; **Supplementary Figure 8**). We do not know if the improved resolution is due to the addition of the antibodies on their own or to the increased staining time.

Brilliant Stain Buffer (BD) was designed to limit aggregation of polymer dyes. To confirm that it improved detection of markers conjugated to Brilliant Violet fluorophores, we compared antibody cocktails prepared in Brilliant Stain Buffer versus FACS wash buffer (1X PBS with 2% FCS and 0.1% sodium azide). After 30 minutes of antibody staining in FACS wash, we observed Brilliant Violet antibody aggregates (**Figure 2E** left plot). However, if the cells were stained in Brilliant Stain Buffer, these aggregates were not observed (**Figure 2E** right plot). In addition to preventing aggregation, we aimed to reduce non-specific binding, which is known to occur with certain fluorophores, such as cyanine tandem dyes (27–29). We assessed monocytes for MFI of cyanine-conjugated antibodies in our panel; PE-Cy7, APC-Cy7, and cFluor B690 (likely cyanine-based due to its spectral similarity to PerCP-Cy5.5, although its precise structure is proprietary). PE-Cy7 and cFluor B690 both exhibited some background staining on monocytes, and APC-Cy7 showed minimal background staining (**Supplementary Figure 9**). Inclusion of True-Stain Monocyte Blocker (BioLegend) led to a modest reduction in background signal. Since it required no additional steps (it was added directly to the antibody cocktail) we incorporated this reagent in our antibody cocktail to improve specificity.

We also evaluated different Fc receptor blocking strategies to further reduce non-specific antibody binding. These were Human FC Block Pure Fc1 (BD #564220), Human TruStain FcX (Biolegend #422301), and purified Human IgG (Merck, #I4506) at 100µg/mL. When the staining of lymphocytes and monocytes was compared to a no blocking control, these reagents made minimal difference in non-specific binding (**Supplementary Figure 10**). As the Fc blocking step added an additional incubation period, we opted to proceed without incorporating an Fc block in the final protocol. We plan to reassess the use of Fc blocking in future panels.

### Population definition by manual gating

To accurately define positively stained populations and determine the placement of our manual gating strategy, we performed Fluorescence Minus One (FMO) control analysis. FMO controls apply all antibodies in the full stain except for one, which will display the full extent of negative spreading caused by the remainder of the panel. This allows an operator to set a gate that captures only true positive events. We created an FMO control for each antibody in the panel to define the entire gating strategy accurately (**Figure 2F**; **Supplementary Figure 11**). **Figure 2F** shows the FMO for CD27 and IgD, which often stain in a continuum rather than having clear negative and positive populations.

**Figure 3:**
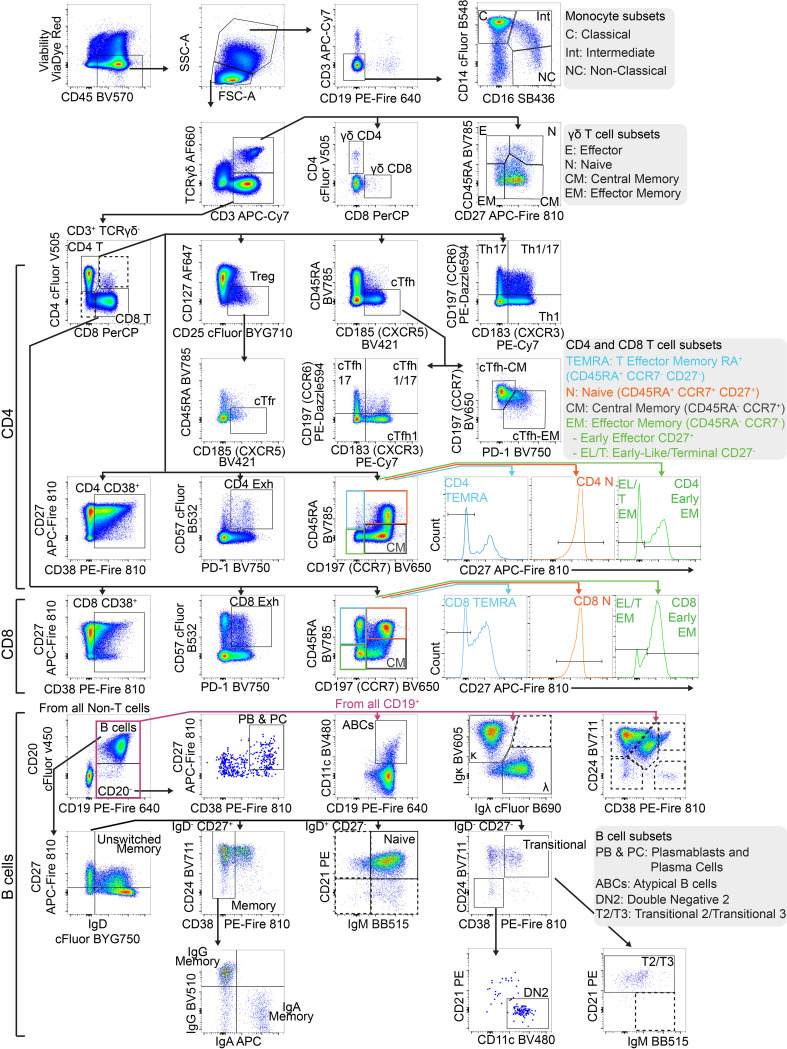
Gating strategy to identify T and B cell populations from PBMCs: Representative FACS plots of PBMCs from a single healthy blood donor. PBMCs were stained with the 30-colour panel, fixed with 1% paraformaldehyde, and acquired on a Cytek Northern Lights (3 Laser – V/B/R). Each fluorescent axis is biexponential with maximum of 2x10^6^ and scaling width basis -400. Scatter axes are linear. Gated populations are surrounded with solid boxes, while QC-only populations are surrounded by dotted-line boxes. Gated populations are as follows: Monocytes (FSC-A^mid^ SSC-A^high^ CD3^-^ CD19^-^) including Classical monocytes (CD14^+^ CD16^-^), Intermediate monocytes (CD14^+^ CD16^+^) and non-classical monocytes (CD14^-^ CD16^+^), Lymphocytes (FSC-A^mid^ SSC-A^low^). γδ T cells (TCRγδ^+^ CD3^+^) including CD4^+^, γδ CD8^+^, γδ Effector (CD45RA^+^ CD27^-^), γδ Naïve (CD45RA^+^ CD27^+^), γδ Central Memory (CD45RA^-^ CD27^+^), γδ Effector Memory (CD45RA^-^ CD27^-^). αβ T cells (TCRγδ^-^ CD3^+^) including CD4^+^ T cells, CD8^+^ T cells, CD4 Tregs (CD127^-^ CD25^+^), cTfr (CD45RA^-^ CXCR5^+^), CD4 Th subsets Th17 (CCR6^+^ CXCR3^-^), Th1/17 (CCR6^+^ CXCR3^+^), Th1 (CCR6^-^ CXCR3^+^), double negative CCR6^-^ CXCR3, CD4 cTfh (CD45RA^-^ CXCR5^+^) (also subdivided into Th1/Th17/Th1/17 subsets by CCR6 and CXCR3), CD4 cTfh Central Memory (CCR7^+^ PD-1^-^), CD4 cTfh Effector Memory (CCR7^mid^ PD-1^+^), CD4 CD38^+^ activated T cells, CD4 Exhausted (CD57^+^ PD-1^+^), CD4 TEMRA (CD45RA^-^ CCR7^-^ CD27^-^), CD4 Naïve (CD45RA^+^ CCR7^+^ CD27^+^), CD4 Central Memory (CD45RA^-^ CCR7^+^) and CD4 Effector Memory (CD45RA^-^ CCR7^-^), CD4 Early Effector Memory (CD27^+^), CD4 Early-like/Terminal Effector Memory (CD27^-^), CD8 CD38^+^ activated T cells, CD8 Exhausted (CD57^+^ PD-1^+^), CD8 TEMRA (CD45RA^-^ CCR7^-^ CD27^-^), CD8 Naïve (CD45RA^+^ CCR7^+^ CD27^+^), CD8 Central Memory (CD45RA^-^ CCR7^+^) and CD8 Effector Memory (CD45RA^-^ CCR7^-^ CD27^-^), CD8 Early Effector Memory (CD27^+^), CD8 Early-like/Terminal Effector Memory (CD27^-^). From CD19^+^ CD20^-^ cells, Plasmablasts and Plasma Cells (PB & PC) were identified (CD27^+^ CD38^+^). B cells (CD19^+^ CD20^+^) including Memory (CD27^+^ IgD^-^ CD24 var CD38^-^), IgG Memory (IgG^+^ IgA^-^), IgA Memory (IgG^-^ IgA^+^), IgG-IgA Double Negative Memory (IgG^-^ IgA^-^), Unswitched Memory (CD27^+^ IgD^+^), Naïve Resting (CD27^-^ IgD^+^ CD21^+^ IgM^var^), Transitional (CD27^-^ IgD^-^ CD24^+^ CD38^+^), Transitional 2/3 (CD21^+^ IgM^var^). Total CD19^+^ cells included Atypical B cells (ABCs) (CD19^+^ CD11c^+^), and light-chain subsets [Ig-κ light chain (Igκ^+^ Igλ^-^), Ig-λ light chain (Igκ^-^ Igλ^+^)].

Our manual gating strategy applied to a fully-stained sample of PBMCs from a healthy donor is shown in **Figure 3**. Preliminary data cleaning was performed on the unmixed FCS file in order to remove doublets, red blood cell (RBC) contamination (see (30, 31) for discrimination of RBCs using side-scatter), antibody aggregates and debris (**Supplementary Figure 12**). Live CD45^+^ (leukocytes) were gated via Viability Dye^-^ CD45^+^ (**Figure 3**). If a sample had less than 10,000 events in the Live CD45^+^ gate, or this population was less than 15% of the total events, then the sample was deemed too low-quality to continue through analysis. FSC-A^mid^, SSC-A^high^ cells were then gated on CD3^-^ CD19^-^ to select monocytes, which were separated into classical monocytes (CD14^+^ CD16^-^), intermediate monocytes (CD14^+^ CD16^+^) and non-classical monocytes (CD14^-^ CD16^+^). Monocyte populations are listed in **Table 2**.

**Table 2:** Monocyte populations gated in this panel.

Population	Gating strategy (all pre-gated on Live CD45+)
Monocytes	FSC-A mid SSC-A high/CD3- CD19-
Classical Monocytes	FSC-A mid SSC-A high/CD3- CD19-/CD14+ CD16-
Intermediate Monocytes	FSC-A mid SSC-A high/CD3- CD19-/CD14+ CD16+
Non-Classical Monocytes	FSC-A mid SSC-A high/CD3- CD19-/CD14- CD16+

FSC-A^mid^ SSC-A^low^ cells were designated as lymphocytes. γδ T cells were identified (TCRγδ^+^ CD3^+^) and subpopulations were further separated, including γδ CD4^+^, γδ CD8^+^, γδ Effector (CD45RA^+^ CD27^-^), γδ Naïve (CD45RA^+^ CD27^+^), γδ Central Memory (CD45RA^-^ CD27^+^) and γδ Effector Memory (CD45RA^-^ CD27^-^). αβ T cells (identified by TCRγδ^-^ CD3^+^) were separated into CD4^+^ and CD8^+^.

CD4^+^ T cells were subdivided into T regulatory cells (Tregs) (CD127^-^ CD25^+^) and the subpopulation circulating T follicular regulatory cells (cTfr) (CD45RA^-^ CXCR5^+^), Th subsets Th17 (CCR6^+^ CXCR3^-^), Th1/17 (CCR6^+^ CXCR3^+^), Th1 (CCR6^-^ CXCR3^+^), and double negative CCR6^-^ CXCR3^-^, circulating T follicular helper cells (cTfh) (CD45RA^-^ CXCR5^+^) (which were also subdivided into Th1/Th17/Th1/17 helper subsets by CCR6 and CXCR3), cTfh Central Memory (CCR7^+^ PD-1^-^) and cTfh Effector Memory (CCR7^mid^ PD-1^+^), CD4 CD38^+^ activated T cells, CD4 Exhausted (CD57^+^ PD-1^+^), CD4 TEMRA (CD45RA^-^ CCR7^-^ CD27^-^), CD4 Naïve (CD45RA^+^ CCR7^+^ CD27^+^), CD4 Central Memory (CD45RA^-^ CCR7^+^) and CD4 Effector Memory (CD45RA^-^ CCR7^-^) (which were separated into Early Effector Memory and Early-like/Terminal Effector Memory based on positive or negative CD27 expression respectively).

Using the same marker expression, CD8^+^ T cells were separated into CD8 CD38^+^ activated T cells, CD8 Exhausted (CD57^+^ PD-1^+^), CD8 TEMRA (CD45RA^-^ CCR7^-^ CD27^-^), CD8 Naïve (CD45RA^+^ CCR7^+^ CD27^+^), CD8 Central Memory (CD45RA^-^ CCR7^+^) and CD8 Effector Memory (CD45RA^-^ CCR7^-^) (with Early CD27^+^ and Early-Like/Terminal CD27^-^ subpopulations). T cell populations are listed in **Table 3**.

**Table 3:** T cell populations gated in this panel.

Population	Gating strategy (all pre-gated on Live CD45+/Lymphocytes)
Total T cells	CD3+
γδ T cells	CD3+ γδTCR+
γδ CD4	CD3+ γδTCR+/CD4+ CD8-
γδ CD8	CD3+ γδTCR+/CD4- CD8+
γδ Central Memory	CD3+ γδTCR+/CD45RA- CD27+
γδ Effector	CD3+ γδTCR+/CD45RA+ CD27-
γδ Effector Memory	CD3+ γδTCR+/CD45RA- CD27-
γδ Naïve	CD3+ CD19-/γδTCR+/CD45RAhi CD27hi
αβ T cells	CD3+ γδTCR-
CD4 T cells	CD3+ γδTCR-/CD4+ CD8-
CD4 Naïve	CD3+ γδTCR-/CD4+ CD8-/CD45RA+ CCR7+/CD27+
CD4 TEMRA	CD3+ γδTCR-/CD4+ CD8-/CD45RA+ CCR7-/CD27-
CD4 Central Memory	CD3+ γδTCR-/CD4+ CD8-/CD45RA- CCR7+
CD4 Effector Memory	CD3+ γδTCR-/CD4+ CD8-/CD45RA- CCR7-
CD4 Early Effector Memory	CD3+ γδTCR-/CD4+ CD8-/CD45RA- CCR7-/CD27+
CD4 Early-like/Terminal Effector Memory	CD3+ γδTCR-/CD4+ CD8-/CD45RA- CCR7-/CD27-
CD4 Exhausted	CD3+ γδTCR-/CD4+ CD8-/PD1+ CD57+
CD4 CD38^+^ (activated)	CD3+ γδTCR-/CD4+ CD8-/CD38+
Treg	CD3+ γδTCR-/CD4+ CD8-/CD127- CD25+
cTfr	CD3+ γδTCR-/CD4+ CD8-/CD127- CD25+/CD45RA- CXCR5+
Th17	CD3+ γδTCR-/CD4+ CD8-/CCR6+ CXCR3-
Th1	CD3+ γδTCR-/CD4+ CD8-/CCR6- CXCR3+
Th1/Th17	CD3+ γδTCR-/CD4+ CD8-/CCR6+ CXCR3+
CCR6- CXCR3-	CD3+ γδTCR-/CD4+ CD8-/CCR6- CXCR3-
cTfh	CD3+ γδTCR-/CD4+ CD8-/CD45RA- CXCR5+
cTfh Th17	CD3+ γδTCR-/CD4+ CD8-/CD45RA- CXCR5+/CCR6+ CXCR3-
cTfh Th1	CD3+ γδTCR-/CD4+ CD8-/CD45RA- CXCR5+/CCR6- CXCR3+
cTfh Th1/Th17	CD3+ γδTCR-/CD4+ CD8-/CD45RA- CXCR5+/CCR6+ CXCR3+
cTfh CCR6- CXCR3-	CD3+ γδTCR-/CD4+ CD8-/CD45RA- CXCR5+/CCR6- CXCR3-
cTfh Central Memory	CD3+ γδTCR-/CD4+ CD8-/CD45RA- CXCR5+/CCR7+ PD-1-
cTfh Effector Memory	CD3+ γδTCR-/CD4+ CD8-/CD45RA- CXCR5+/CCR7mid PD-1+
CD8 T cells	CD3+ γδTCR-/CD4- CD8+
CD8 Exhausted	CD3+ γδTCR-/CD4- CD8+/PD1+ CD57+
CD8 Naïve	CD3+ γδTCR-/CD4- CD8+/CD45RA+ CCR7+/CD27+
CD8 TEMRA	CD3+ γδTCR-/CD4- CD8+/CD45RA+ CCR7-/CD27-
CD8 Central Memory	CD3+ γδTCR-/CD4- CD8+/CD45RA- CCR7+
CD8 Effector Memory	CD3+ γδTCR-/CD4- CD8+/CD45RA- CCR7-
CD8 Early Effector Memory	CD3+ γδTCR-/CD4- CD8+/CD45RA- CCR7-/CD27+
CD8 Early-like/Terminal Effector Memory	CD3+ γδTCR-/CD4- CD8+/CD45RA- CCR7-/CD27-
CD8 CD38^+^ (activated)	CD3+ γδTCR-/CD4- CD8+/CD38+

CD19 B cells were separated into a number of populations before CD20 was incorporated as an additional B cell lineage marker. CD19^+^ B cell subsets included Atypical B cells (ABCs) using CD11c as the definitive marker (32), and light-chain defined populations (Ig-κ light chain (Igκ^+^ Igλ^-^), Ig-λ light chain (Igκ^-^ Igλ^+^)). From CD19^+^ CD20^-^ cells, Plasmablasts and Plasma Cells (PB & PC) were identified (CD27^+^ CD38^+^).

From CD19^+^ CD20^+^ B cells, developmental stage was determined using CD27 and IgD which were gated by additional markers to confirm as follows; Memory (CD27^+^ IgD^-^ CD24^var^ CD38^-^), Unswitched Memory (CD27^+^ IgD^+^), Naïve Resting (CD27^-^ IgD^+^ CD21^+^ IgM^var^), and Transitional (CD27^-^ IgD^-^ CD24^+^ CD38^+^). Memory B cells were separated by surface immunoglobulin class (IgG^+^, IgA^+^ or IgG^-^ & IgA^-^). Transitional B cells were further gated down to T2/T3 (CD21^+^ IgM^var^). Double Negative 2 (DN2) B cells were identified by CD27^-^ IgD^-^ CD38^-^ CD24^-^ CD21^-^ CD11c^+^ (33). B cell populations are listed in **Table 4**.

**Table 4:** B cell populations gated in this panel.

Population	Gating strategy (all pre-gated on Live CD45+/Lymphocytes/Not (T cell))
Total B cells (inc. Plasmablasts and Plasma Cells)	CD19+
Ig-κ light chain	CD19+/Ig-κ+
Ig-λ light chain	CD19+/Ig- λ+
Atypical B cells	CD19+/CD11c+
Plasmablasts and Plasma Cells	CD19+ CD20-/CD38+ CD27+
Memory B cells	CD19+ CD20+/CD27+ IgD-/CD38-
IgA Memory	CD19+ CD20+/CD27+ IgD-/CD38-/IgA+
IgG Memory	CD19+ CD20+/CD27+ IgD-/CD38-/IgG+
Double Negative Memory	CD19+ CD20+/CD27+ IgD- CD38-/IgA- IgG-
Naïve Resting	CD19+ CD20+/CD27- IgD+/CD21+ IgMvar
Transitional	CD19+ CD20+/CD27- IgD-/CD38+ CD24+
T2 and T3	CD19+ CD20+/CD27- IgD- CD38+ CD24+/CD21+ IgMvar
Unswitched Memory	CD19+ CD20+/CD27+ IgD+
Double Negative 2 (DN2)	CD19+ CD20+/CD27- IgD-/CD38- CD24-/CD21- CD11c+

Additionally, in manual gating analysis we included some populations purely for quality assurance (denoted by dotted gating lines in **Figure 3**). In some cases, these are populations with low frequency in healthy people (e.g. double positive CD4^+^ CD8^+^ T cells, CD21^-^ Naïve B cells). Some of these populations are generally undetectable in healthy people (e.g. dual-light-chain expressing B cells (Igκ^+^ Igλ^+^), T1 B cells (CD27^-^ IgD^-^ CD24^+^ CD38^+^ CD21^-^ IgM^+^). Some are biologically unlikely, such as double positive IgG^+^ IgA^+^ memory B cells. These populations were used to assess for aberrant expansion (either due to biology or experimental error) which could then be investigated. Additionally, we also maintained an additional set of B cell gates measuring major populations by CD24 and CD38 expression (Memory (CD24^hi^ CD38^-^), Naïve (CD24^mid^ CD38^mid^), Transitional (CD24^hi^ CD38^hi^), Plasmablasts (CD24^-^ CD38^hi^), Anergic (CD24^-^ CD38^-^)) for a layer of redundancy.

To ensure that only reliable results were reported, throughout manual analysis, if a gated population had less than 100 events, then no frequency value for that population or any of its subpopulations was reported. This number was chosen after we compared the co-efficient of variation (CV) of all the subsets at various cut-off levels and found that this level gave the best results for capturing biological variability and minimising analytical variability introduced by the assay itself.

### Panel performance over time

**Figure 4:**
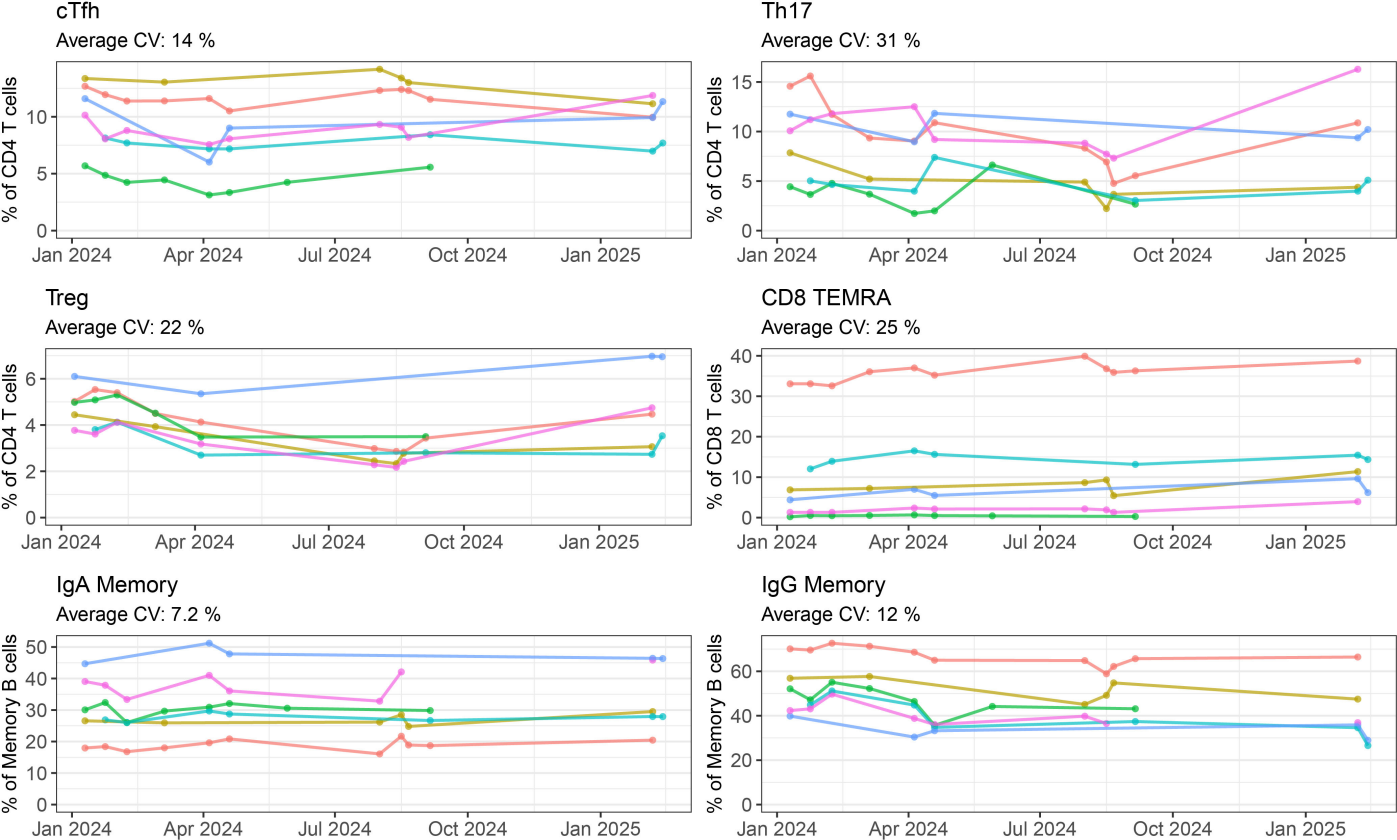
Batch variability of repeated healthy PBMCs by manual gating: Frequency of six selected populations (cTfh, Th17, Treg, CD8 TEMRA, IgA Memory, IgG Memory) measured for six healthy controls over five or more batch repeats. Each coloured line represents one individual, with the cells coming from the same batch of PBMCs frozen in late 2023. Where a point is not shown either that individual’s sample was not included in the run, or the quality requirements for inclusion were not met.

To determine how batch effects change the numerical output of our panel, frequency of lymphocyte subpopulations were measured on repeated samples. For six healthy donors, we collected PBMCs in late 2023, and then used one vial of PBMCs in repeated batches over the span of one year. In six selected populations (**Figure 4**, all populations shown in **Supplementary Figure 13** and **Supplementary Table 2**) we can see there is some variation between batches. These tended to vary around a mean value. In the case of CD4 Tregs, we noticed a decline in frequency in mid-2024. This occured due to a decrease in fluorescence intensity of our CD25 cFluor BYG710 antibody, which is the main positive marker for Tregs in our panel. Once we opened a new vial of antibody, the frequency of Tregs somewhat recovered (**Figure 4**). We are therefore replacing it more frequently to account for this decrease in fluorescence intensity. We have also identified CD25 RB705 (BD 570249) as a potential replacement for this antibody which has a similar emission spectra to cFluor BYG710, and can be substituted into our panel.

**Figure 5:**
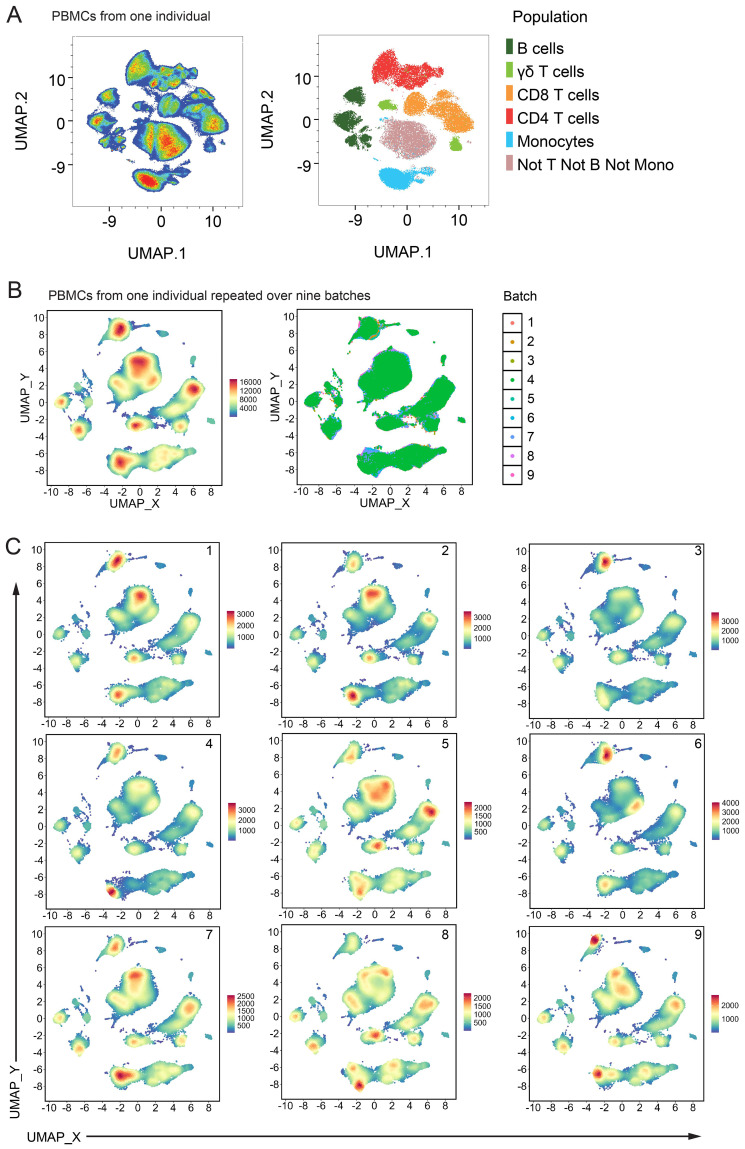
Batch variability from unsupervised analysis: **(A)** UMAP of Live CD45^+^ cells from one healthy control individual. Left plot is coloured by cell density and right plot shows the same events coloured by their manually gated population. **(B)** UMAP of Live CD45^+^ cells pooled from nine batch repeats of one healthy control individual. 50,000 events from each batch were used. UMAP settings; Distance: Euclidean, NN: 15, Min dist: 0.5. Left plot is coloured by cell density and right plot shows the same events coloured by their batch run. **(C)** Same UMAP shown in **(B)** with each batch shown on one panel, coloured by cell density.

To measure batch effects occurring outside of our manual gating strategy, we performed dimensionality reduction using UMAP (**Figure 5**) on Live CD45^+^ events from a healthy control individual. When the global structure was overlayed with colours representative of the manually gated populations (CD4 and CD8 T cells, γδ T cells, B cells, monocytes and all other cells) the major lineage populations separated into distinct islands (**Figure 5A**, right plot). These lineage population islands were made up of their relevant subpopulations (**Supplementary Figure 14**). One healthy control PBMC sample was repeated over nine batches, and the pooled data were analysed by UMAP (**Figure 5B**). The same major lineage populations were separated as distinct islands with expected marker expression patterns (**Supplementary Figure 15**). When coloured by batch run (**Figure 5B** right plot), or visualised on separate plots (**Figure 5C**), slight variability between runs was visible but largely, global structure was consistent between all nine batches.

### Healthy adults

**Figure 6:**
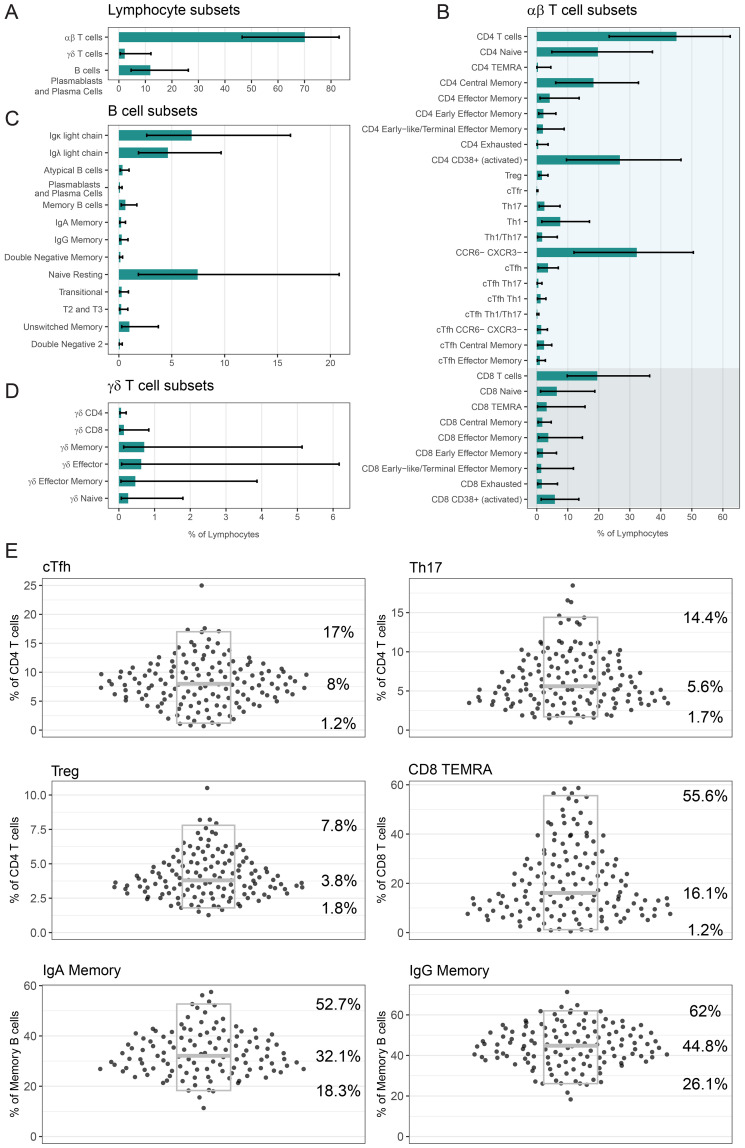
Analysis of lymphocyte populations in our healthy cohort: Frequency of **(A)** αβ T cells, γδ T cells, B cells PB & PC, **(B)** αβ T cell subsets, **(C)** B cell subsets and **(D)** γδ T cell subsets as a percentage of lymphocytes. In **(A-D)** bars indicate median percentage, and error bars designate 2.5^th^ percentile and 97.5^th^ percentile. **(E)** Frequency of six selected populations (cTfh, Th17, Treg, CD8 TEMRA, IgA Memory, IgG Memory) as a percentage of relevant parent population. Each point is a unique individual. Grey boxes represent the median (middle line marker), 2.5^th^ percentile (lower bound) and 97.5^th^ percentile (upper bound), and the values of these percentiles are displayed on the right. For five populations, there were several people who had less than 100 events in this population and as such we did not report a frequency; cTfh: 2, Th17: 1, CD8 TEMRA: 1, IgA Memory: 29, IgG Memory: 16. In **(A-E)** the number of healthy donors n = 148, with the exception of Treg n = 138. If a person’s PBMCs were repeated over multiple batches, then only the data from their most representative batch in displayed and used to calculate summary statistics.

Once we were satisfied with the performance of our panel, we analysed the PBMCs of 148 healthy adults. The median age of this cohort was 50 years (range 18-83, age of 12 participants not reported). The cohort was comprised of 58% female and 42% male participants (sex of 6 participants not reported). **Figure 6** presents the frequency of lymphocyte subsets within this healthy population. On average, αβ T cells accounted for 70.1% of lymphocytes, γδ T cells for 2.2%, and B cells, PB & PC for 11.9% (**Figure 6A**). CD4 T cells comprised 45% of lymphocytes, while CD8 T cells comprised 19.5% (**Figure 6B**). The majority of B cells were classified as Naïve Resting B cells, representing 7.4% of lymphocytes (**Figure 6C**). γδ T cell subset distributions showed considerable inter-individual variability, with a wide range between the 2.5^th^ and 97.5^th^ percentiles (**Figure 6D**).

In **Figure 6E** we display the frequency data for six selected populations as a percentage of a relevant parent population, with individual donor values shown to illustrate the distribution across the healthy cohort (all populations in **Supplementary Figure 16**). As a frequency of CD4 T cells, cTfh cells had a median of 8% (2.5^th^ percentile – 97.5^th^ percentile; 1.2%–17%), Th17 cells had a median of 5.6% (1.7%–14.4%), and Tregs had a median of 3.8% (1.8%–7.8%). CD8 TEMRA cells had a median frequency of 16.1% (1.2%–55.6%) of CD8 T cells. As a frequency of Memory B cells, IgA Memory had a median of 32.1% (18.3%–52.7%) and IgG Memory had a median of 44.8% (26.1%–62%). Treg measurement was impacted by antibody performance for 10 individuals, and as such the Treg data (and cTfr data) is available for 138 donors only. For five of these six populations, there were some people who had too few (less than 100) gated events, and a frequency was not reported (cTfh: 2, Th17: 1, CD8 TEMRA: 1, IgA Memory: 29, IgG Memory: 16).

Summary statistics for the frequency of all lymphocyte and monocyte populations are shown in **Table 5**. These data capture the heterogeneity of this healthy population and provides a robust baseline for our future clinical validation.

**Table 5:** Frequency of lymphocyte and monocyte populations in healthy Australian adults.

Population	Measurement	Number of people with detectable population (number below limit of detection)	Median frequency (2.5th - 97.5th percentile)
αβ T cells	% of Lymphocytes	148 (0)	70.1 (46.5 - 83.1)
CD4 T cells	% of Lymphocytes	148 (0)	45 (23.3 - 62.4)
	% of αβ T cells	148 (0)	65.6 (40.1 - 81.1)
CD4 Naïve	% of Lymphocytes	148 (0)	19.7 (4.9 - 37.3)
	% of CD4 T cells	148 (0)	43.3 (13 - 72.6)
CD4 TEMRA	% of Lymphocytes	92 (56)	0.4 (0 - 4.5)
	% of CD4 T cells	92 (56)	0.9 (0.1 - 10.9)
CD4 Central Memory	% of Lymphocytes	148 (0)	18.3 (6.1 - 32.8)
	% of CD4 T cells	148 (0)	40.2 (17 - 65.6)
CD4 Effector Memory	% of Lymphocytes	148 (0)	4.2 (1.1 - 13.7)
	% of CD4 T cells	148 (0)	10.8 (2 - 31.8)
CD4 Early Effector Memory	% of Lymphocytes	148 (0)	2.2 (0.6 - 6.2)
	% of CD4 Effector Memory	148 (0)	56.4 (21.2 - 80.2)
CD4 Early-like/Terminal Effector Memory	% of Lymphocytes	148 (0)	2 (0.3 - 8.8)
	% of CD4 Effector Memory	148 (0)	43.6 (19.8 - 78.8)
CD4 Exhausted	% of Lymphocytes	133 (15)	0.5 (0.1 - 3.7)
	% of CD4 T cells	133 (15)	1.1 (0.1 - 9.3)
CD4 CD38+ (activated)	% of Lymphocytes	148 (0)	26.8 (9.6 - 46.5)
	% of CD4 T cells	148 (0)	59.8 (30.7 - 83.2)
Treg	% of Lymphocytes	138 (0)	1.7 (0.7 - 3.6)
	% of CD4 T cells	138 (0)	3.8 (1.8 - 7.8)
cTfr	% of Lymphocytes	85 (53)	0.1 (0 - 0.4)
	% of Treg	85 (53)	6.3 (3.5 - 16.2)
Th17	% of Lymphocytes	147 (1)	2.5 (0.8 - 7.5)
	% of CD4 T cells	147 (1)	5.6 (1.7 - 14.4)
Th1	% of Lymphocytes	147 (1)	7.6 (1.6 - 17)
	% of CD4 T cells	147 (1)	17.8 (5.4 - 38.5)
Th1/Th17	% of Lymphocytes	141 (7)	1.7 (0.3 - 6.6)
	% of CD4 T cells	141 (7)	4.4 (0.7 - 14.4)
CCR6- CXCR3-	% of Lymphocytes	148 (0)	32.2 (12 - 50.5)
	% of CD4 T cells	148 (0)	71.2 (43.6 - 90.8)
cTfh	% of Lymphocytes	146 (2)	3.6 (0.5 - 6.9)
	% of CD4 T cells	146 (2)	8 (1.2 - 17)
cTfh Th17	% of Lymphocytes	128 (20)	0.5 (0.1 - 1.7)
	% of cTfh	128 (20)	15.3 (3.8 - 31.8)
cTfh Th1	% of Lymphocytes	138 (10)	1.2 (0.2 - 3)
	% of cTfh	138 (10)	33.8 (20.4 - 55)
cTfh Th1/Th17	% of Lymphocytes	107 (41)	0.2 (0.1 - 0.7)
	% of cTfh	107 (41)	5.6 (1.4 - 12.4)
cTfh CCR6- CXCR3-	% of Lymphocytes	143 (5)	1.4 (0.3 - 3.4)
	% of cTfh	143 (5)	44.7 (24.4 - 72.7)
cTfh Central Memory	% of Lymphocytes	143 (5)	2.4 (0.3 - 4.8)
	% of cTfh	143 (5)	68.7 (30.8 - 86.8)
cTfh Effector Memory	% of Lymphocytes	139 (9)	1 (0.2 - 2.8)
	% of cTfh	139 (9)	29.3 (11 - 65.2)
CD8 T cells	% of Lymphocytes	148 (0)	19.5 (9.8 - 36.4)
	% of αβ T cells	148 (0)	29.3 (15.7 - 52.1)
CD8 Naïve	% of Lymphocytes	148 (0)	6.5 (1.2 - 18.7)
	% of CD8 T cells	148 (0)	34.6 (6 - 81.2)
CD8 TEMRA	% of Lymphocytes	147 (1)	3.2 (0.2 - 15.6)
	% of CD8 T cells	147 (1)	16.1 (1.2 - 55.6)
CD8 Central Memory	% of Lymphocytes	147 (1)	1.8 (0.4 - 4.7)
	% of CD8 T cells	147 (1)	9.4 (1.9 - 25.1)
CD8 Effector Memory	% of Lymphocytes	148 (0)	3.7 (0.6 - 14.7)
	% of CD8 T cells	148 (0)	20.5 (3.1 - 47.5)
CD8 Early Effector Memory	% of Lymphocytes	147 (1)	2 (0.3 - 6.4)
	% of CD8 Effector Memory	147 (1)	57.3 (23.1 - 84.9)
CD8 Early-like/Terminal Effector Memory	% of Lymphocytes	145 (3)	1.4 (0.2 - 11.8)
	% of CD8 Effector Memory	145 (3)	43.1 (15.1 - 77.1)
CD8 Exhausted	% of Lymphocytes	147 (1)	1.6 (0.3 - 6.7)
	% of CD8 T cells	147 (1)	8.2 (1.5 - 31.3)
CD8 CD38+ (activated)	% of Lymphocytes	148 (0)	5.8 (1.4 - 13.6)
	% of CD8 T cells	148 (0)	31.2 (7.8 - 59.7)
γδ T cells	% of Lymphocytes	148 (0)	2.2 (0.5 - 12.1)
γδ CD4	% of Lymphocytes	63 (85)	0.1 (0 - 0.2)
	% of γδ T cells	63 (85)	2.4 (0.3 - 9.8)
γδ CD8	% of Lymphocytes	83 (65)	0.1 (0 - 0.8)
	% of γδ T cells	83 (65)	5.4 (0.8 - 35.5)
γδ Central Memory	% of Lymphocytes	139 (9)	0.7 (0.1 - 5.1)
	% of γδ T cells	139 (9)	38.3 (6.1 - 67.3)
γδ Effector	% of Lymphocytes	130 (18)	0.6 (0.1 - 6.2)
	% of γδ T cells	130 (18)	25.9 (4 - 81.8)
γδ Effector Memory	% of Lymphocytes	123 (25)	0.5 (0.1 - 3.9)
	% of γδ T cells	123 (25)	17.4 (2.8 - 49.2)
γδ Naïve	% of Lymphocytes	126 (22)	0.3 (0.1 - 1.8)
	% of γδ T cells	126 (22)	12.7 (1.7 - 50.6)
Total B cells (inc. Plasmablasts and Plasma Cells)	% of Lymphocytes	148 (0)	11.9 (4.6 - 26.2)
Ig-κ light chain	% of Lymphocytes	148 (0)	6.9 (2.6 - 16.2)
	% of Total B cells	148 (0)	58.6 (52.3 - 65.9)
Ig-λ light chain	% of Lymphocytes	148 (0)	4.6 (1.9 - 9.7)
	% of Total B cells	148 (0)	38.9 (30.8 - 45)
Atypical B cells	% of Lymphocytes	140 (8)	0.3 (0.1 - 1)
	% of Total B cells	140 (8)	3 (1.1 - 7.8)
Plasmablasts and Plasma Cells	% of Lymphocytes	40 (108)	0.1 (0 - 0.3)
B cells	% of Lymphocytes	148 (0)	11.5 (4.5 - 26)
Memory B cells	% of Lymphocytes	143 (5)	0.6 (0.2 - 1.7)
	% of B cells	143 (5)	4.9 (1.7 - 14.8)
IgA Memory	% of Lymphocytes	119 (29)	0.2 (0.1 - 0.6)
	% of Memory B cells	119 (29)	32.1 (18.3 - 52.7)
IgG Memory	% of Lymphocytes	132 (16)	0.3 (0.1 - 0.9)
	% of Memory B cells	132 (16)	44.8 (26.1 - 62)
Double Negative Memory	% of Lymphocytes	112 (36)	0.1 (0.1 - 0.3)
	% of Memory B cells	112 (36)	21.4 (9.7 - 43.4)
Naïve Resting	% of Lymphocytes	148 (0)	7.4 (1.8 - 20.8)
	% of B cells	148 (0)	66 (33.2 - 83.4)
Transitional	% of Lymphocytes	135 (13)	0.3 (0.1 - 0.9)
	% of B cells	135 (13)	2.1 (0.6 - 6.9)
T2 and T3	% of Lymphocytes	131 (17)	0.2 (0.1 - 0.8)
	% of Transitional B cells	131 (17)	88.1 (59.8 - 98.2)
Unswitched Memory	% of Lymphocytes	145 (3)	1 (0.3 - 3.7)
	% of B cells	145 (3)	8.3 (1.9 - 30.3)
Double Negative 2	% of Lymphocytes	44 (104)	0.1 (0 - 0.3)
	% of B cells	44 (104)	0.6 (0.2 - 2.9)
Monocytes	% of Live CD45+	146 (2)	6.7 (0.8 - 27.7)
Classical Monocytes	% of Monocytes	146 (2)	77.5 (31.1 - 92.1)
Intermediate Monocytes	% of Monocytes	123 (25)	4.5 (1.4 - 14.2)
Non-Classical Monocytes	% of Monocytes	123 (25)	4.3 (0.6 - 16.4)

## Discussion

We developed a comprehensive T and B cell immunophenotyping panel, which operates on a 3-laser CYTEK Northern Lights. Spectral flow cytometry panels detecting leukocytes populations have previously been reported, including a 24-colour (17) and 22-colour panel (34). Recently a 31-colour panel using the 3-laser CYTEK Aurora instrument was reported (35), however our panel is quite distinct. Hammerich and colleagues (35) used fresh blood as their sample type, and primarily focused on gating monocytes, NK cells, and T cell populations. In contrast, our panel is optimised for use on cryopreserved PBMCs and additionally includes detailed characterisation of B cell populations, with anticipated utility in the diagnosis and monitoring of rheumatic disorders (36). Finally, the 31-colour panel has a theoretical complexity index of 55.34, whereas our 30-colour panel achieves a theoretical complexity index of 19.59 (and in practice, 23). This lower complexity index suggests that our fluorophore combinations may offer improved performance in reducing spectral overlap. We have achieved this high resolution panel on a 3-laser instrument, which is a more affordable option than the 4- or 5-laser setups used in other recent spectral panels for lymphocyte subset detection (24, 37).

One limitation of our panel is that we were not able to include the enumeration of NK cell subsets due to issues with the CD56 antibody described earlier, but plan to include this in a complementary second stain specifically designed for the 3-laser CYTEK Aurora. This means that we cannot negatively gate on CD56^+^ cells in our monocyte gate, like we can exclude CD3+ and CD19^+^ positive events and so the presence of NK cells in non-classical monocyte gate cannot be excluded.

We have collected data on 148 healthy blood donors, representing a substantial healthy cohort that is not commonly reported in similar studies. The donors were recruited in Canberra, which by population census data reflects the cultural diversity of the wider Australian population (38). Given Australia’s multicultural population, researchers comparing their data to ours should consider potential population-specific differences, as genetic and environmental diversity may influence immunophenotypes (39). While reference ranges were not calculated in this study, these data will contribute to the development of regional reference ranges for lymphocyte populations derived from PBMCs in healthy adults. Our healthy population provides a robust baseline for future research studies and clinical validation efforts with our aim to have a panel accredited under ISO15189 so that the results can be used directly in clinical care.

There are potential applications of detailed lymphocyte subset analysis across a range of autoimmune and inflammatory conditions. These may include assisting diagnosis (40), disease sub-type stratification (41–43) and monitoring of treatment efficacy (44). Immunophenotyping of peripheral blood lymphocytes has revealed distinct immune signatures in many patient cohorts. Compared to healthy controls, previous studies have identified that people with SLE exhibit helper T cell skewing towards Th17 (45), increased proportions of Tregs, cTfh, switched memory B cells (46) and plasmablasts (46, 47). People with RA exhibit increased frequency of cTfh (44, 48, 49), particularly PD-1^+^ cTfh (44, 49), and increased naïve B cells (44). A similar phenotype was identified in people with Sjögrens syndrome (SS), who exhibited increased cTfh (50) and naïve B cells, memory B cells and plasma cells (51). Patients with systemic sclerosis have been found to have reduced Th1/increased Th2 populations (52), and a bias towards effector CD8 and naïve CD4 T cells (53), and reduced γδ T cells (54). People with inclusion body myositis exhibit an increased frequency of CD8 TEMRA (55) and CD8 CD57^+^ cells (56). Flow cytometry immunophenotyping was able to differentiate people with anti-synthetase syndrome from those with dermatomyositis, inclusion body myositis and healthy controls, owing to their increased proportion of Effector Memory CD4 T cells (57). In particular, the cTfh values described by Jin et al. (51) for patients with Sjögrens syndrome fall outside the range of values seen for this population in our study.

Many of these studies used conventional flow cytometry, which limited the number of lymphocyte populations that could be assessed simultaneously. As a result, analyses were often divided between separate “T cell” and “B cell” panels, with little to no overlap in marker sets. This separation restricted the ability to examine cross-lineage marker expression - for example, T cell markers on B cells or vice versa - thereby limiting opportunities for discovery of novel or unexpected immunophenotypic patterns associated with disease. In contrast, our panel enables the simultaneous measurement of all these populations from a single sample, addressing a significant limitation in current methodologies. As these research studies have not yet been translated into clinical practice, our future work will focus on clinical application of this panel to confirm the associations made in the listed papers, with the ultimate goal of developing a diagnostic tool that bridges the translational gap.

## Data Availability

The raw data supporting the conclusions of this article will be made available by the authors, without undue reservation.
