# Supplementary material for: Advanced immunophenotyping of lymphocyte and monocyte subsets in healthy Australian adults using a novel spectral flow cytometry panel
Source: Front Immunol. 2025 Jul 22;16:1577206. doi: 10.3389/fimmu.2025.1577206 (PMC12322900; doi:10.3389/fimmu.2025.1577206)
Supplement: Supplementary file 1 [file DataSheet1.pdf]

Davies et al, Front. Immunol., doi:10.3389/fimmu.2025.1577206

Supplementary Material 1: Supplementary Table 1
Supplementary Figures 1-12

Supplementary Table 1

Table 1 - Initial Panel Design for 31 colour panel. This panel was optimized to produce the final panel described in the main figures.

Laser	Peak channel	Marker	Fluorophore	Supplier	Cat	Clone	Ab host species	Ab Isotype
405nm	V1	CXCR5	BV421	Invitrogen	404-9185-41	MU5UBEE	Mouse	IgG2b κ
	V2	CD16	Super Bright 436	Invitrogen	62-0168-41	eBioCB16	Mouse	IgG1 κ
	V3	Viability	v450	Tonbo	13-0863	N/A		
	V5	CD11c	BV480	BD	566184	B-ly6	Mouse	IgG1 κ
	V5	CD4	cFluor V505	Cytek	R7-20248	SK3	Mouse	IgG1 κ
	V7	IgG	BV510	BD	563247	G18-145	Mouse	IgG1 κ
	V8	CD45	cFluor V547	Cytek	R7-20012	HI30	Mouse	IgG1 κ
	V10	Igκ	BV605	BD	752959	G20-193	Mouse	IgG1 κ
	V11	CCR7	BV650	BioLegend	353233	G043H7	Mouse	IgG2a κ
	V13	CD24	BV711	Biolegend	311135	ML5	Mouse	IgG2a κ
	V14	PD-1	BV750	BioLegend	329965	EH12.2H7	Mouse	IgG1 κ
	V15	CD45RA	BV785	Biolegend	304139	HI100	Mouse	IgG2b κ
488nm	B1	IgM	BB515	BD	564622	G20-127	Mouse	IgG1 κ
	B2	CD57	cFluor B532	Cytek	RC-00127	HNK-1	N/A	N/A
	B3	CD14	cFluor B548	Cytek	R7-20116	63D3	Mouse	IgG1 κ
	B4	CD21	PE	BD	561768	B-ly4	Mouse	IgG1 κ
	B6	CCR6	PE-Dazzle 594	BioLegend	353429	G034E3		
	B7	CD19	PE-Fire 640	BioLegend	302273	HIB19	Mouse	IgG1 κ
	B8	CD8	PerCP	Tonbo	67-0087	SK1	Mouse	IgG1 κ
	B9	Igλ	cFluor B690	Cytek	R7-20260	1-155-2	Mouse	IgG1 κ
	B10	CD25	cFluor BYG710	Cytek	R7-20585	BC-96	Mouse	IgG1 κ
	B12	IgD	cFluor BYG750	Cytek	RC-00521	N/A	N/A	N/A
	B13	CXCR3	PE-Cy7	BioLegend	353719	G025H7	Mouse	IgG1 κ
	B14	CD38	PE-Fire 810	BioLegend	397225	S17015F	Mouse	IgG2a κ
640nm	R1	IgA	APC	Miltenyi	130-113-998	IS11-8E10	Mouse	IgG1 κ
	R2	CD127	AF647	BioLegend	351317	A019D5	Mouse	IgG1 κ
	R3	TCRgd	AF660	BioLegend	331239	B1	Mouse	IgG1 κ
	R4	CD20	redFluor 710	Tonbo	80-0209	2H7	Mouse	IgG2b κ
	R4	CD56	cFluor R720	Cytek	R7-20090	5.1H11	Mouse	IgG1 κ
	R7	CD3	APC-Cy7	Tonbo	25-0038	UCHT1	Mouse	IgG1 κ
	R8	CD27	APC-Fire 810	BioLegend	302863	O323	Mouse	IgG1 κ

Supplementary Figure 1

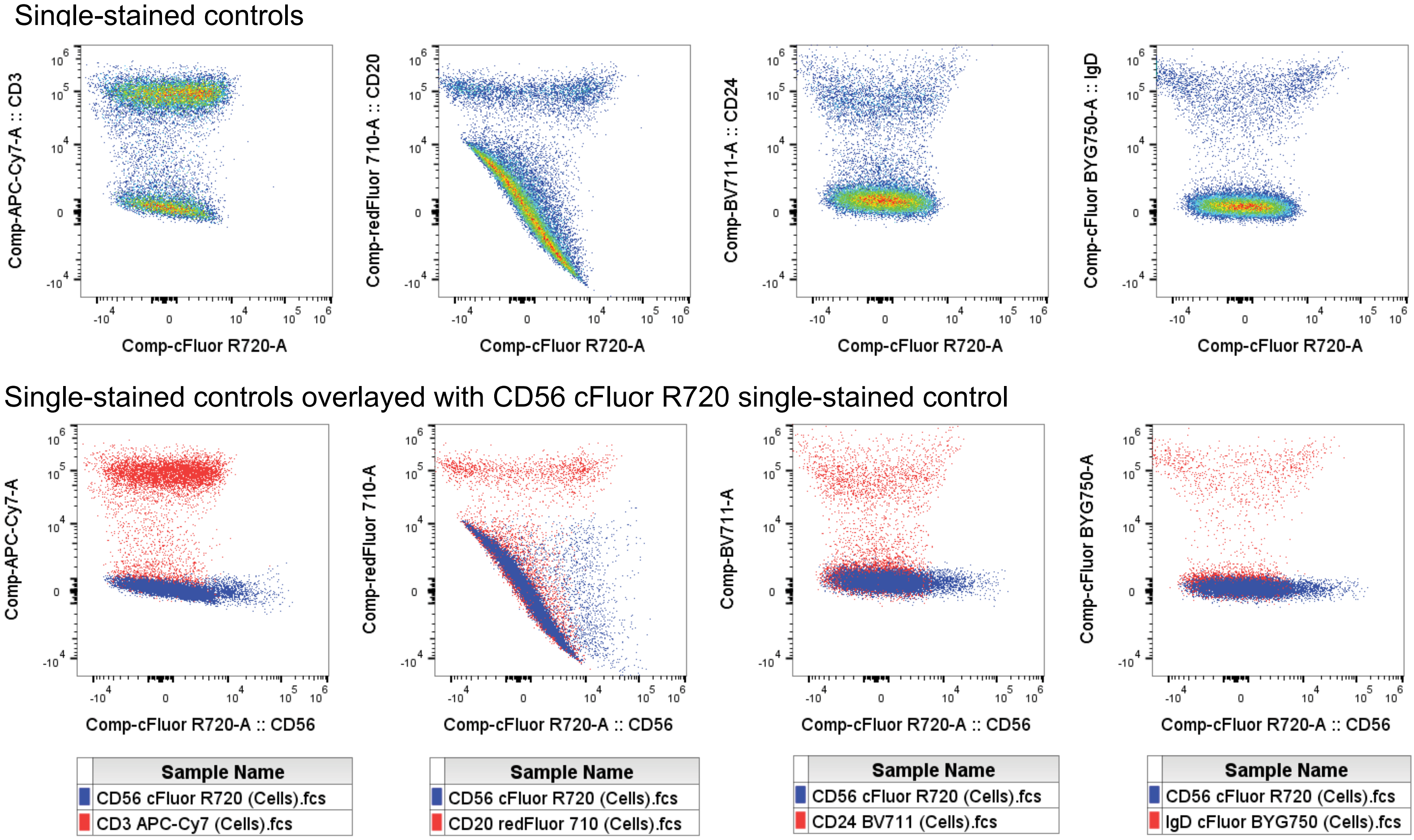

Supplementary Figure 1 - CD56 cFluor R720 staining was negatively impacted by spreading contributed by other fluorophores. From left to right, top row shows PBMCs stained with CD3 APC-Cy7, CD20 redFluor710, CD24 BV711 and IgD cFluor BYG750 only. Bottom row shows these same controls (red) overlayed with CD56 cFluor R720 single-stained control (blue). This demonstrates that the spreading contributed by these colours would make true CD56 positive events difficult to resolve.

Supplementary Figure 2

	BV421	Super Bright 436	cFluor V450	BV480	cFluor V505	BV510	BV570	BV605	BV650	BV711	BV750	BV785	BB515	cFluor B532	cFluor B548	PE	PE-Dazzle 594	PE-Fire 640	PerCP	cFluor B690	cFluor BYG710	cFluor BYG750	PE-Cy7	PE-Fire 810	APC	Alexa Fluor 647	Alexa Fluor 660	APC-Cy7	ViaDye Red	APC-Fire 810
BV421	1																													
Super Bright 436	0.95	1																												
cFluor V450	0.78	0.86	1																											
BV480	0.27	0.34	0.57	1																										
cFluor V505	0.15	0.2	0.34	0.9	1																									
BV510	0.16	0.21	0.35	0.86	0.92	1																								
BV570	0.18	0.18	0.16	0.27	0.25	0.57	1																							
BV605	0.08	0.08	0.06	0.16	0.15	0.41	0.7	1																						
BV650	0.1	0.09	0.08	0.06	0.04	0.16	0.24	0.53	1																					
BV711	0.08	0.08	0.06	0.03	0.01	0.06	0.08	0.18	0.46	1																				
BV750	0.06	0.06	0.04	0.02	0.01	0.04	0.04	0.12	0.25	0.68	1																			
BV785	0.08	0.08	0.06	0.02	0.01	0.03	0.03	0.08	0.16	0.46	0.82	1																		
BB515	0	0	0	0.08	0.03	0.01	0.01	0	0	0	0	0	1																	
cFluor B532	0	0	0	0.06	0.03	0.01	0.02	0	0	0	0	0	0.9	1																
cFluor B548	0.02	0.03	0.04	0.21	0.23	0.29	0.24	0.16	0.05	0.02	0.01	0.01	0.53	0.75	1															
PE	0	0.01	0.01	0.1	0.09	0.21	0.46	0.23	0.05	0.01	0.01	0	0.1	0.17	0.47	1														
PE-Dazzle 594	0	0	0	0.05	0.04	0.12	0.26	0.35	0.15	0.05	0.03	0.02	0.05	0.08	0.31	0.49	1													
PE-Fire 640	0	0	0	0.01	0.01	0.03	0.08	0.15	0.27	0.19	0.08	0.05	0.02	0.02	0.15	0.17	0.56	1												
PerCP	0	0	0	0.01	0	0.04	0.08	0.17	0.4	0.3	0.18	0.09	0	0.01	0.09	0.08	0.33	0.68	1											
cFluor B690	0	0	0	0.01	0	0.04	0.06	0.15	0.36	0.58	0.33	0.22	0	0.01	0.08	0.06	0.29	0.65	0.76	1										
cFluor BYG710	0	0	0	0.01	0	0.02	0.04	0.06	0.15	0.36	0.18	0.11	0.01	0.02	0.09	0.1	0.23	0.54	0.45	0.84	1									
cFluor BYG750	0	0	0	0	0	0.01	0.02	0.04	0.08	0.24	0.3	0.21	0	0.01	0.04	0.03	0.09	0.26	0.24	0.46	0.61	1								
PE-Cy7	0	0	0	0	0	0	0.01	0.02	0.04	0.15	0.22	0.25	0	0	0.03	0.02	0.05	0.16	0.12	0.3	0.36	0.75	1							
PE-Fire 810	0	0	0	0.01	0.01	0.02	0.06	0.03	0.02	0.06	0.09	0.13	0.02	0.03	0.1	0.18	0.12	0.13	0.08	0.18	0.23	0.45	0.74	1						
APC	0	0	0	0	0	0.02	0.02	0.06	0.32	0.21	0.08	0.04	0	0	0.01	0.01	0.02	0.29	0.24	0.26	0.17	0.08	0.03	0.01	1					
Alexa Fluor 647	0	0	0	0	0	0	0	0	0.19	0.19	0.03	0.02	0	0	0	0	0	0.24	0.23	0.29	0.19	0.08	0.03	0.01	0.92	1				
Alexa Fluor 660	0	0	0	0	0	0	0.01	0.01	0.16	0.33	0.08	0.04	0	0	0.01	0.01	0.02	0.23	0.21	0.4	0.33	0.12	0.06	0.02	0.63	0.8	1			
APC-Cy7	0	0	0	0	0	0	0	0.01	0.06	0.21	0.19	0.22	0	0	0	0	0	0.08	0.05	0.16	0.13	0.19	0.18	0.06	0.2	0.22	0.3	1		
ViaDye Red	0	0	0	0	0	0	0	0	0.08	0.29	0.12	0.09	0.01	0	0.01	0	0.01	0.15	0.11	0.29	0.29	0.27	0.18	0.08	0.38	0.45	0.64	0.83	1	
APC-Fire 810	0	0	0	0	0	0	0	0.01	0.05	0.15	0.13	0.2	0	0	0	0	0	0.05	0.04	0.11	0.09	0.11	0.15	0.1	0.16	0.17	0.2	0.68	0.5	1

Complexity™ Index: 19.59

Supplementary Figure 2 - Similarity index of 30 colours in theory. Matrix generated on CYTEK Cloud (cloud.cytekbio.com/). Numbers in each cell indicate the similarity index of the two fluorophores in that row/column pair. Theoretical calculated complexity index is 19.59.

Supplementary Figure 3

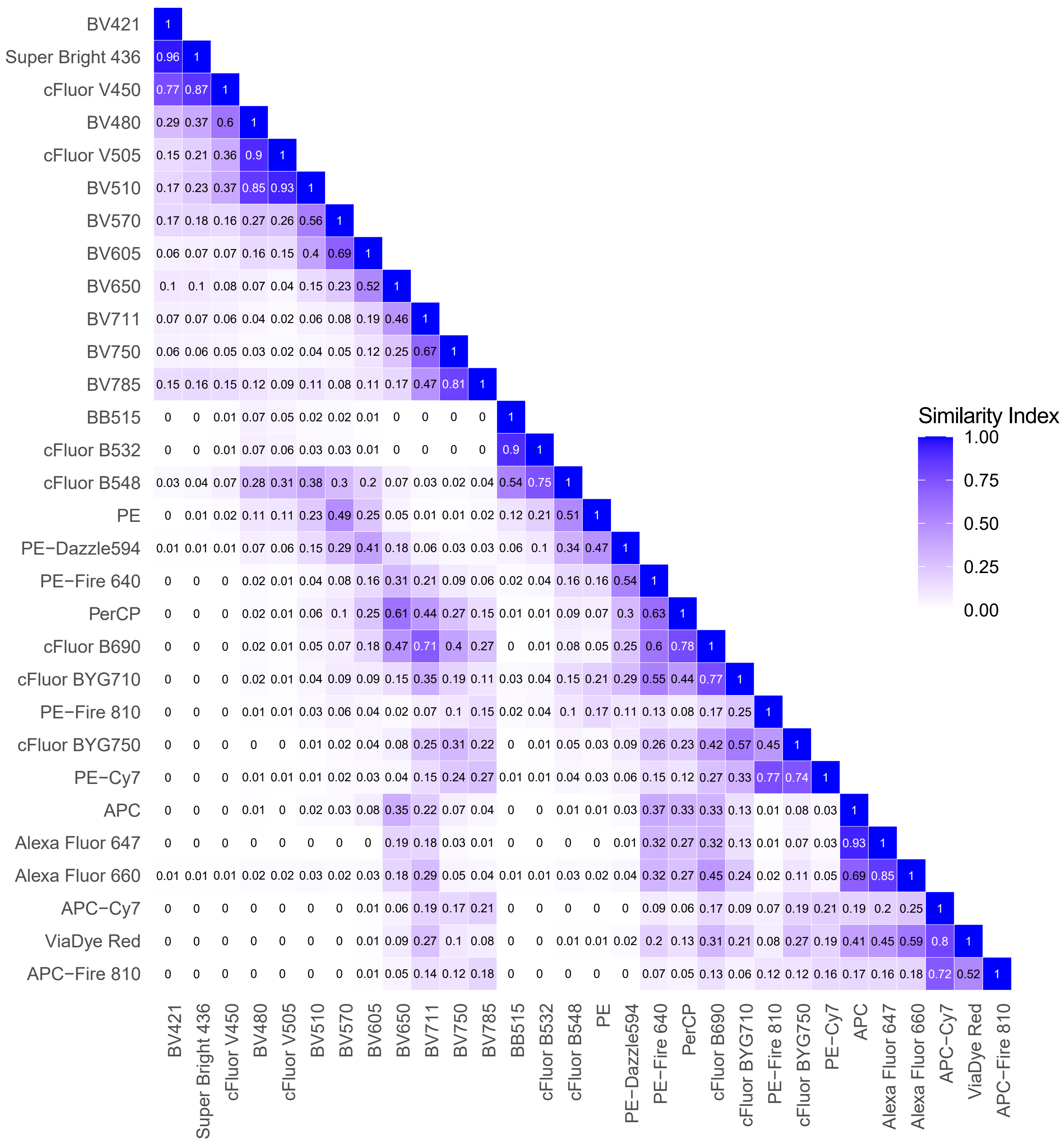

Supplementary Figure 3 - Similarity Index of 30 colours in practice. Matrix generated using ggplot2 in R-studio using similarity indices reported by SpectroFlo after acquiring 30 single-stained controls. The calculated complexity index (SpectroFlo) was 23.25.

Supplementary Figure 4

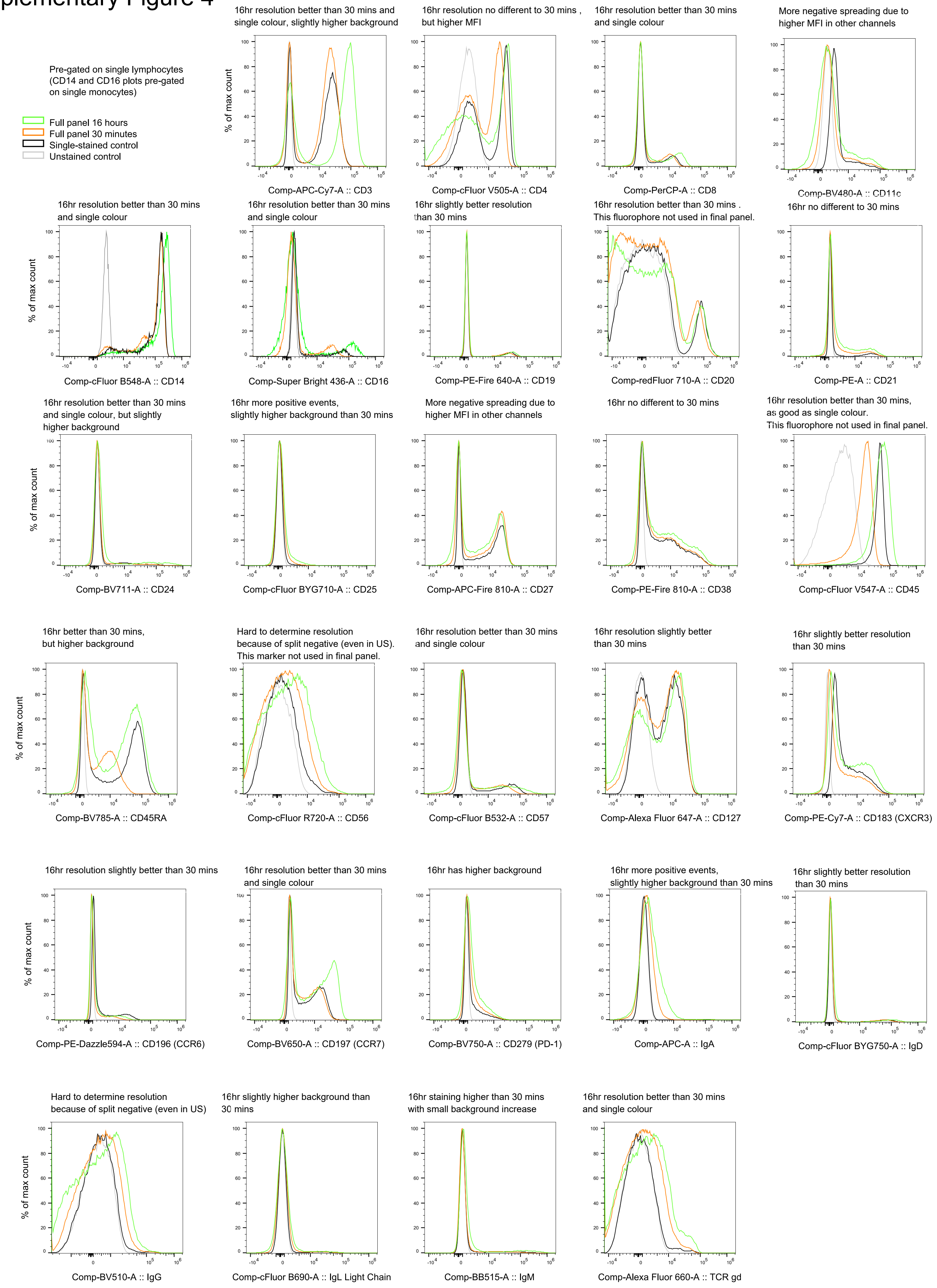

Supplementary Figure 4 - Overnight staining to improve population resolution. PBMCs were stained with antibody cocktail for 30 minutes (orange) or overnight/16 hours (green), stained with one antibody (black), or unstained (grey).

Supplementary Figure 5

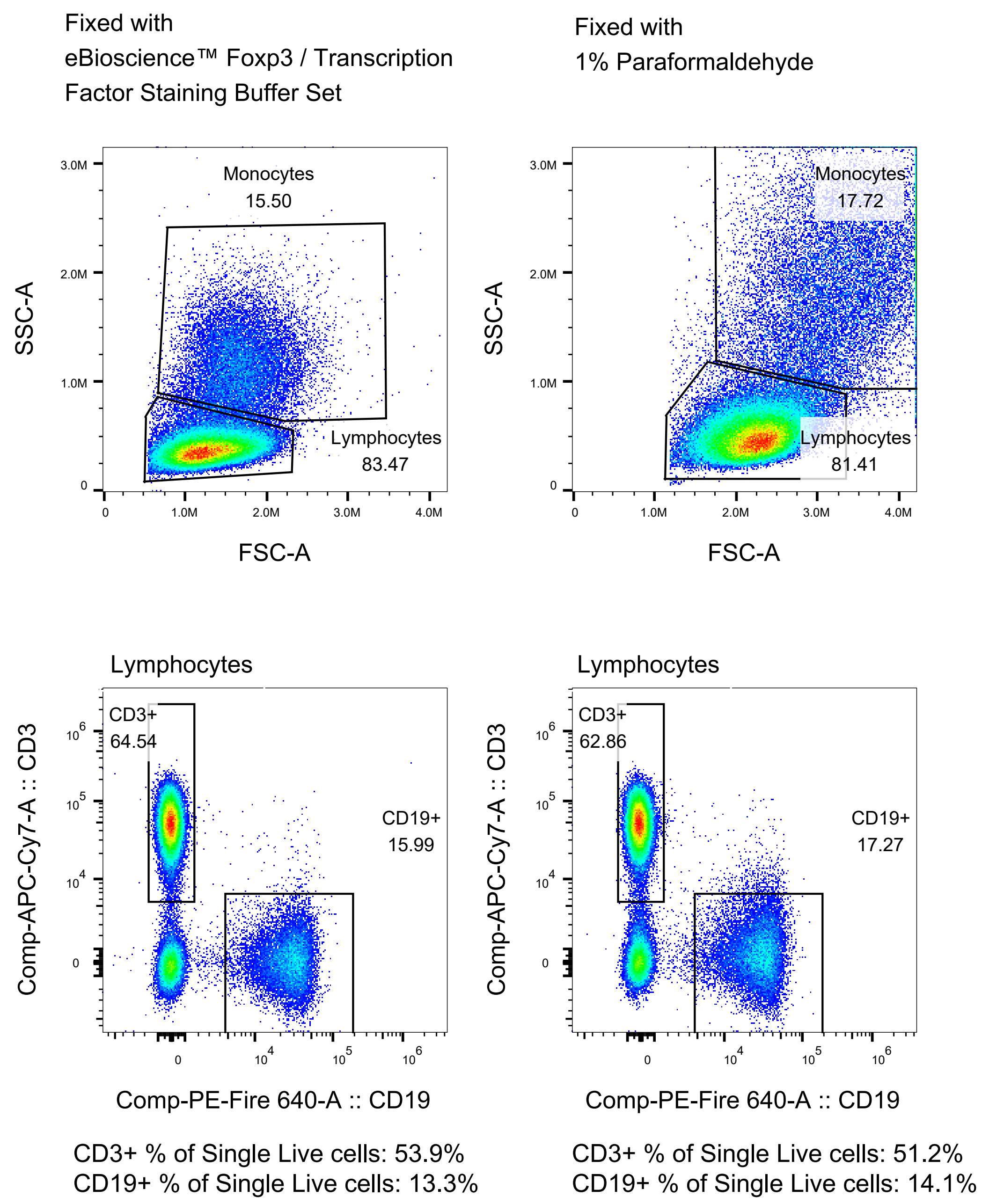

Supplementary Figure 5 - Comparison of two fixation methods. Top row: Single Live cells. Bottom row: Lymphocytes (SSC-A low FSC-A low/mid). Left column: cells fixed with eBioscience ™ Foxp3 / Transcription Factor Staining Buffer Set (ThermoFisher 00-5523-00) as per kit instructions. Right column: cells fixed with 1% paraformaldehyde in 1X PBS (ThermoFisher J19943.K2).

Supplementary Figure 6

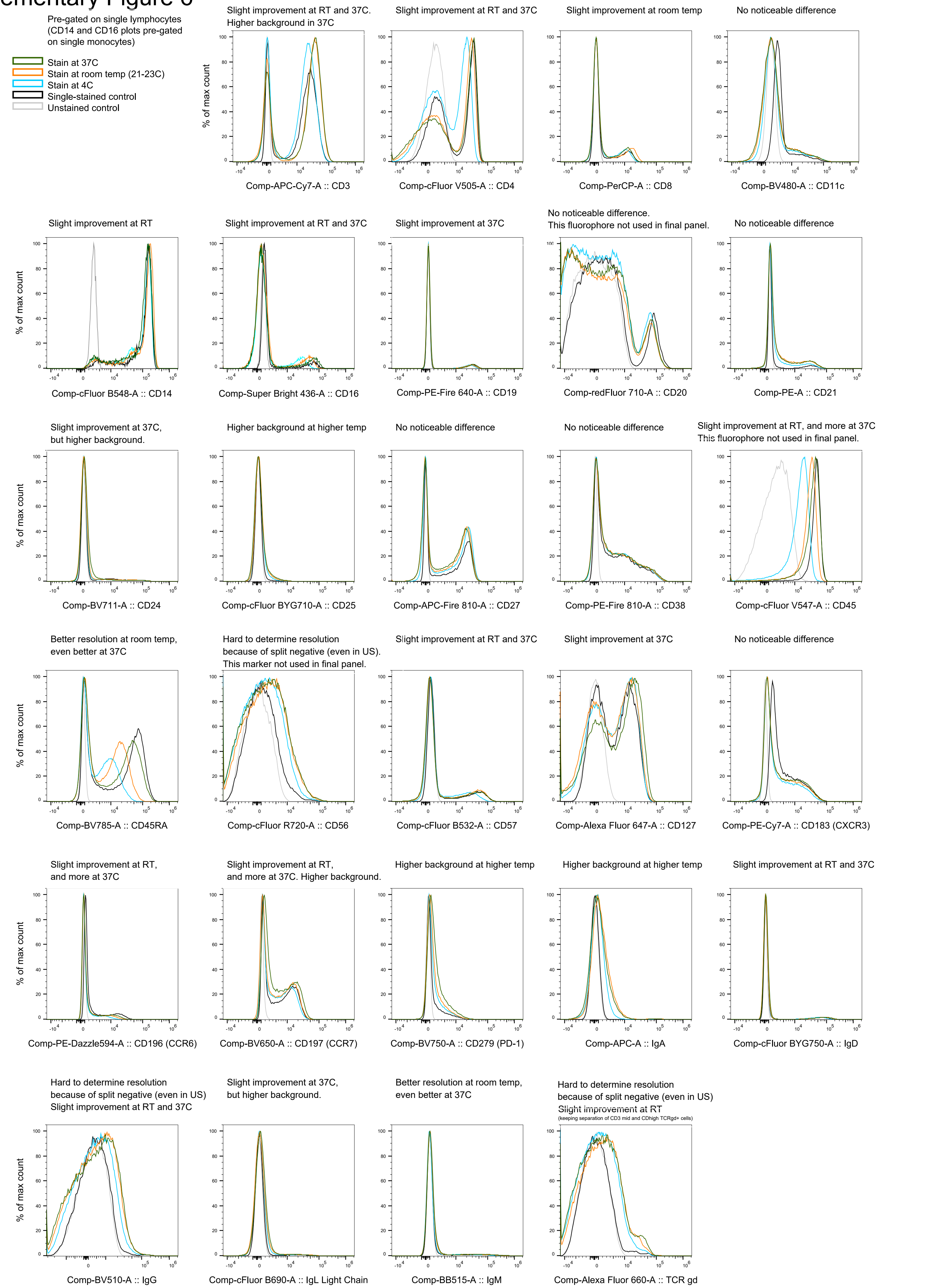

Supplementary Figure 6 - Comparison of staining temperature to improve population resolution. PBMCs were stained with antibody cocktail for 30 minutes. Cells were incubated at 37°C (dark green), room temperature between 21°C and 23°C (orange) or at 4°C (blue). Controls were stained for 30 minutes at 4°C with one antibody (black) or unstained (grey).

Supplementary Figure 7

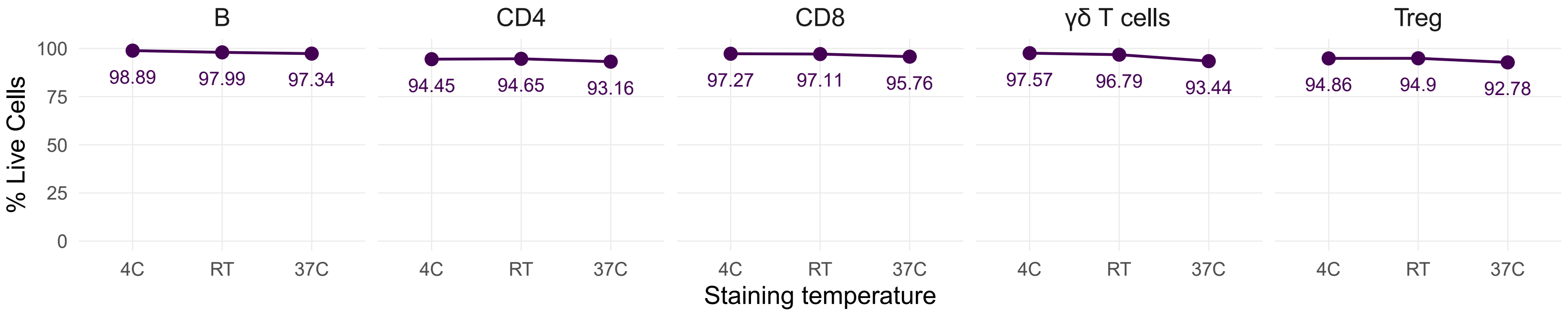

Supplementary Figure 7 - Cell viability after staining at different temperatures. PBMCs were stained with antibody cocktail for 30 minutes at either 4°C, room temperature between 21°C and 23°C, or at 37°C. After staining, cells were incubated with viability dye, followed by fixative. Percentage of live cells were measured among B cells ($CD3^- CD19^+ CD20^+$), CD4 T cells ($CD3^+ TCR\gamma\delta^- CD4^+ CD8^-$), CD8 T cells ($CD3^+ TCR\gamma\delta^- CD4^- CD8^+$), γδ T cells ($CD3^+ TCR\gamma\delta^+$), Tregs ($CD3^+ TCR\gamma\delta^- CD4^+ CD8^- CD127^- CD25^+$).

Supplementary Figure 8

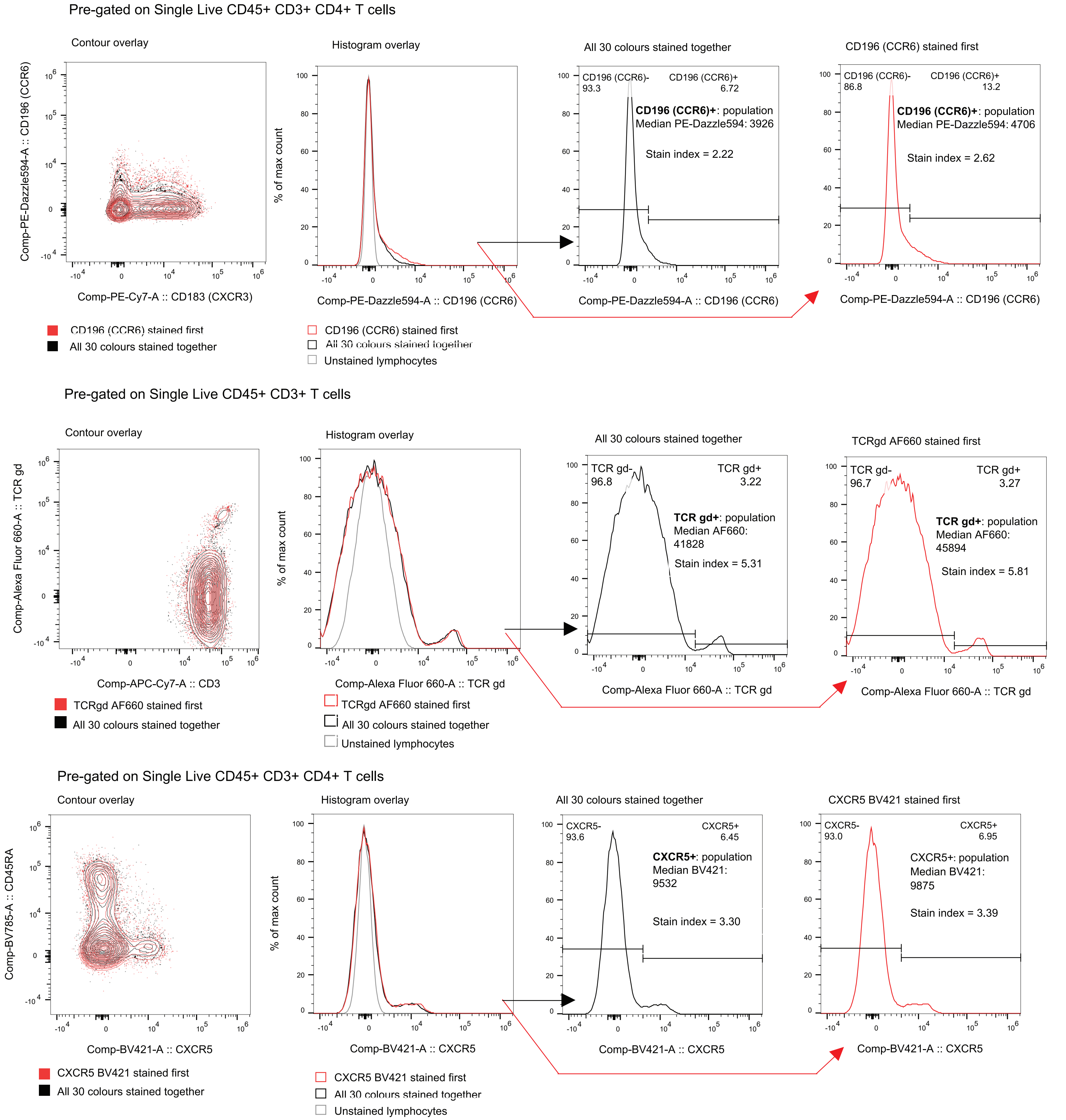

Supplementary Figure 8 - Sequential staining resulted in small improvements in signal for CCR6, TCRγδ and CXCR5. In contour plots or histograms, red lines represent sample where the antibody was stained in a primary layer and all other colours were stained in a secondary layer. Black lines represent sample where all 30 colours stained together in one layer.

The two right histograms show frequency of positive and negative events as percent of parent (e.g. CXCR5- 93.6%, CXCR5+ 6.45%), the MFI of the positive population (e.g. Median BV421 9532) and the Stain index (MFI-positive – MFI-negative / 2 x SD-negative) for that sample (e.g. Stain index = 3.30).

Supplementary Figure 9

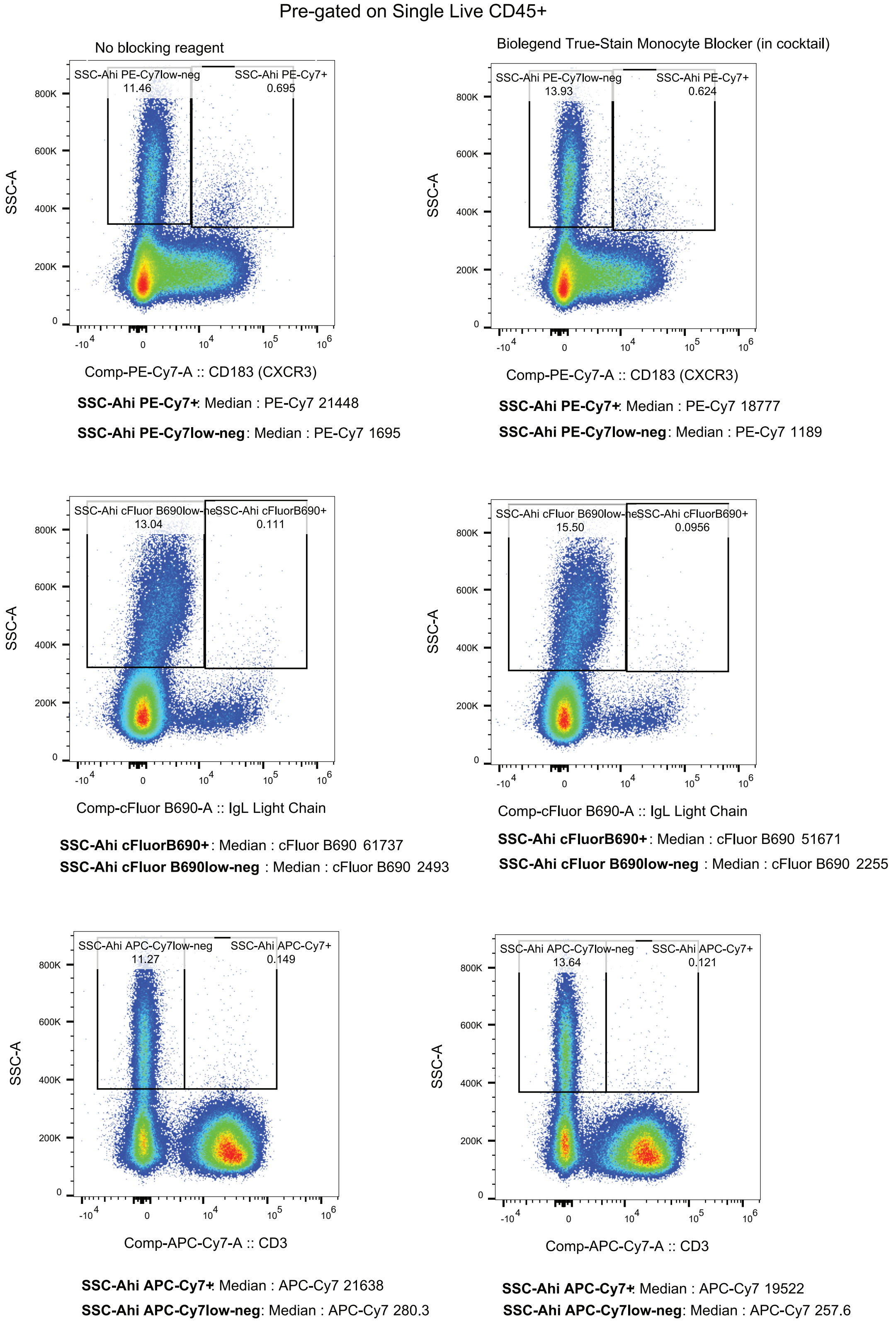

Supplementary Figure 9 - True-Stain Monocyte Blocker (Biolegend) slightly reduces the background staining of cyanine dyes on monocytes. Median MFI of the flurophore for positive and negative SSC-A high cells are shown under each plot.

Supplementary Figure 10

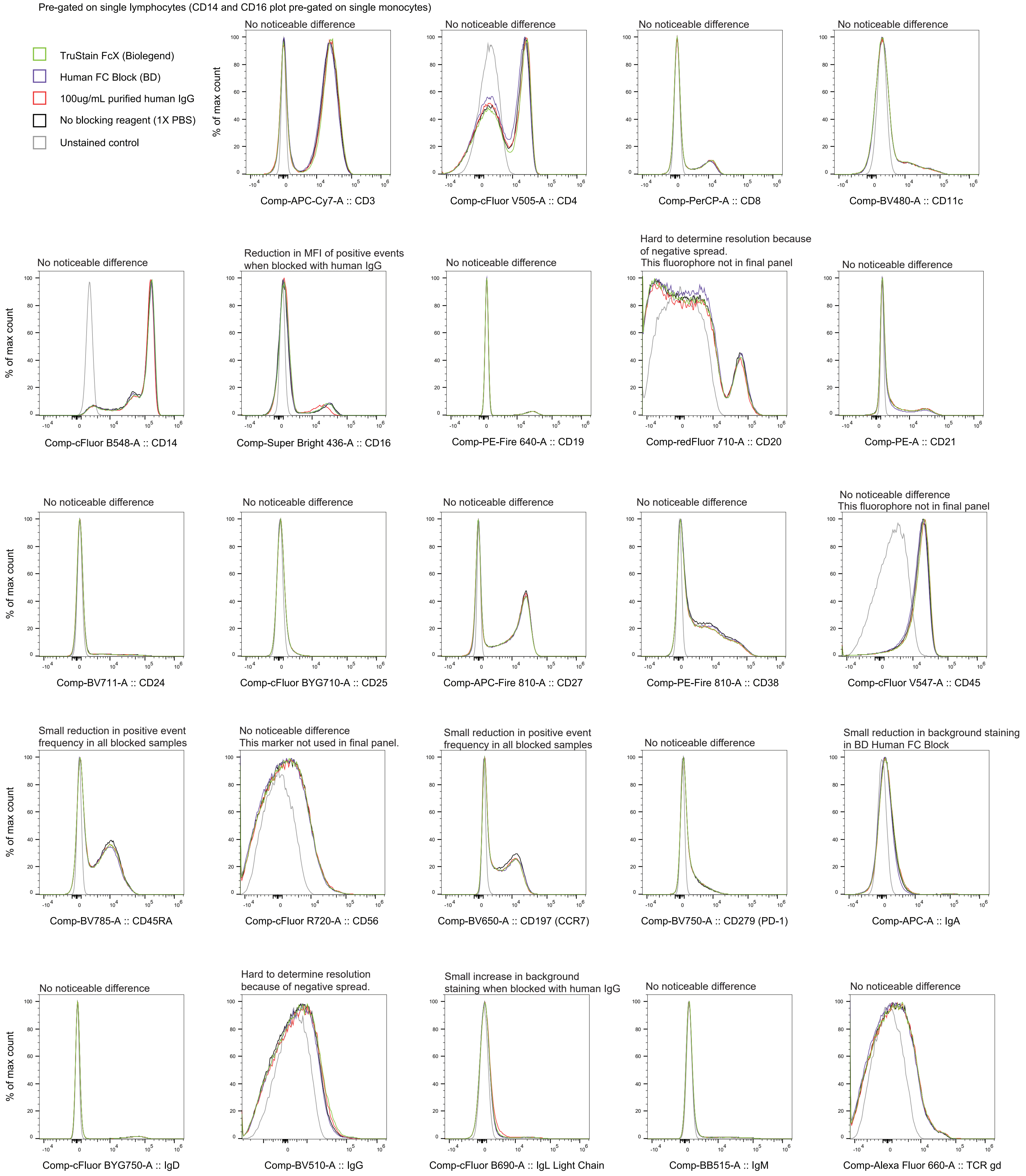

Supplementary Figure 10 - Comparison of Fc blocking to improve population resolution. PBMCs were incubated with either Human TruStain FcX (Biolegend #422301, green line), Human FC Block Pure Fc1 (BD #564220, purple line), purified Human IgG (Merck, #I4506) at 100μg/mL (orange line) or no blocking (only diluent, 1X PBS, black line) before proceeding with panel staining. Unstained control shown in grey.

Supplementary Figure 11

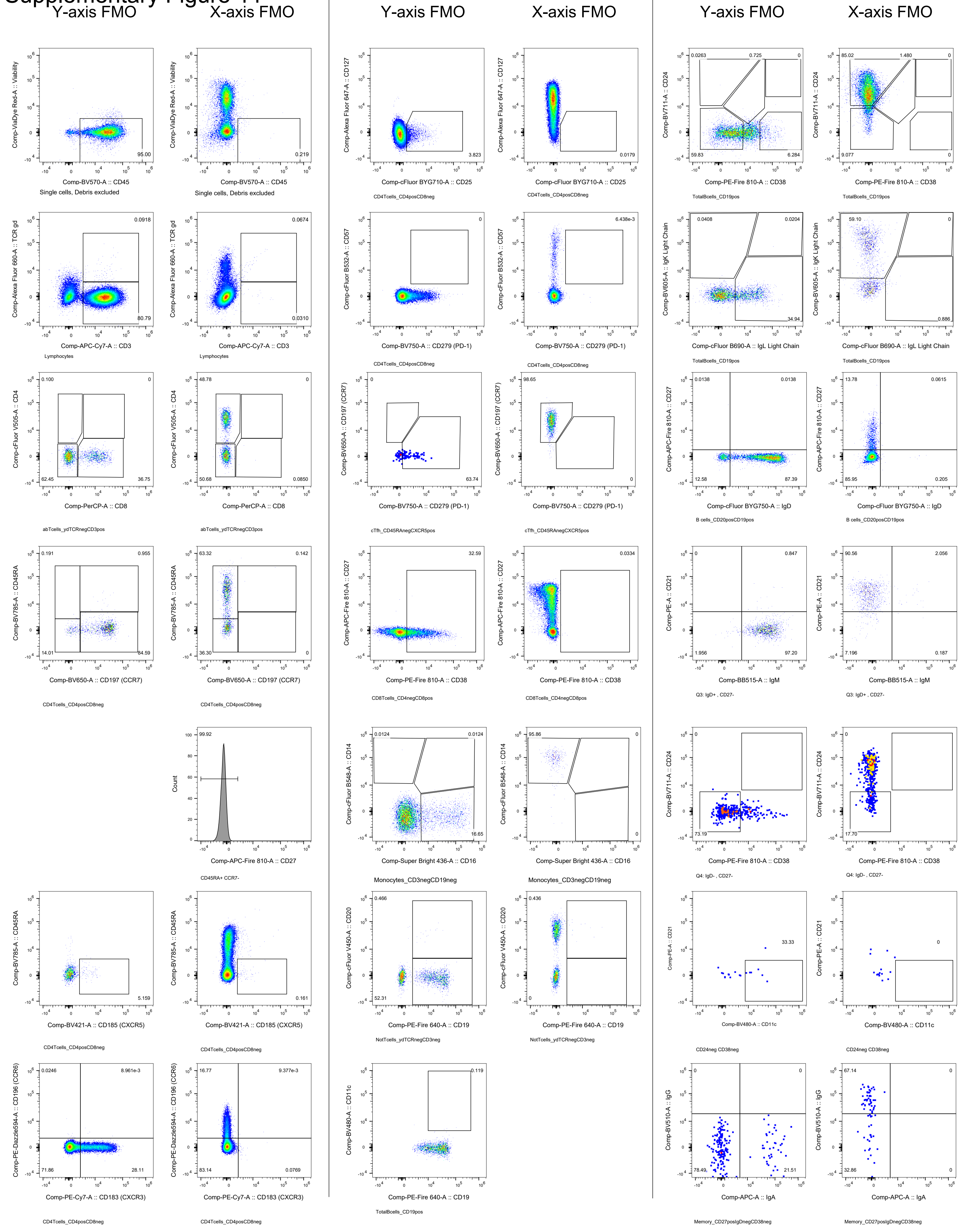

Supplementary Figure 11 - Fluoresence Minus One (FMO) controls. PBMC samples were stained with a cocktail of all antibodies in the panel, except one. Plots show FMO of the Y-axis antibody in the left column and the FMO of the X-axis antibody in the right column. Plots are pre-gated with the relevant population written underneath the plot.

Supplementary Figure 12

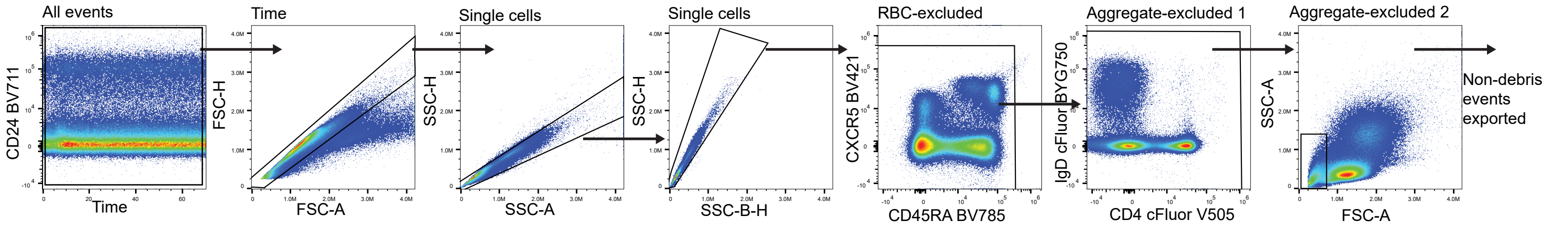

Supplementary Figure 12 - Preliminary gating of FCS files. First, all fluorescent markers vs time are analysed to identify pressure disturbances that may have affected the acquisition of that sample. A time gate is set on CD24 BV711 vs time. A series of doublet exclusion gates are applied (FCS-A vs FSC-H, SSC-A vs SSC-H), followed by a red blood cell exclusion gate (SSC-Blue-H vs SSC-H) which is useful if the PBMC sample had considerable RBC contamination. Two gates are applied to remove any antibody aggregates (hyper-fluorescent events). Finally, small FSC-A events are excluded in a “debris” exclusion gate. This data is saved as a new FCS file which is used for further analysis.
