# Supplementary material for: Advanced immunophenotyping of lymphocyte and monocyte subsets in healthy Australian adults using a novel spectral flow cytometry panel
Source: Front Immunol. 2025 Jul 22;16:1577206. doi: 10.3389/fimmu.2025.1577206 (PMC12322900; doi:10.3389/fimmu.2025.1577206)

Davies et al, Front. Immunol., doi:10.3389/fimmu.2025.1577206

Supplementary Material 2: Supplementary Table 2
Supplementary Figure 13

Supplementary Table 2

Average CV per population of six repeated PBMC samples run in five or more batches

Population	Measurement	n	CV (%)
αβ T cells	% of Lymphocytes	6	4.5
Atypical B cells	% of Total B cells	6	25
B cells Plasmablasts and Plasma Cells	% of Lymphocytes	6	9
CCR6- CXCR3-	% of CD4 T cells	6	6.7
CD4 CD38+ (activated)	% of CD4 T cells	6	14
CD4 Central Memory	% of CD4 T cells	6	10
CD4 Early Effector Memory	% of CD4 Effector Memory	6	9.6
CD4 Early-like/Terminal Effector Memory	% of CD4 Effector Memory	6	16
CD4 Effector Memory	% of CD4 T cells	6	20
CD4 Exhausted	% of CD4 T cells	6	21
CD4 Naive	% of CD4 T cells	6	10
CD4 T cells	% of Lymphocytes	6	5.3
CD4 T cells	% of αβ T cells	6	2.8
CD4 TEMRA	% of CD4 T cells	6	35
CD8 CD38+ (activated)	% of CD8 T cells	6	29
CD8 Central Memory	% of CD8 T cells	6	24
CD8 Early Effector Memory	% of CD8 Effector Memory	6	16
CD8 Early-like/Terminal Effector Memory	% of CD8 Effector Memory	6	30
CD8 Effector Memory	% of CD8 T cells	6	17
CD8 Exhausted	% of CD8 T cells	6	25
CD8 Naive	% of CD8 T cells	6	7.5
CD8 T cells	% of Lymphocytes	6	6.8
CD8 T cells	% of αβ T cells	6	4.8
CD8 TEMRA	% of CD8 T cells	6	25
Classical Monocytes	% of Monocytes	6	5
Double Negative 2	% of B cells	6	14
Double Negative Memory	% of Memory B cells	6	25
γδ CD4	% of γδ T cells	6	31
γδ CD8	% of γδ T cells	6	18
γδ Central Memory	% of γδ T cells	6	14
γδ Effector	% of γδ T cells	6	36
γδ Effector Memory	% of γδ T cells	6	31
γδ Naive	% of γδ T cells	6	32
γδ T cells	% of Lymphocytes	6	9.8
Ig-κ light chain	% of Total B cells	6	2.9
Ig-λ light chain	% of Total B cells	6	3.2
IgA Memory	% of Memory B cells	6	7.2
IgG Memory	% of Memory B cells	6	12
Intermediate Monocytes	% of Monocytes	6	39
Memory B cells	% of B cells	6	16
Monocytes	% of Live CD45+	6	32
Naive Resting	% of B cells	6	5.9
Non-Classical Monocytes	% of Monocytes	6	46
Plasmablasts and Plasma Cells	% of Lymphocytes	6	15
T2 and T3	% of Transitional B cells	6	3.8
Th1	% of CD4 T cells	6	17
Th1/Th17	% of CD4 T cells	6	38
Th17	% of CD4 T cells	6	31
Total T cells	% of Lymphocytes	6	4.3
Transitional	% of B cells	6	15
Treg	% of CD4 T cells	6	22
Unswitched Memory	% of B cells	6	22
cTfh	% of CD4 T cells	6	14
cTfh CCR6- CXCR3-	% of cTfh	6	16
cTfh Central Memory	% of cTfh	6	10
cTfh Effector Memory	% of cTfh	6	23
cTfh Th1	% of cTfh	6	9
cTfh Th1/Th17	% of cTfh	6	40
cTfh Th17	% of cTfh	6	39
cTfr	% of Treg	6	27

Supplementary Figure 13 - Frequency of leukocyte populations measured for six healthy controls over five or more batch repeats. Each coloured line represents one individual, with the cells coming from the same batch of PBMCs frozen in late 2023. Where a point is not shown either that individual's sample was not included in the run, or the quality requirements for inclusion were not met.

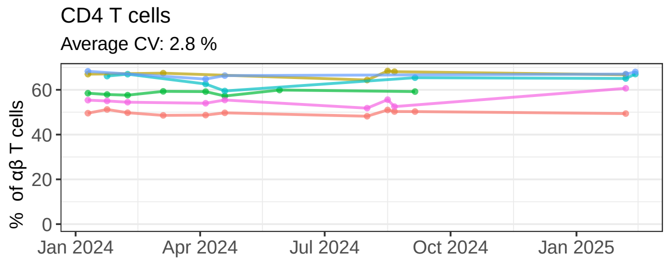
CD4 T cells
Average CV: 2.8 %
% of αβ T cells
0
20
40
60
Jan 2024
Apr 2024
Jul 2024
Oct 2024
Jan 2025

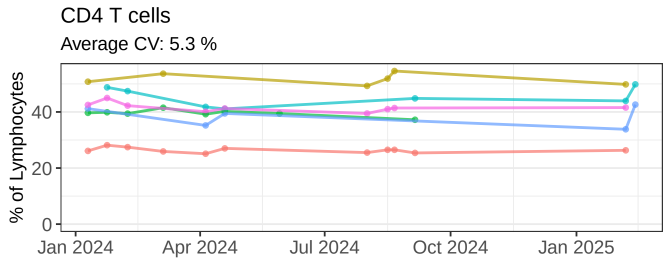
CD4 T cells
Average CV: 5.3 %
% of Lymphocytes
0
20
40
Jan 2024
Apr 2024
Jul 2024
Oct 2024
Jan 2025

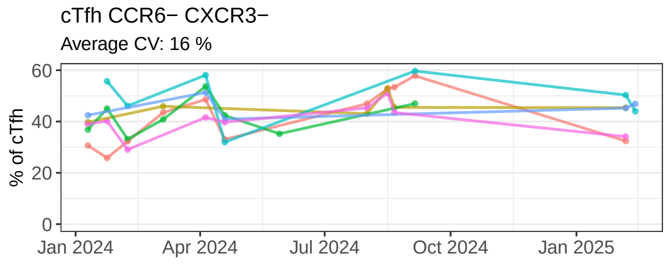
cTfh CCR6− CXCR3−
Average CV: 16 %
% of cTfh
0
20
40
60
Jan 2024
Apr 2024
Jul 2024
Oct 2024
Jan 2025

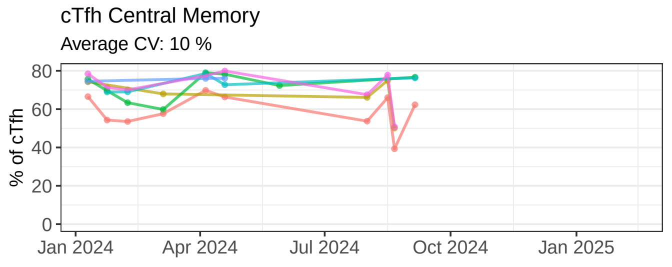
cTfh Central Memory
Average CV: 10 %
% of cTfh
80
60
40
20
0
Jan 2024
Apr 2024
Jul 2024
Oct 2024
Jan 2025

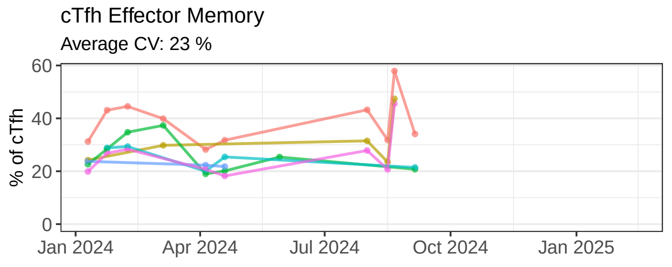

cTfh Effector Memory
Average CV: 23 %
% of cTfh
60
40
20
0
Jan 2024
Apr 2024
Jul 2024
Oct 2024
Jan 2025

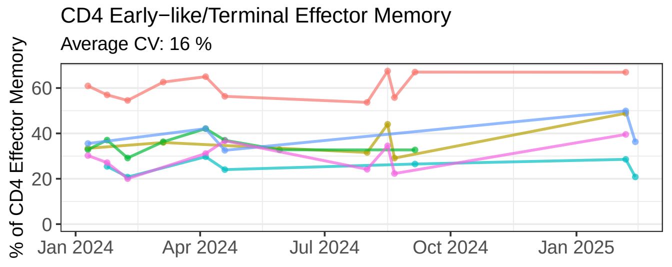

CD4 Early−like/Terminal Effector Memory
Average CV: 16 %
% of CD4 Effector Memory
60
40
20
0
Jan 2024
Apr 2024
Jul 2024
Oct 2024
Jan 2025

Th1

Average CV: 17 %

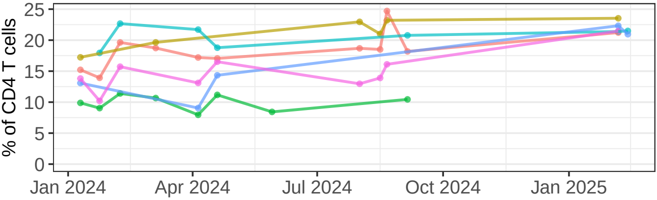

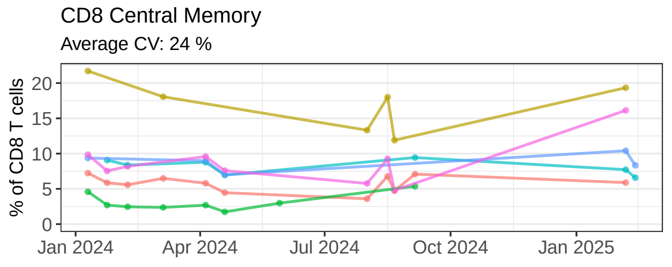
CD8 Central Memory
Average CV: 24 %
% of CD8 T cells
0
5
10
15
20
Jan 2024
Apr 2024
Jul 2024
Oct 2024
Jan 2025

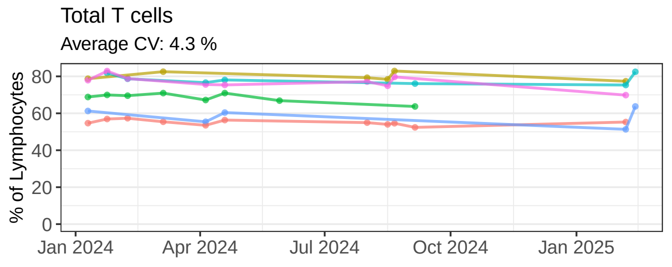
Total T cells
Average CV: 4.3 %
% of Lymphocytes
0
20
40
60
80
Jan 2024
Apr 2024
Jul 2024
Oct 2024
Jan 2025

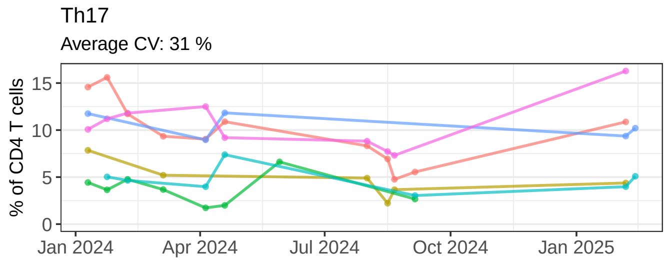
Th17
Average CV: 31 %
% of CD4 T cells
0
5
10
15
Jan 2024
Apr 2024
Jul 2024
Oct 2024
Jan 2025

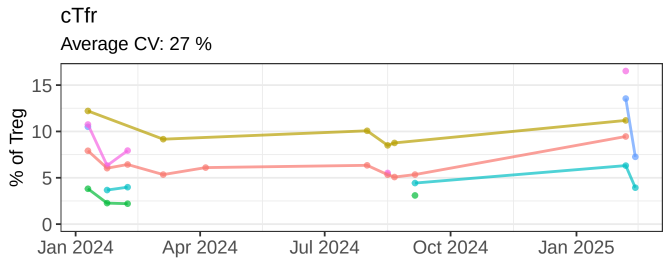

cTfr
Average CV: 27 %
% of Treg
15
10
5
0
Jan 2024
Apr 2024
Jul 2024
Oct 2024
Jan 2025

CD8 T cells

Average CV: 6.8 %

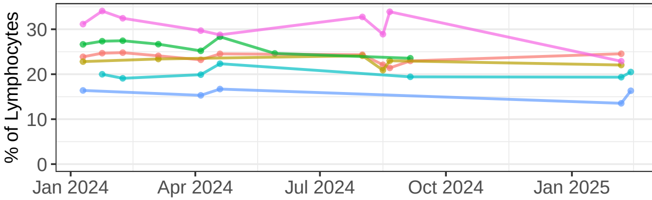

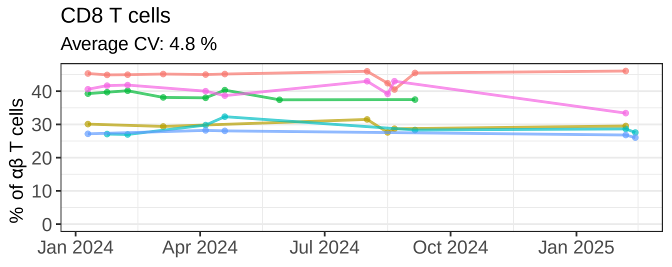

CD8 T cells
Average CV: 4.8 %
% of αβ T cells
0
10
20
30
40
Jan 2024
Apr 2024
Jul 2024
Oct 2024
Jan 2025

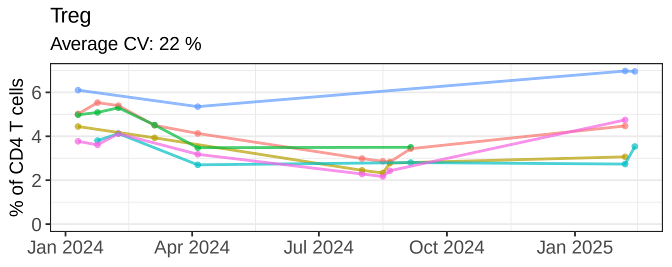

Treg
Average CV: 22 %
% of CD4 T cells
6
4
2
0
Jan 2024
Apr 2024
Jul 2024
Oct 2024
Jan 2025

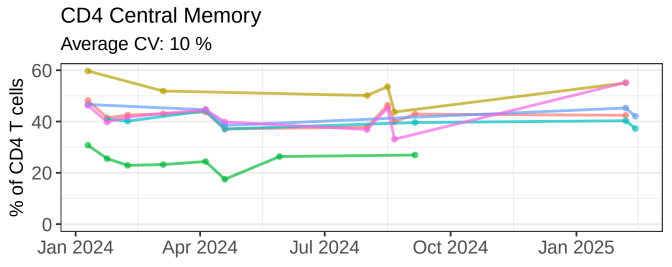
CD4 Central Memory
Average CV: 10 %
% of CD4 T cells
60
40
20
0
Jan 2024
Apr 2024
Jul 2024
Oct 2024
Jan 2025

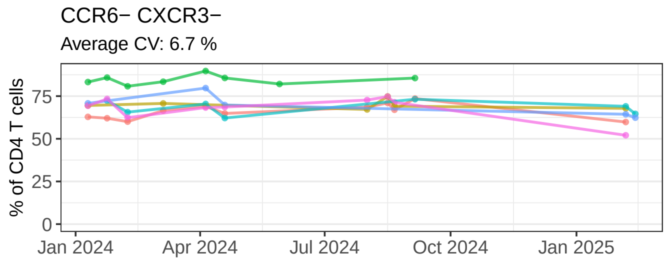
CCR6− CXCR3−
Average CV: 6.7 %
% of CD4 T cells
0
25
50
75
Jan 2024
Apr 2024
Jul 2024
Oct 2024
Jan 2025

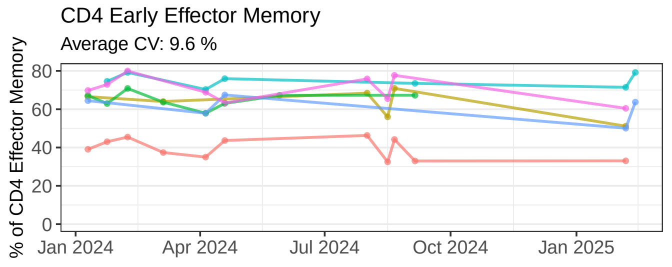
CD4 Early Effector Memory
Average CV: 9.6 %
% of CD4 Effector Memory
80
60
40
20
0
Jan 2024
Apr 2024
Jul 2024
Oct 2024
Jan 2025

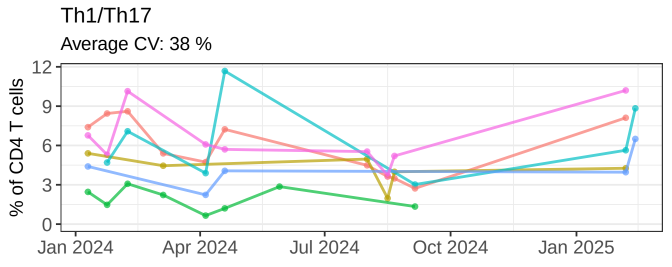
Th1/Th17
Average CV: 38 %
% of CD4 T cells
12
9
6
3
0
Jan 2024
Apr 2024
Jul 2024
Oct 2024
Jan 2025

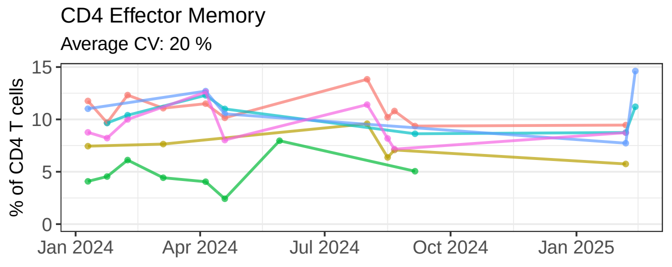
CD4 Effector Memory
Average CV: 20 %
% of CD4 T cells
15
10
5
0
Jan 2024
Apr 2024
Jul 2024
Oct 2024
Jan 2025

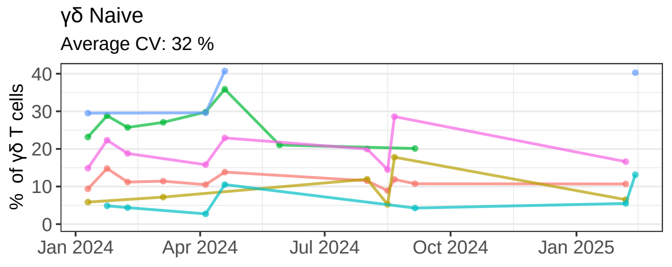

γδ Naive
Average CV: 32 %
% of γδ T cells
40
30
20
10
0
Jan 2024
Apr 2024
Jul 2024
Oct 2024
Jan 2025

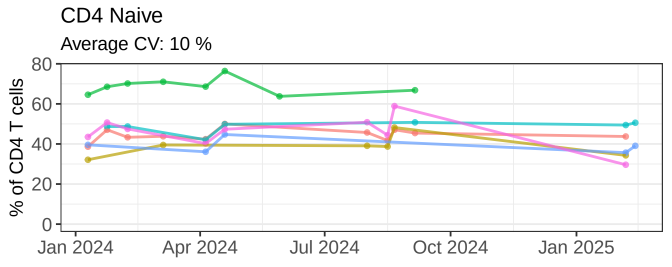
CD4 Naive
Average CV: 10 %
% of CD4 T cells
80
60
40
20
0
Jan 2024
Apr 2024
Jul 2024
Oct 2024
Jan 2025

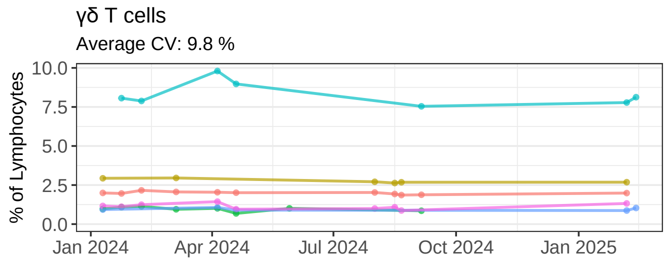
γδ T cells
Average CV: 9.8 %
% of Lymphocytes
10.0
7.5
5.0
2.5
0.0
Jan 2024
Apr 2024
Jul 2024
Oct 2024
Jan 2025

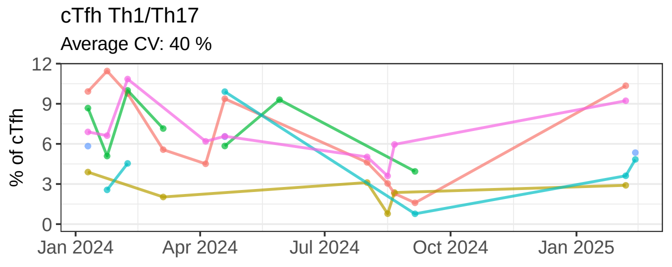
cTfh Th1/Th17
Average CV: 40 %
% of cTfh
12
9
6
3
0
Jan 2024
Apr 2024
Jul 2024
Oct 2024
Jan 2025

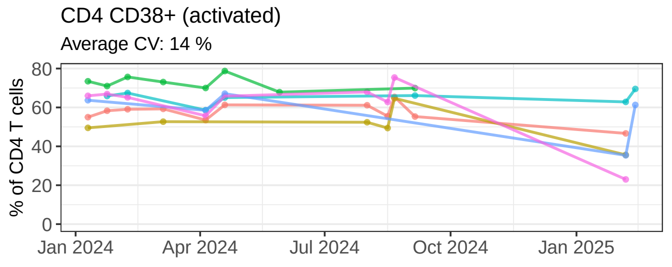

CD4 CD38+ (activated)
Average CV: 14 %
% of CD4 T cells
80
60
40
20
0
Jan 2024
Apr 2024
Jul 2024
Oct 2024
Jan 2025

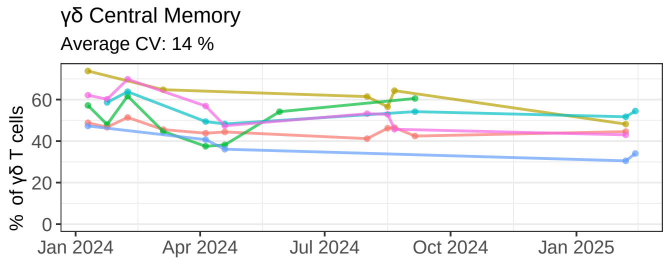
γδ Central Memory
Average CV: 14 %
% of γδ T cells
60
40
20
0
Jan 2024
Apr 2024
Jul 2024
Oct 2024
Jan 2025

γδ Effector Memory

Average CV: 31 %

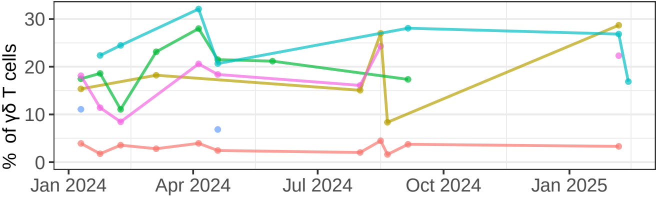

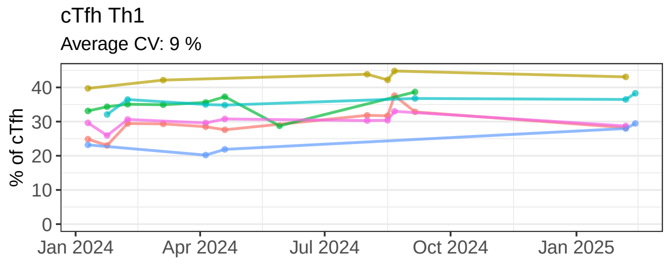
cTfh Th1
Average CV: 9 %
% of cTfh
40
30
20
10
0
Jan 2024
Apr 2024
Jul 2024
Oct 2024
Jan 2025

CD8 Early−like/Terminal Effector Memory

Average CV: 30 %

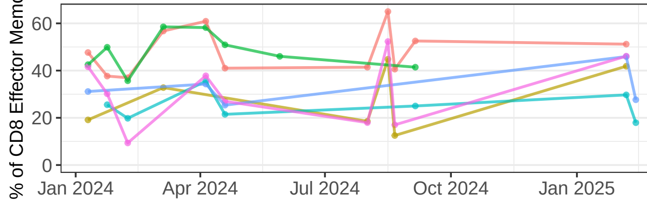

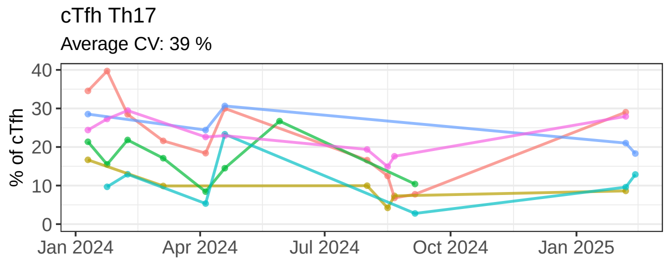

cTfh Th17
Average CV: 39 %
% of cTfh
40
30
20
10
0
Jan 2024
Apr 2024
Jul 2024
Oct 2024
Jan 2025

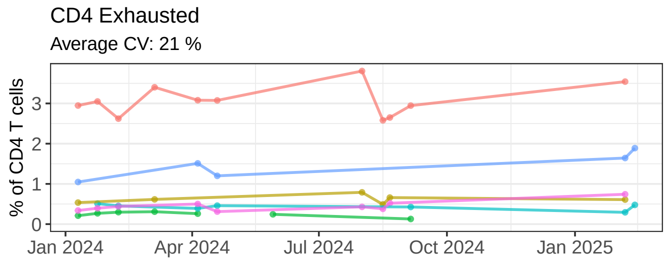

CD4 Exhausted
Average CV: 21 %
% of CD4 T cells
0
1
2
3
Jan 2024
Apr 2024
Jul 2024
Oct 2024
Jan 2025

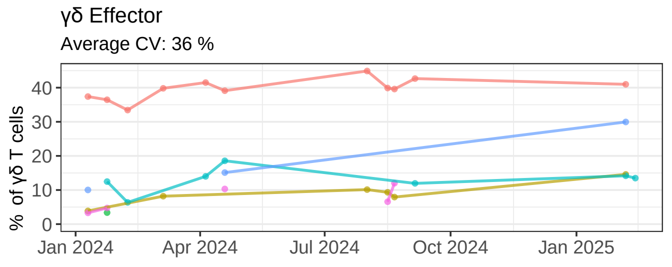
γδ Effector
Average CV: 36 %
% of γδ T cells
0
10
20
30
40
Jan 2024
Apr 2024
Jul 2024
Oct 2024
Jan 2025

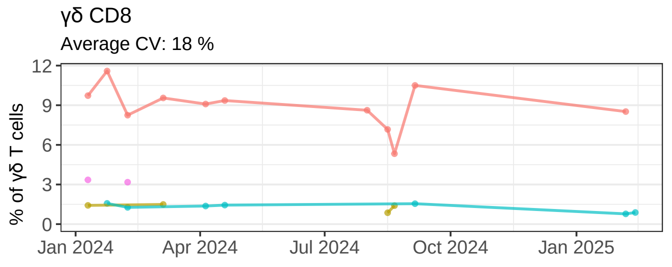
γδ CD8
Average CV: 18 %
% of γδ T cells
12
9
6
3
0
Jan 2024
Apr 2024
Jul 2024
Oct 2024
Jan 2025

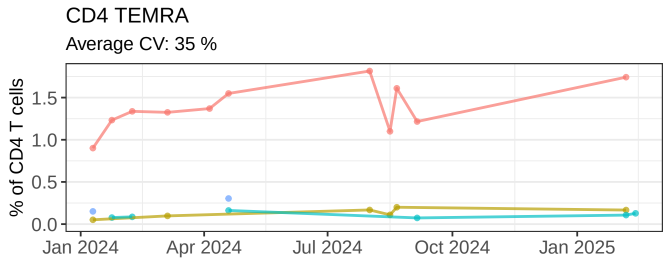

CD4 TEMRA
Average CV: 35 %
% of CD4 T cells
1.5
1.0
0.5
0.0
Jan 2024
Apr 2024
Jul 2024
Oct 2024
Jan 2025

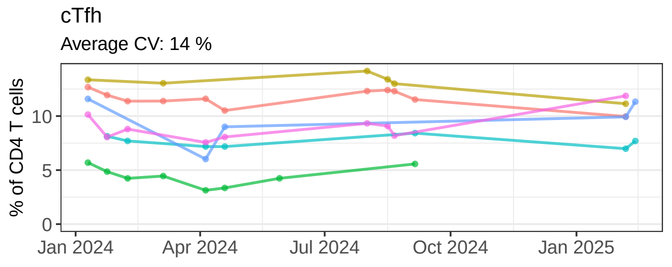
cTfh
Average CV: 14 %
% of CD4 T cells
0
5
10
Jan 2024
Apr 2024
Jul 2024
Oct 2024
Jan 2025

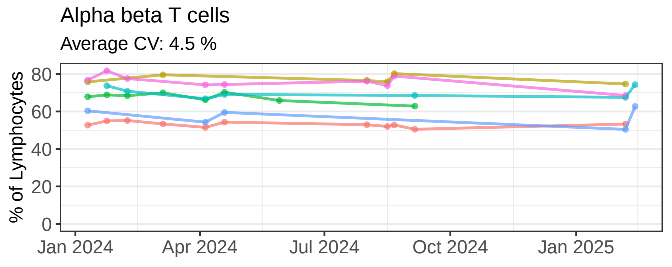
Alpha beta T cells
Average CV: 4.5 %
% of Lymphocytes
80
60
40
20
0
Jan 2024
Apr 2024
Jul 2024
Oct 2024
Jan 2025

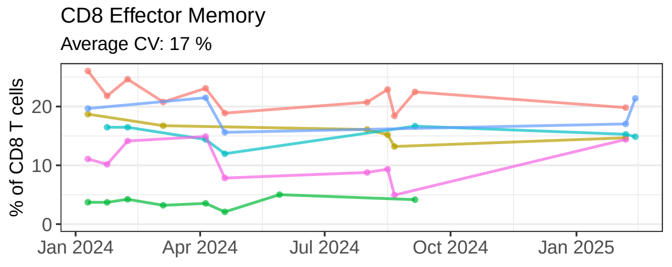

CD8 Effector Memory
Average CV: 17 %
% of CD8 T cells
0
10
20
Jan 2024
Apr 2024
Jul 2024
Oct 2024
Jan 2025

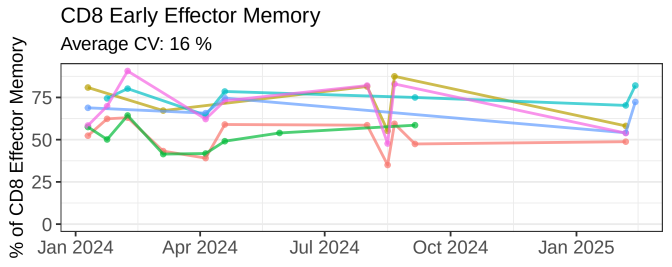

CD8 Early Effector Memory
Average CV: 16 %
% of CD8 Effector Memory
0
25
50
75
Jan 2024
Apr 2024
Jul 2024
Oct 2024
Jan 2025

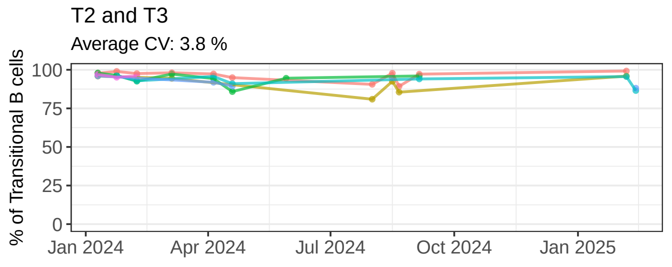
T2 and T3
Average CV: 3.8 %
% of Transitional B cells
100
75
50
25
0
Jan 2024
Apr 2024
Jul 2024
Oct 2024
Jan 2025

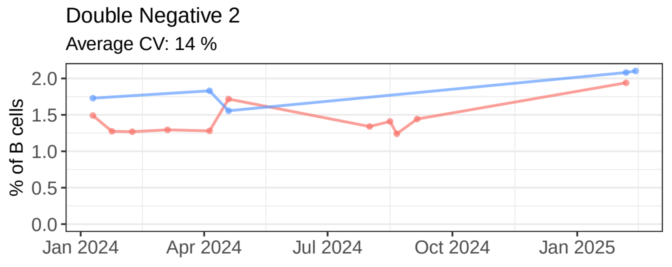
Double Negative 2
Average CV: 14 %
% of B cells
2.0
1.5
1.0
0.5
0.0
Jan 2024
Apr 2024
Jul 2024
Oct 2024
Jan 2025

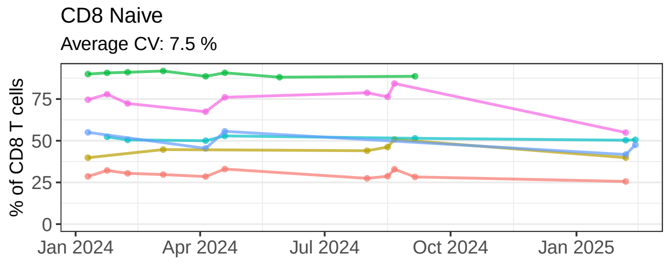
CD8 Naive
Average CV: 7.5 %
% of CD8 T cells
0
25
50
75
Jan 2024
Apr 2024
Jul 2024
Oct 2024
Jan 2025

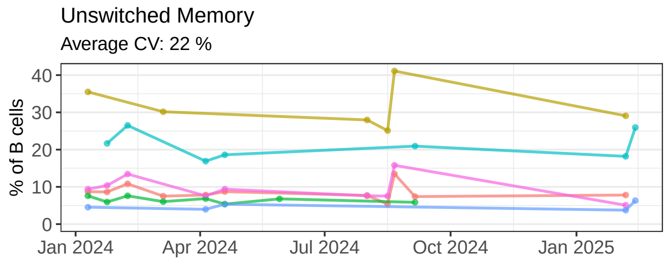
Unswitched Memory
Average CV: 22 %
% of B cells
0
10
20
30
40
Jan 2024
Apr 2024
Jul 2024
Oct 2024
Jan 2025

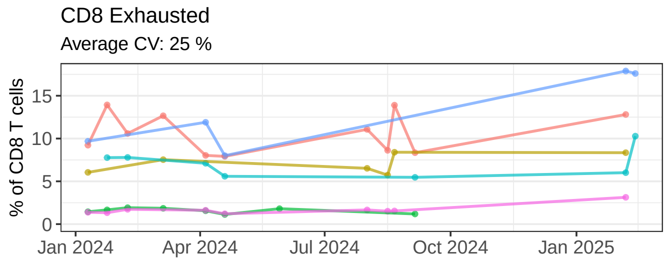
CD8 Exhausted
Average CV: 25 %
% of CD8 T cells
0
5
10
15
Jan 2024
Apr 2024
Jul 2024
Oct 2024
Jan 2025

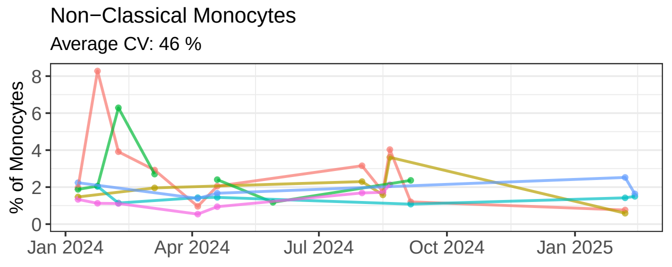

Non−Classical Monocytes
Average CV: 46 %
% of Monocytes
0
2
4
6
8
Jan 2024
Apr 2024
Jul 2024
Oct 2024
Jan 2025

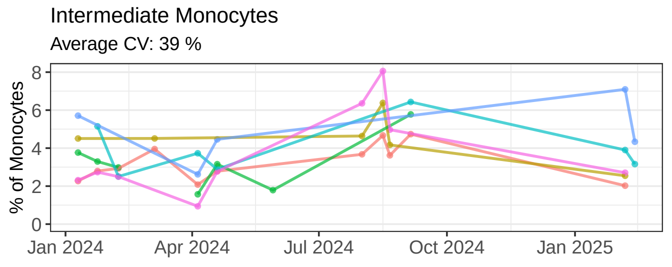

Intermediate Monocytes
Average CV: 39 %
% of Monocytes
0
2
4
6
8
Jan 2024
Apr 2024
Jul 2024
Oct 2024
Jan 2025

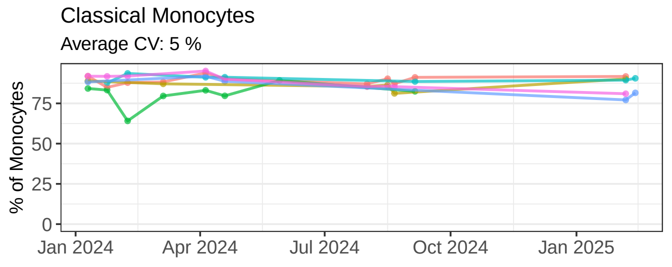

Classical Monocytes
Average CV: 5 %
% of Monocytes
0
25
50
75
Jan 2024
Apr 2024
Jul 2024
Oct 2024
Jan 2025

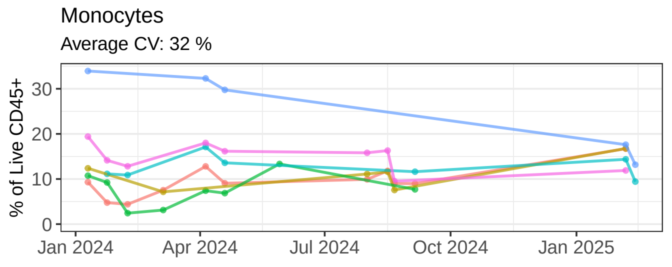
Monocytes
Average CV: 32 %
% of Live CD45+
30
20
10
0
Jan 2024
Apr 2024
Jul 2024
Oct 2024
Jan 2025

CD8 TEMRA

Average CV: 25 %

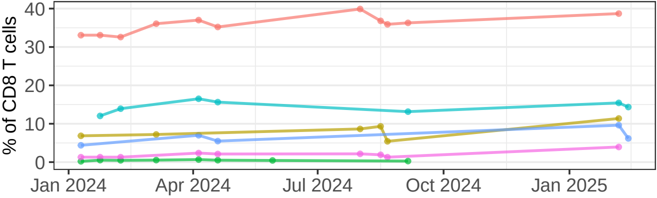

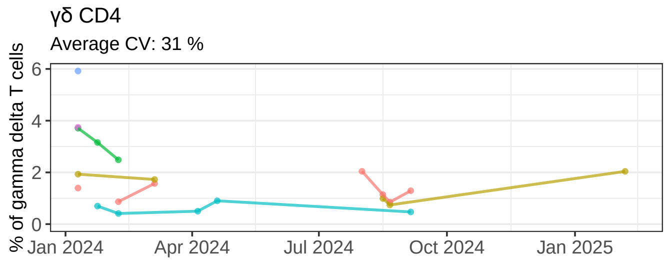

γδ CD4
Average CV: 31 %
% of gamma delta T cells
0
2
4
6
Jan 2024
Apr 2024
Jul 2024
Oct 2024
Jan 2025

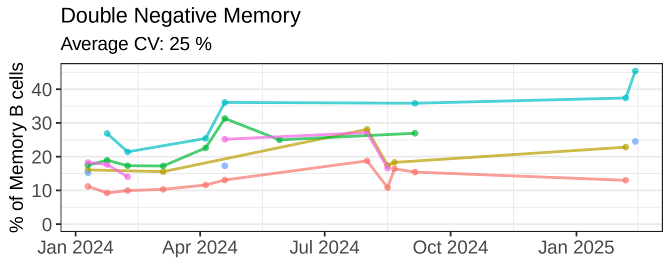

Double Negative Memory
Average CV: 25 %
% of Memory B cells
40
30
20
10
0
Jan 2024
Apr 2024
Jul 2024
Oct 2024
Jan 2025

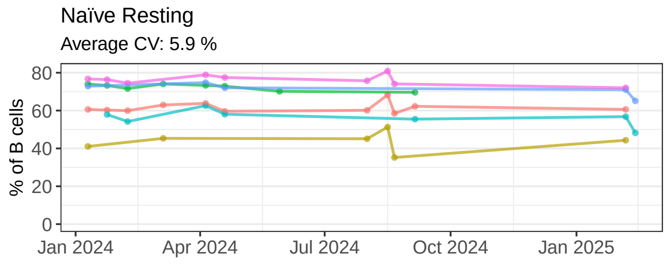

Naïve Resting
Average CV: 5.9 %
% of B cells
80
60
40
20
0
Jan 2024
Apr 2024
Jul 2024
Oct 2024
Jan 2025

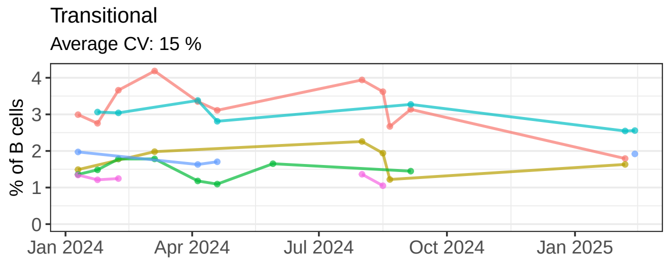
Transitional
Average CV: 15 %
% of B cells
0
1
2
3
4
Jan 2024
Apr 2024
Jul 2024
Oct 2024
Jan 2025

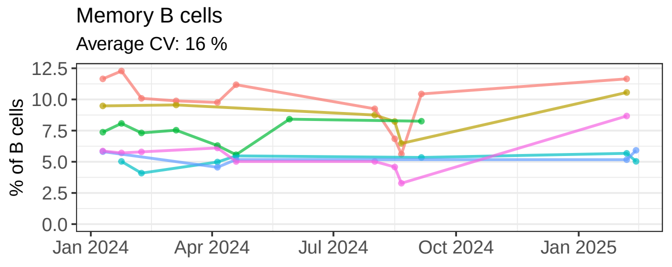
Memory B cells
Average CV: 16 %
% of B cells
12.5
10.0
7.5
5.0
2.5
0.0
Jan 2024
Apr 2024
Jul 2024
Oct 2024
Jan 2025

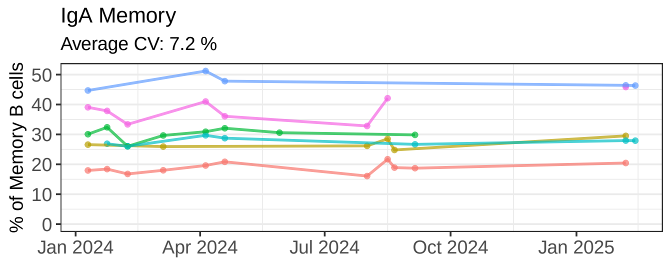
IgA Memory
Average CV: 7.2 %
% of Memory B cells
0
10
20
30
40
50
Jan 2024
Apr 2024
Jul 2024
Oct 2024
Jan 2025

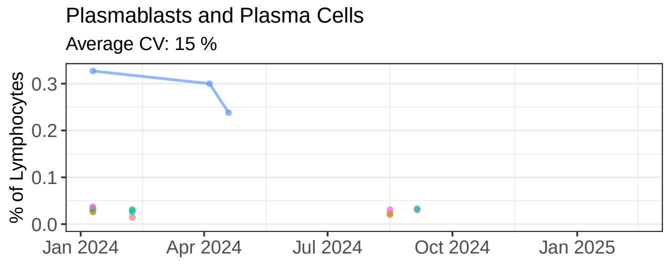

Plasmablasts and Plasma Cells
Average CV: 15 %
% of Lymphocytes
0.3
0.2
0.1
0.0
Jan 2024
Apr 2024
Jul 2024
Oct 2024
Jan 2025

B cells Plasmablasts and Plasma Cells

Average CV: 9 %

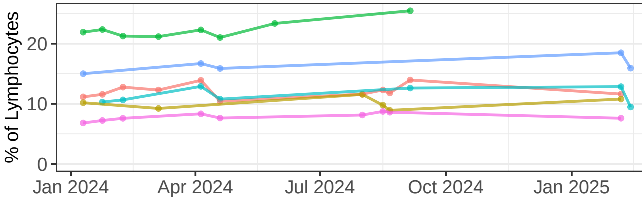

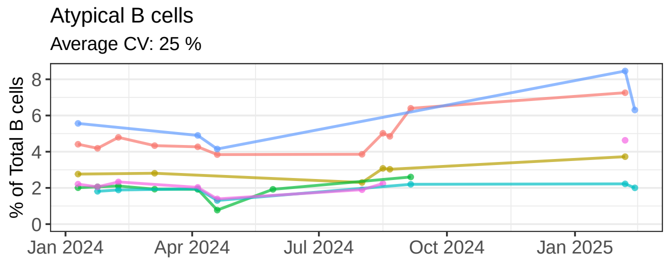

Atypical B cells
Average CV: 25 %
% of Total B cells
0
2
4
6
8
Jan 2024
Apr 2024
Jul 2024
Oct 2024
Jan 2025

CD8 CD38+ (activated)

Average CV: 29 %

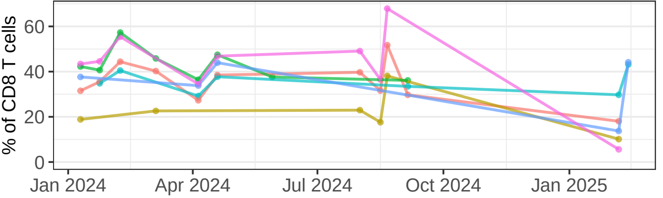

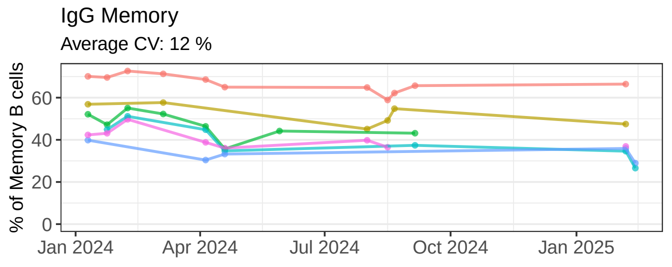
IgG Memory
Average CV: 12 %
% of Memory B cells
0
20
40
60
Jan 2024
Apr 2024
Jul 2024
Oct 2024
Jan 2025

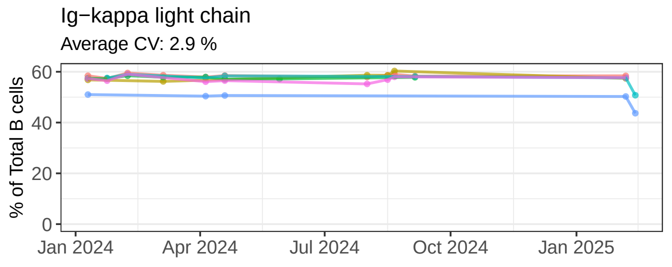

Ig−kappa light chain
Average CV: 2.9 %
% of Total B cells
0
20
40
60
Jan 2024
Apr 2024
Jul 2024
Oct 2024
Jan 2025

Ig−lambda light chain

Average CV: 3.2 %

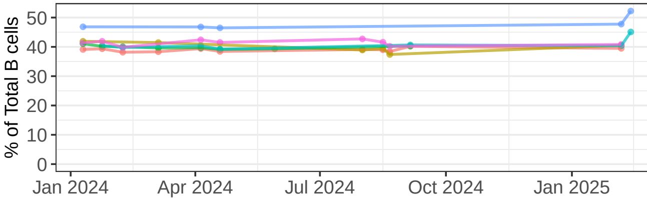

Supplement: Supplementary file 2 [file DataSheet2.pdf]
