# Supplementary material for: Advanced immunophenotyping of lymphocyte and monocyte subsets in healthy Australian adults using a novel spectral flow cytometry panel
Source: Front Immunol. 2025 Jul 22;16:1577206. doi: 10.3389/fimmu.2025.1577206 (PMC12322900; doi:10.3389/fimmu.2025.1577206)
Supplement: Supplementary file 3 [file DataSheet3.pdf]

Davies et al, Front. Immunol., doi:10.3389/fimmu.2025.1577206

Supplementary Material 3: Supplementary Figures 14-15

Supplementary Figure 14

A

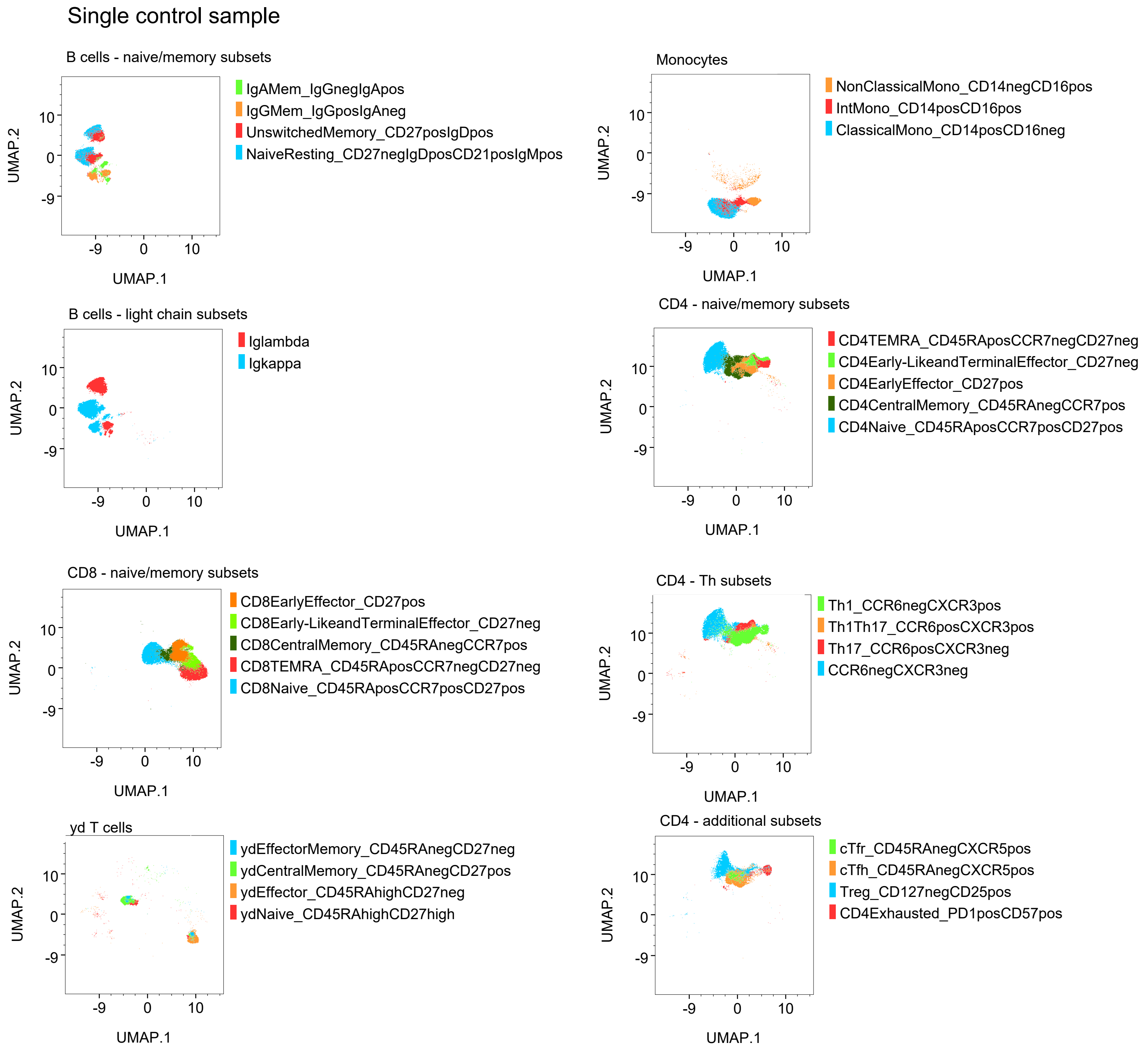

Supplementary Figure 14 - Manual gating overlay of UMAP dimensionality reduction. UMAP of Live CD45+ cells from one healthy control individual **(A)**. Manual gating was applied to the Live CD45+ cells, and these populations are overlayed in the labelled plots.

Supplementary Figure 15

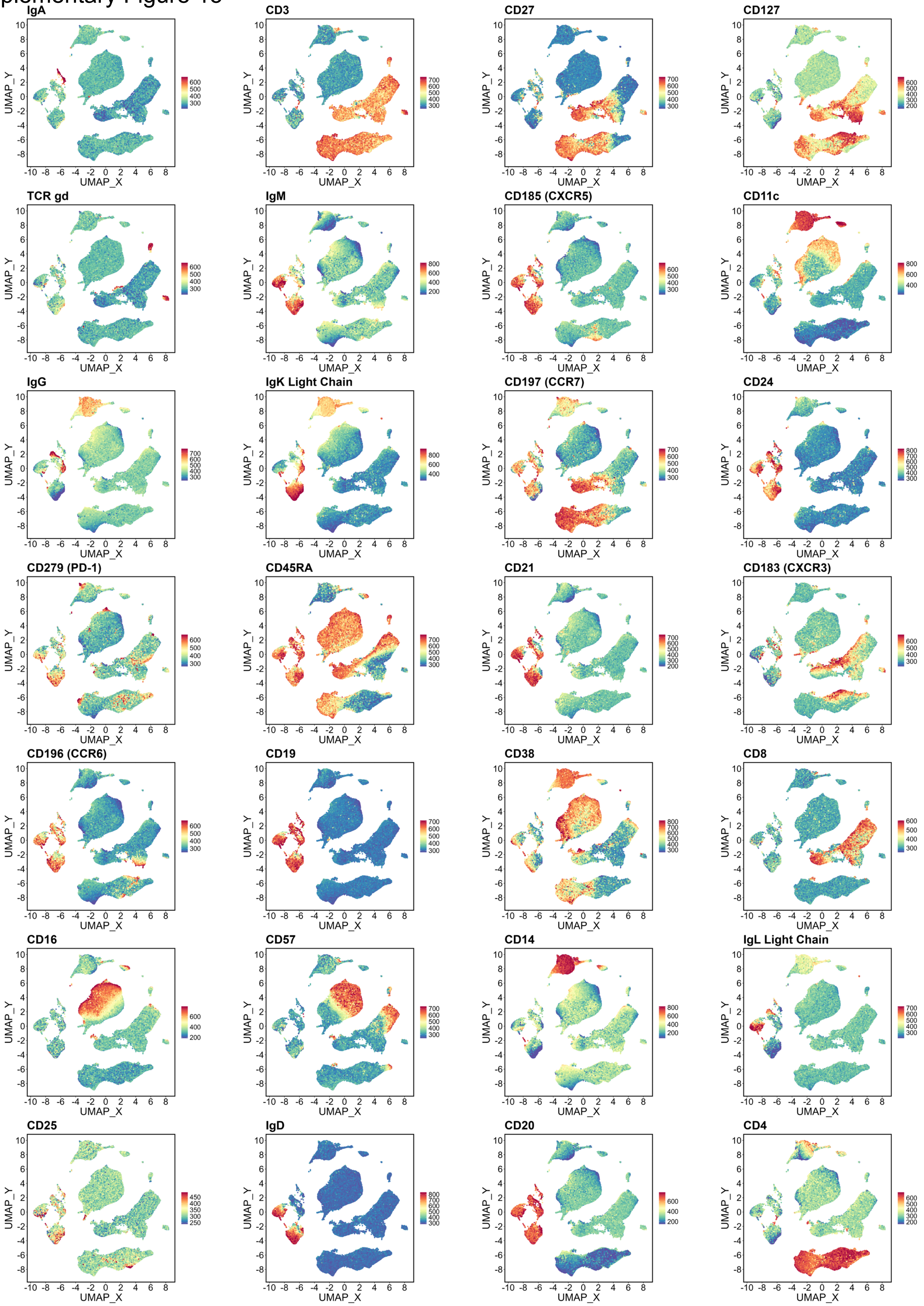

Supplementary Figure 15 - UMAP dimensionality reduction of nine batch repeats of one healthy control individual. Input cells were Live CD45+. Points are coloured by the scaled expression of the labelled marker.
