# Supplementary material for: Advanced immunophenotyping of lymphocyte and monocyte subsets in healthy Australian adults using a novel spectral flow cytometry panel
Source: Front Immunol. 2025 Jul 22;16:1577206. doi: 10.3389/fimmu.2025.1577206 (PMC12322900; doi:10.3389/fimmu.2025.1577206)
Supplement: Supplementary file 4 [file DataSheet4.pdf]

Davies et al, Front. Immunol., doi:10.3389/fimmu.2025.1577206

Supplementary Material 4: Supplementary Figure 16

Supplementary Figure 16 - Frequency of leukocyte populations. Healthy donors n=148 *. Grey boxes represent the median (middle line marker), 2.5th percentile (lower bound) and 97.5th percentile (upper bound), and the values of these percentiles are displayed on the right. Samples below the limit of detection (less than 100 events in the gate) are not shown on the plot. The number of samples below the limit of detection are written on the plot.

* Treg and cTfr healthy donors n = 138.

CD4 T cells

samples with too few events to calculate frequency: 0

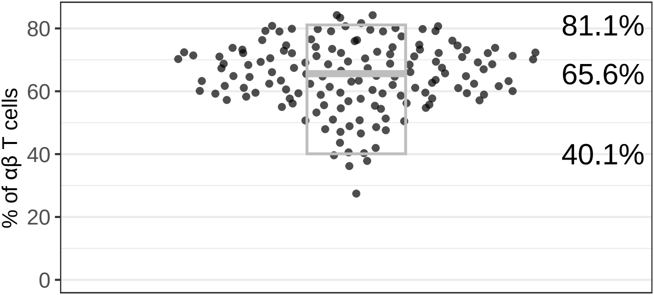

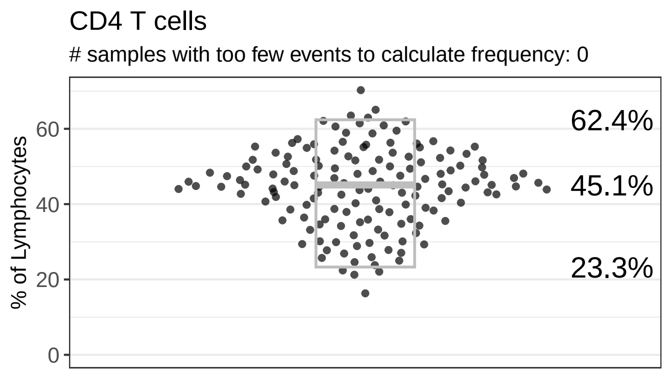
CD4 T cells
samples with too few events to calculate frequency: 0
% of Lymphocytes
0
20
40
60
62.4%
45.1%
23.3%

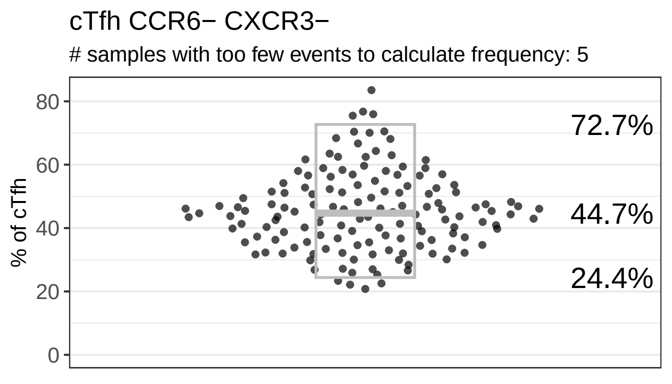
cTfh CCR6− CXCR3−
samples with too few events to calculate frequency: 5
% of cTfh
80
60
40
20
0
72.7%
44.7%
24.4%

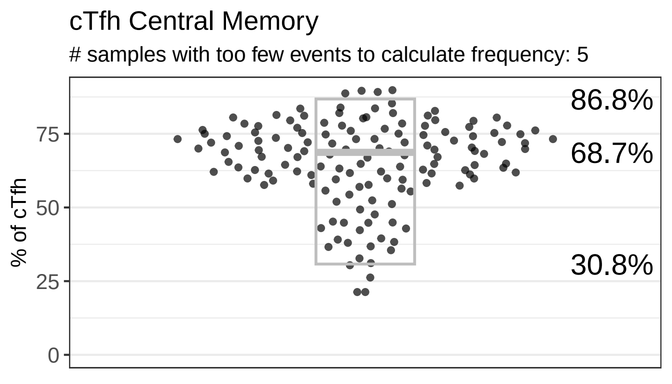
cTfh Central Memory
samples with too few events to calculate frequency: 5
% of cTfh
0
25
50
75
86.8%
68.7%
30.8%

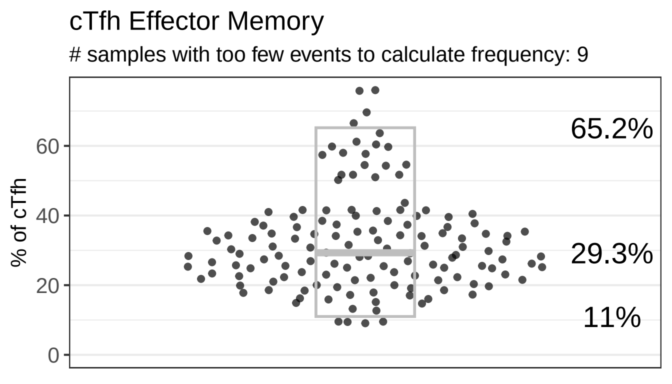
cTfh Effector Memory
samples with too few events to calculate frequency: 9
% of cTfh
0
20
40
60
65.2%
29.3%
11%

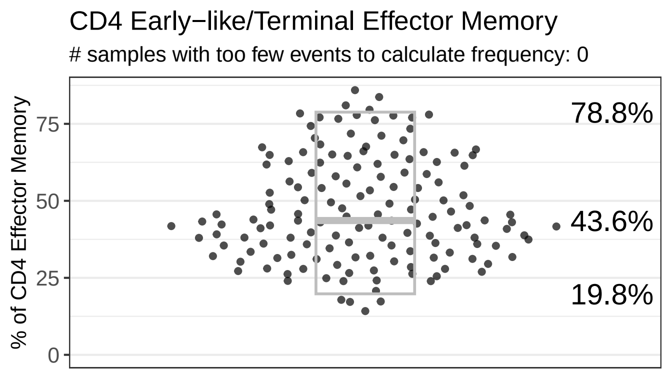
CD4 Early−like/Terminal Effector Memory
samples with too few events to calculate frequency: 0
% of CD4 Effector Memory
75
50
25
0
78.8%
43.6%
19.8%

Th1

samples with too few events to calculate frequency: 1

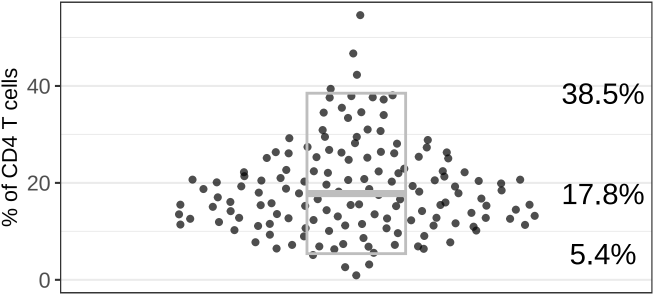

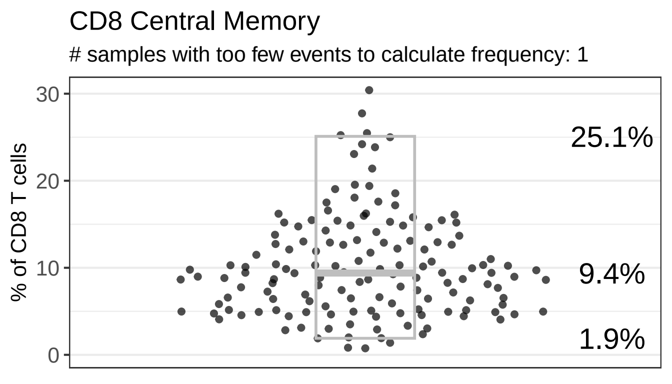
CD8 Central Memory
samples with too few events to calculate frequency: 1
% of CD8 T cells
30
20
10
0
25.1%
9.4%
1.9%

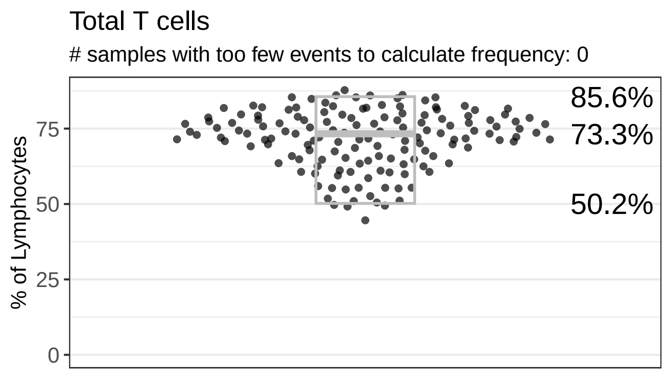
Total T cells
samples with too few events to calculate frequency: 0
% of Lymphocytes
0
25
50
75
85.6%
73.3%
50.2%

Th17

samples with too few events to calculate frequency: 1

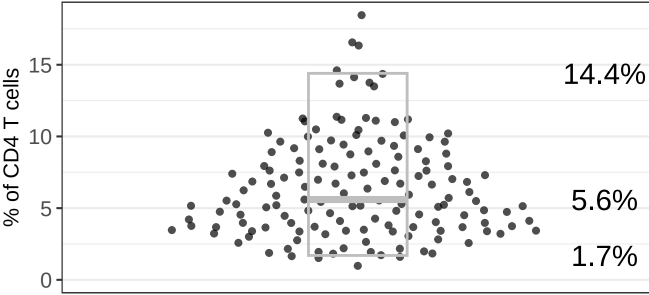

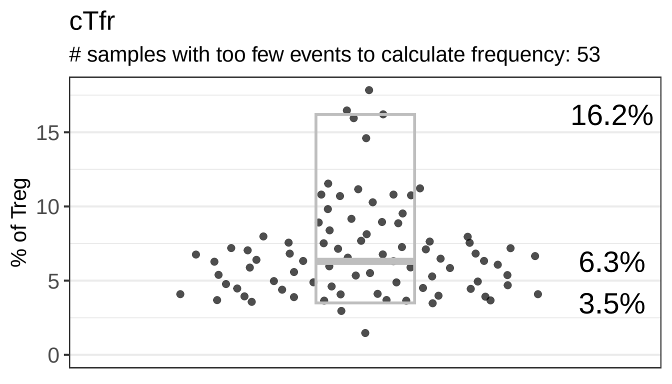
cTfr
samples with too few events to calculate frequency: 53
% of Treg
0
5
10
15
16.2%
6.3%
3.5%

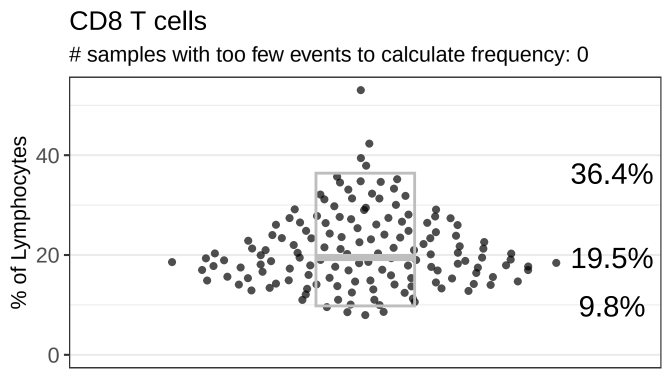
CD8 T cells
samples with too few events to calculate frequency: 0
% of Lymphocytes
40
20
0
36.4%
19.5%
9.8%

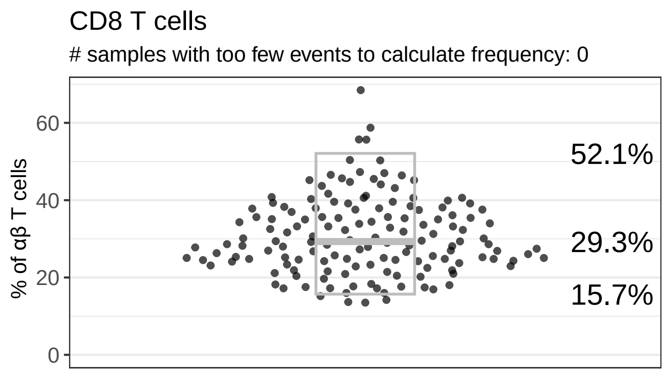
CD8 T cells
samples with too few events to calculate frequency: 0
% of αβ T cells
0
20
40
60
52.1%
29.3%
15.7%

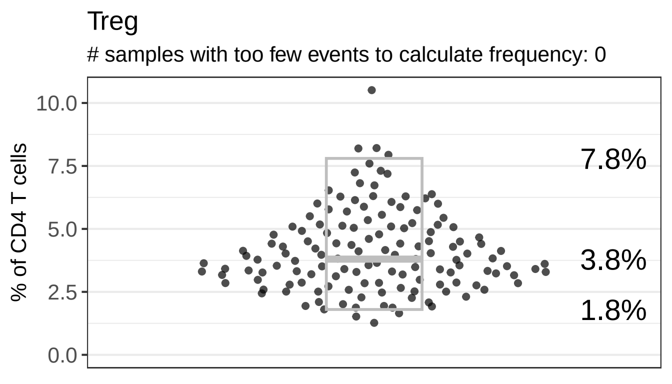
Treg
samples with too few events to calculate frequency: 0
10.0
7.5
5.0
2.5
0.0
% of CD4 T cells
7.8%
3.8%
1.8%

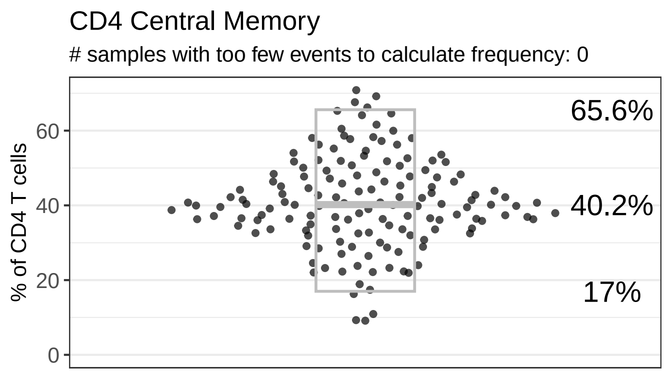
CD4 Central Memory
samples with too few events to calculate frequency: 0
% of CD4 T cells
0
20
40
60
65.6%
40.2%
17%

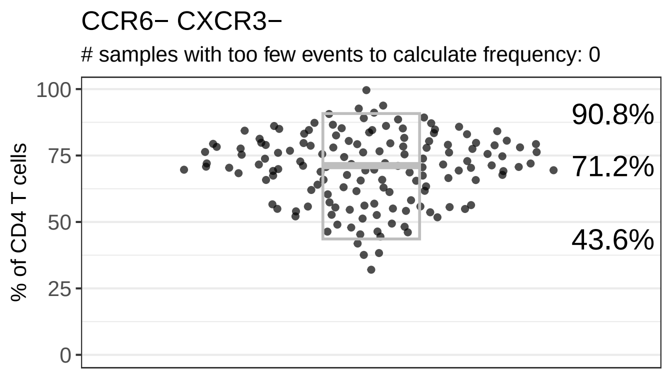
CCR6− CXCR3−
samples with too few events to calculate frequency: 0
% of CD4 T cells
100
75
50
25
0
90.8%
71.2%
43.6%

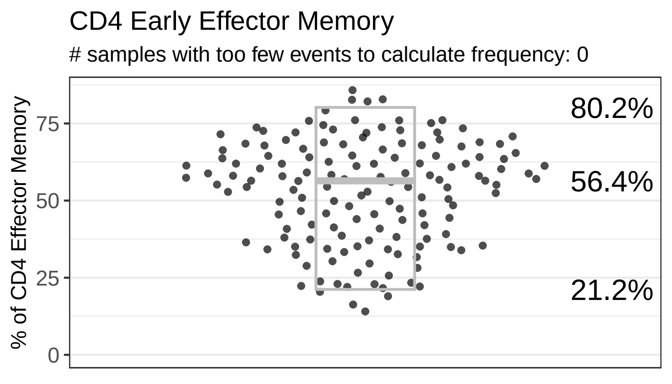
CD4 Early Effector Memory
samples with too few events to calculate frequency: 0
% of CD4 Effector Memory
0
25
50
75
80.2%
56.4%
21.2%

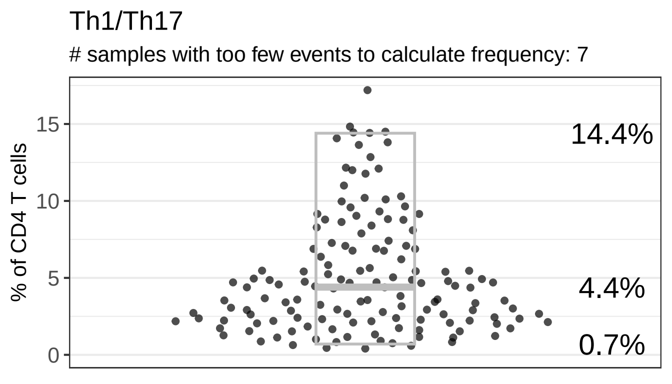
Th1/Th17
samples with too few events to calculate frequency: 7
% of CD4 T cells
15
10
5
0
14.4%
4.4%
0.7%

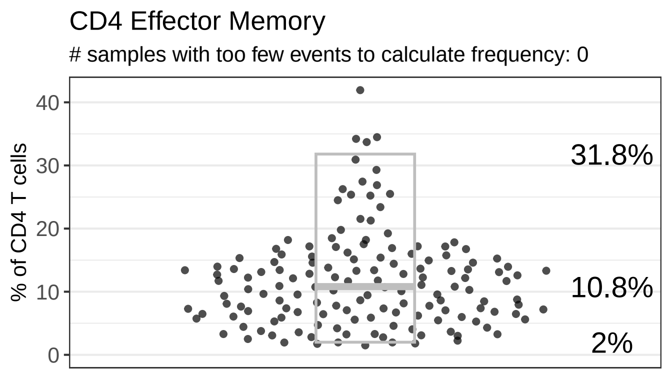
CD4 Effector Memory
samples with too few events to calculate frequency: 0
% of CD4 T cells
40
30
20
10
0
31.8%
10.8%
2%

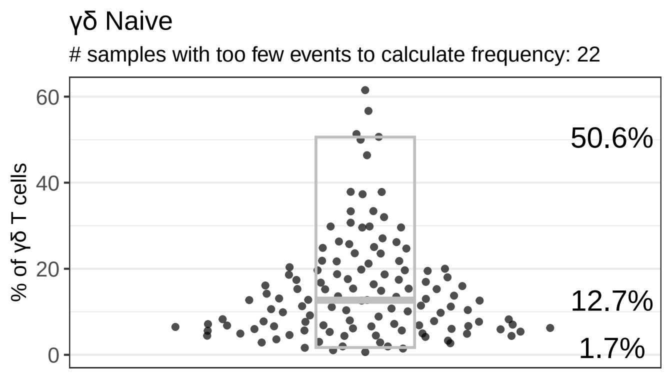
γδ Naive
samples with too few events to calculate frequency: 22
60
40
20
0
% of γδ T cells
50.6%
12.7%
1.7%

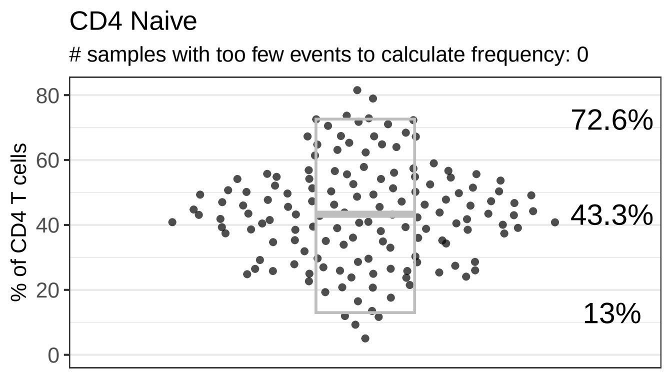

CD4 Naive
samples with too few events to calculate frequency: 0
% of CD4 T cells
0
20
40
60
80
72.6%
43.3%
13%

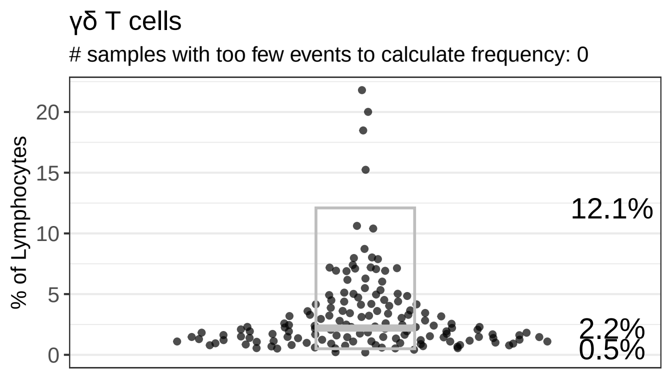
γδ T cells
samples with too few events to calculate frequency: 0
% of Lymphocytes
20
15
10
5
0
12.1%
2.2%
0.5%

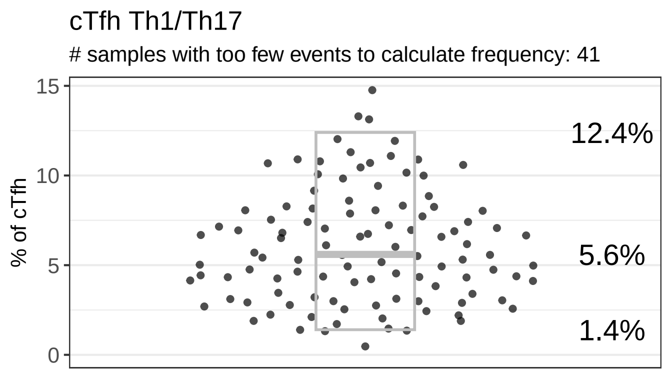
cTfh Th1/Th17
samples with too few events to calculate frequency: 41
% of cTfh
15
10
5
0
12.4%
5.6%
1.4%

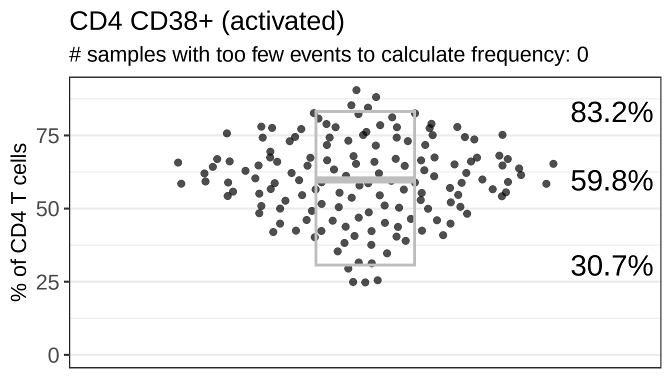
CD4 CD38+ (activated)
samples with too few events to calculate frequency: 0
% of CD4 T cells
0
25
50
75
83.2%
59.8%
30.7%

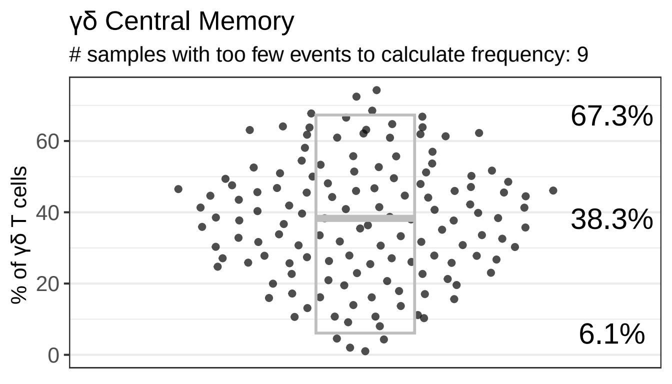
γδ Central Memory
samples with too few events to calculate frequency: 9
% of γδ T cells
0
20
40
60
67.3%
38.3%
6.1%

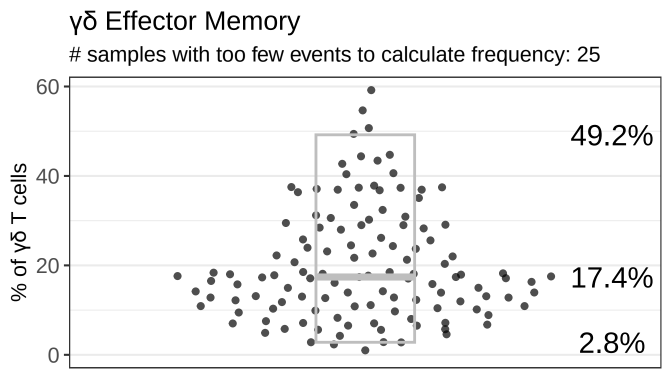
γδ Effector Memory
samples with too few events to calculate frequency: 25
% of γδ T cells
60
40
20
0
49.2%
17.4%
2.8%

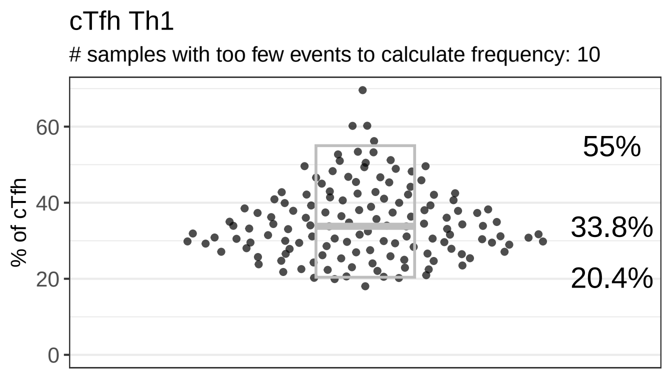
cTfh Th1
samples with too few events to calculate frequency: 10
% of cTfh
0
20
40
60
55%
33.8%
20.4%

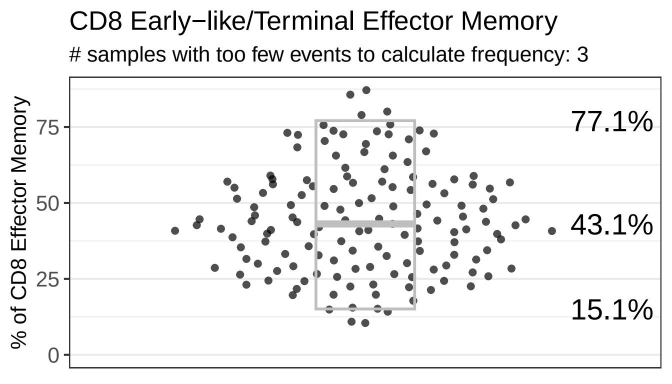
CD8 Early−like/Terminal Effector Memory
samples with too few events to calculate frequency: 3
% of CD8 Effector Memory
0
25
50
75
77.1%
43.1%
15.1%

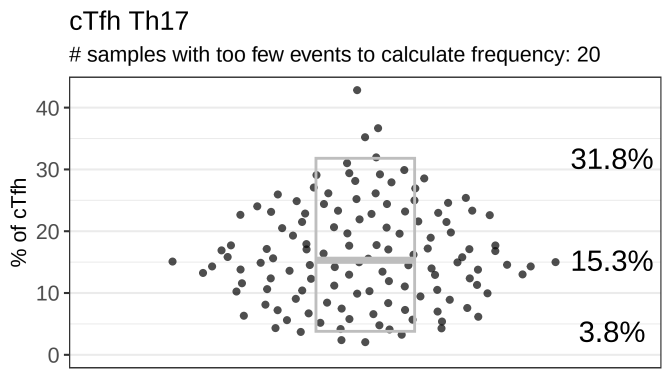
cTfh Th17
samples with too few events to calculate frequency: 20
40
30
20
10
0
% of cTfh
31.8%
15.3%
3.8%

CD4 Exhausted

samples with too few events to calculate frequency: 15

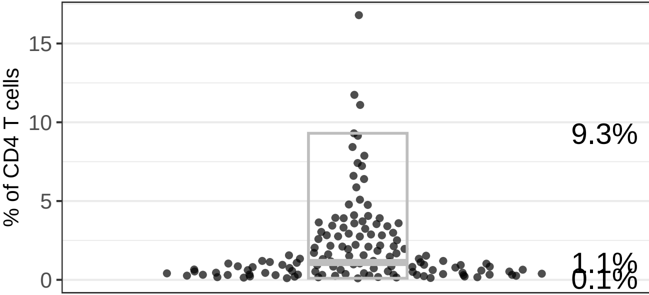

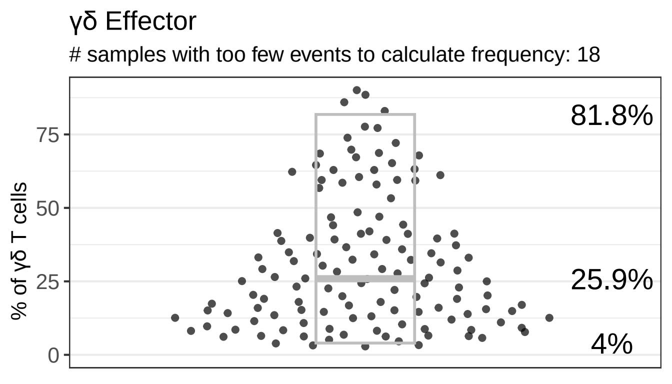
γδ Effector
samples with too few events to calculate frequency: 18
% of γδ T cells
0
25
50
75
81.8%
25.9%
4%

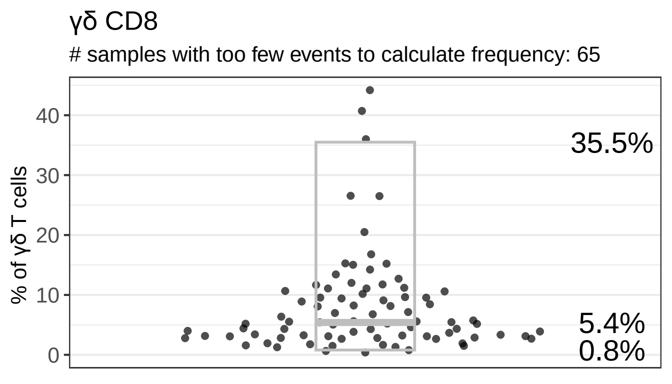
γδ CD8
samples with too few events to calculate frequency: 65
% of γδ T cells
0
10
20
30
40
35.5%
5.4%
0.8%

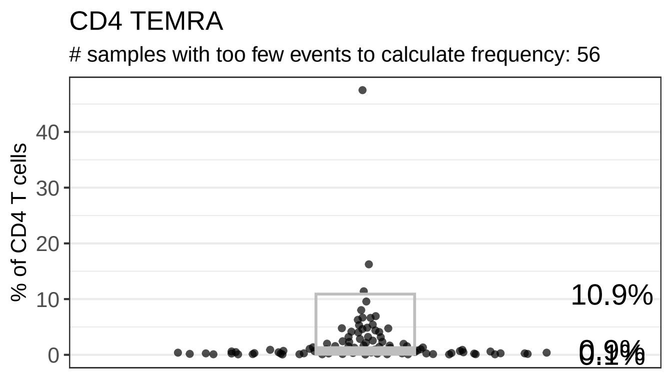
CD4 TEMRA
samples with too few events to calculate frequency: 56
% of CD4 T cells
40
30
20
10
0
10.9%

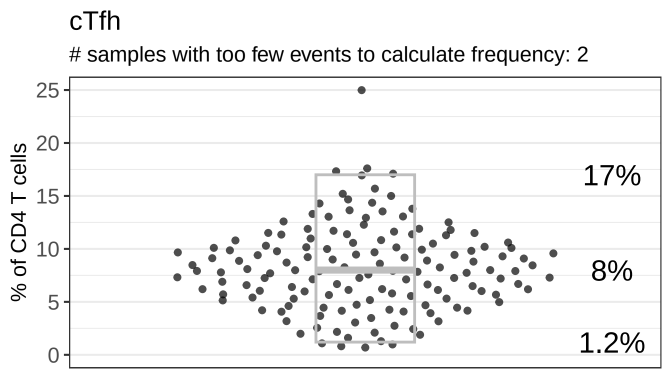
cTfh
samples with too few events to calculate frequency: 2
% of CD4 T cells
25
20
15
10
5
0
17%
8%
1.2%

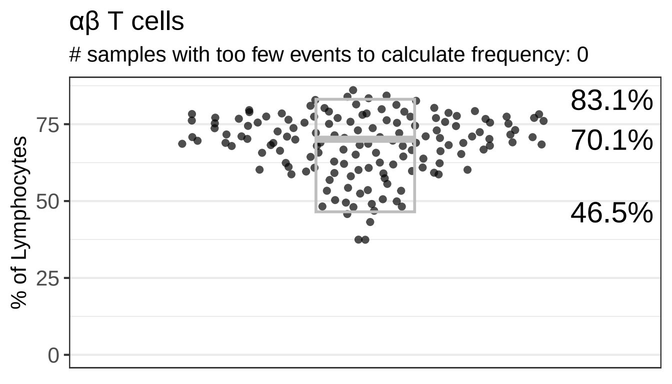
αβ T cells
samples with too few events to calculate frequency: 0
% of Lymphocytes
0
25
50
75
83.1%
70.1%
46.5%

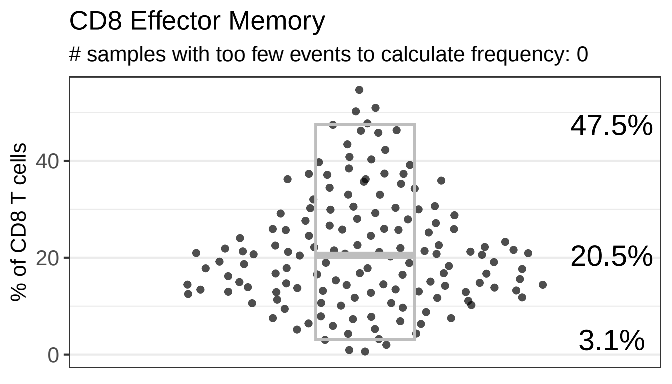
CD8 Effector Memory
samples with too few events to calculate frequency: 0
% of CD8 T cells
40
20
0
47.5%
20.5%
3.1%

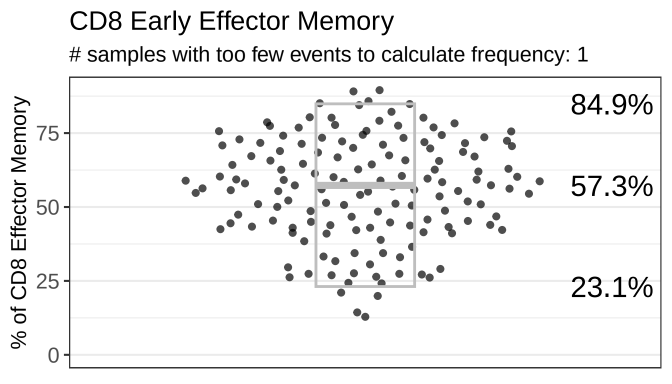

CD8 Early Effector Memory
samples with too few events to calculate frequency: 1
% of CD8 Effector Memory
0
25
50
75
84.9%
57.3%
23.1%

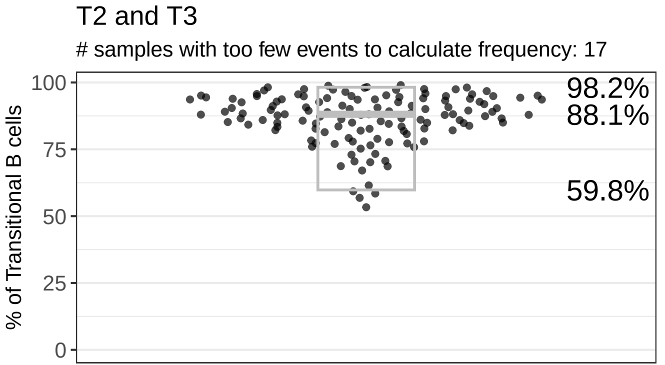
T2 and T3
samples with too few events to calculate frequency: 17
% of Transitional B cells
100
75
50
25
0
98.2%
88.1%
59.8%

Double Negative 2

samples with too few events to calculate frequency: 104

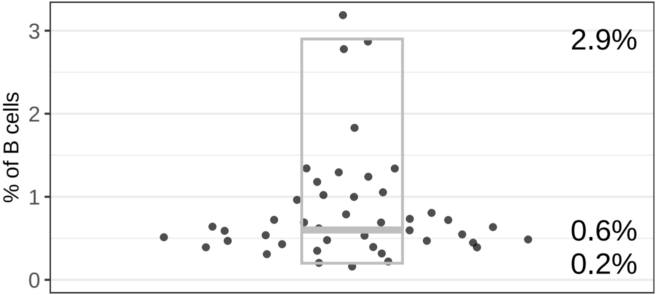

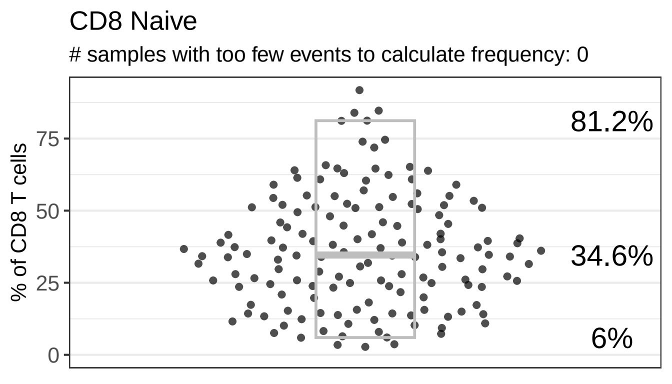
CD8 Naive
samples with too few events to calculate frequency: 0
% of CD8 T cells
75
50
25
0
81.2%
34.6%
6%

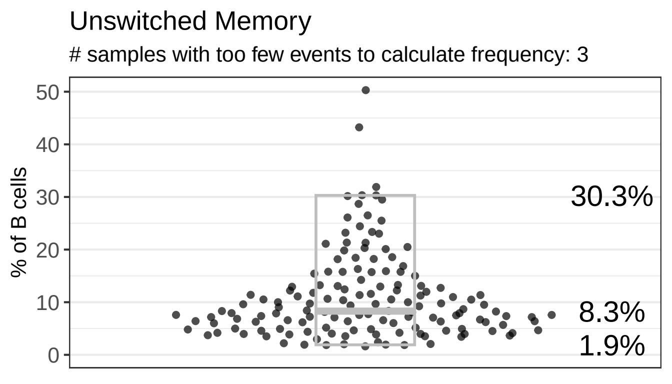
Unswitched Memory
samples with too few events to calculate frequency: 3
% of B cells
50
40
30
20
10
0
30.3%
8.3%
1.9%

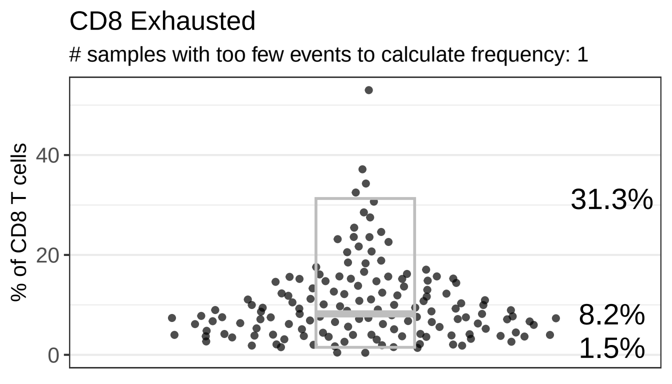
CD8 Exhausted
samples with too few events to calculate frequency: 1
% of CD8 T cells
0
20
40
31.3%
8.2%
1.5%

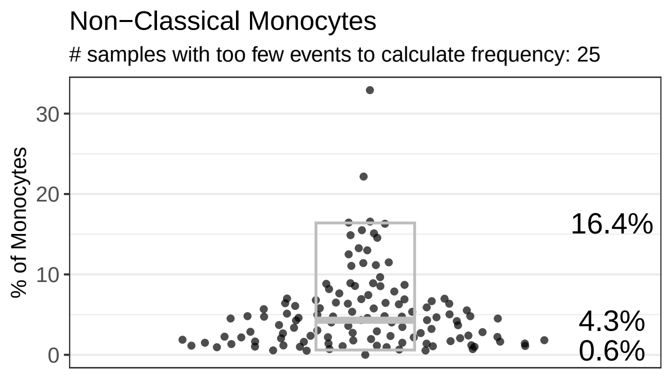

Non−Classical Monocytes
samples with too few events to calculate frequency: 25
% of Monocytes
30
20
10
0
16.4%
4.3%
0.6%

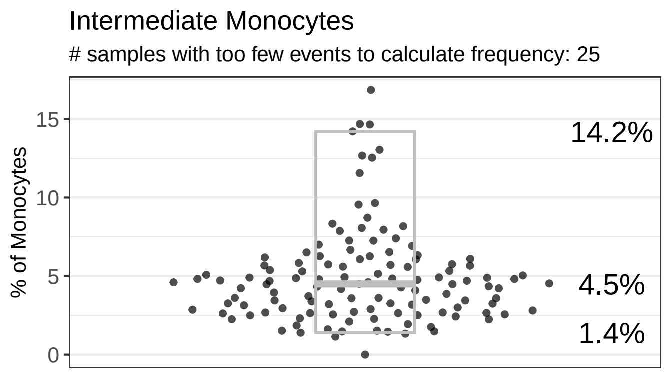
Intermediate Monocytes
samples with too few events to calculate frequency: 25
% of Monocytes
0
5
10
15
14.2%
4.5%
1.4%

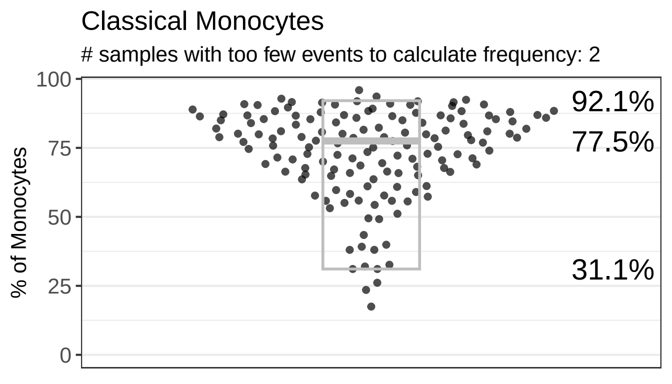
Classical Monocytes
samples with too few events to calculate frequency: 2
% of Monocytes
100
75
50
25
0
92.1%
77.5%
31.1%

Monocytes

samples with too few events to calculate frequency: 2

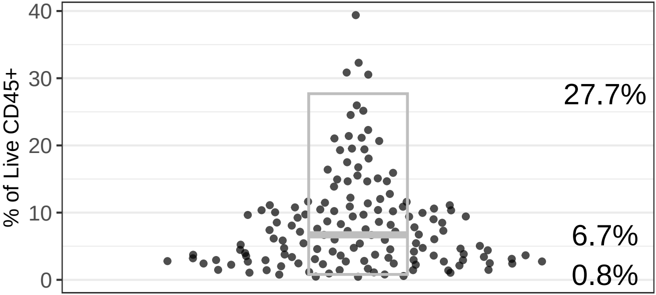

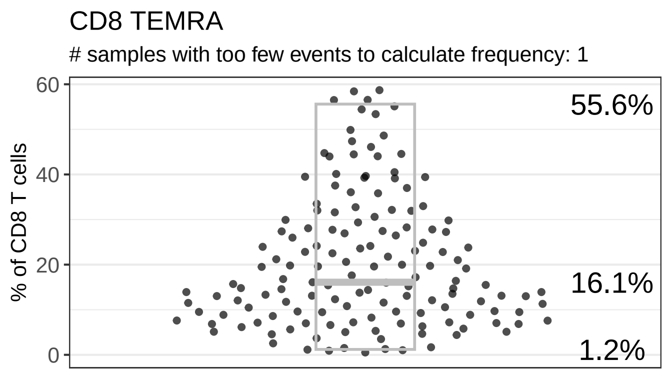
CD8 TEMRA
samples with too few events to calculate frequency: 1
% of CD8 T cells
60
40
20
0
55.6%
16.1%
1.2%

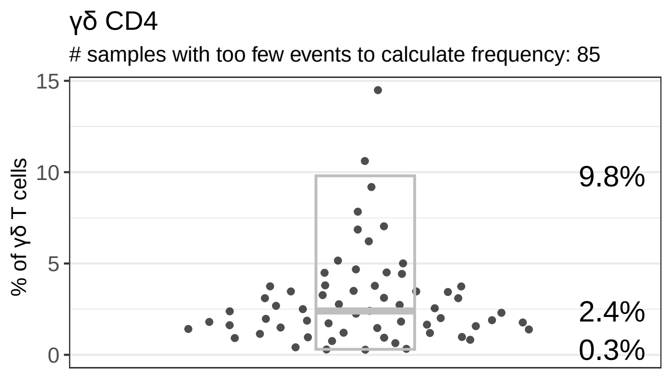
γδ CD4
samples with too few events to calculate frequency: 85
15
10
5
0
% of γδ T cells
9.8%
2.4%
0.3%

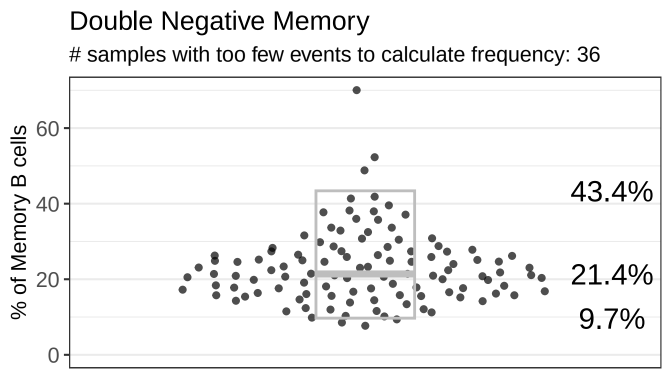
Double Negative Memory
samples with too few events to calculate frequency: 36
% of Memory B cells
0
20
40
60
43.4%
21.4%
9.7%

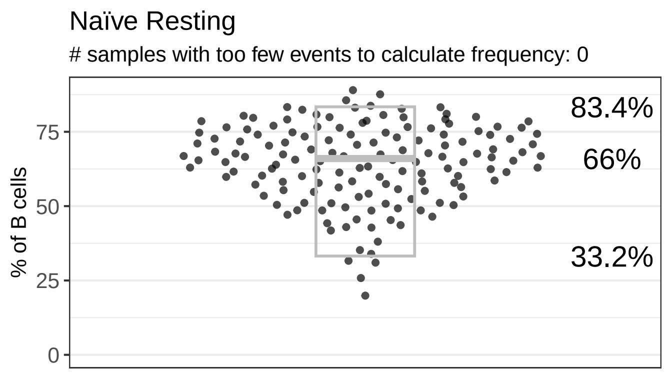
Naïve Resting
samples with too few events to calculate frequency: 0
% of B cells
75
50
25
0
83.4%
66%
33.2%

Transitional

samples with too few events to calculate frequency: 13

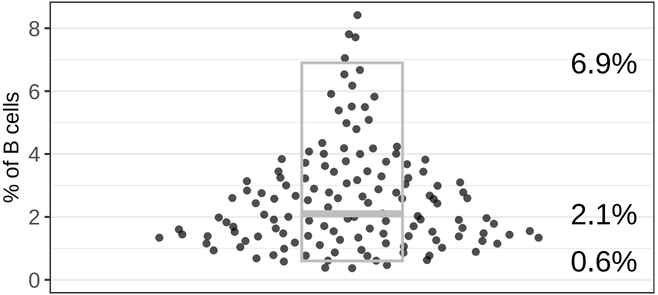

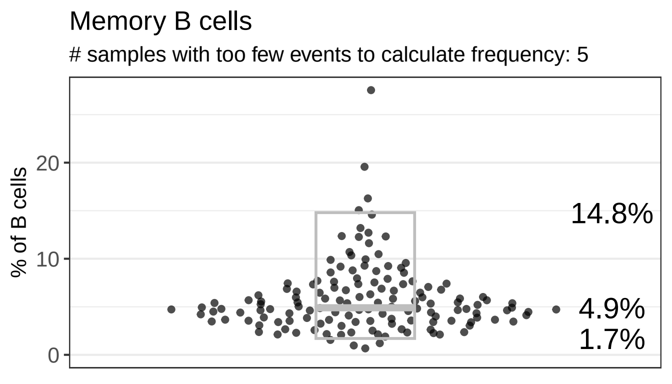
Memory B cells
samples with too few events to calculate frequency: 5
% of B cells
0
10
20
14.8%
4.9%
1.7%

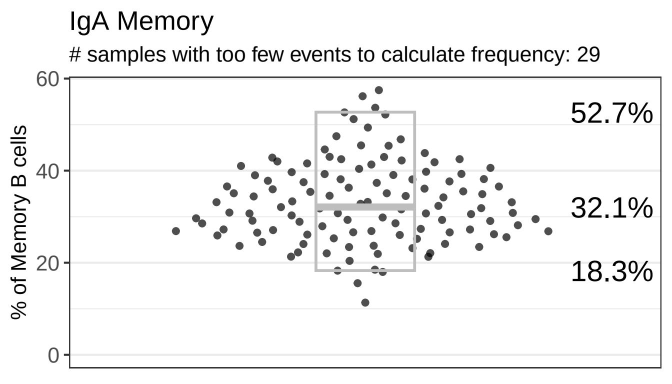

IgA Memory
samples with too few events to calculate frequency: 29
% of Memory B cells
60
40
20
0
52.7%
32.1%
18.3%

Plasmablasts and Plasma Cells

samples with too few events to calculate frequency: 108

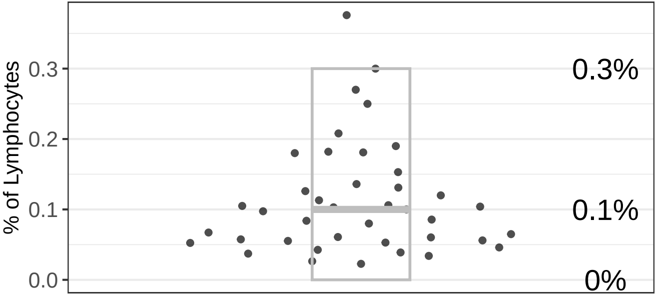

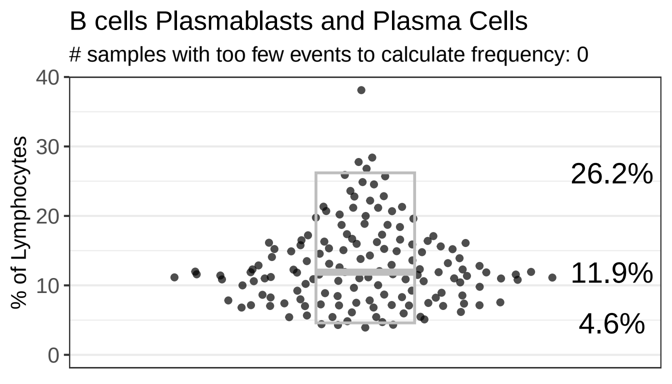
B cells Plasmablasts and Plasma Cells
samples with too few events to calculate frequency: 0
% of Lymphocytes
40
30
20
10
0
26.2%
11.9%
4.6%

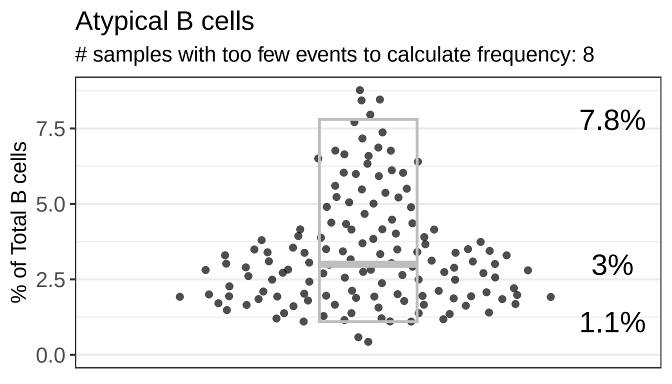
Atypical B cells
samples with too few events to calculate frequency: 8
% of Total B cells
7.5
5.0
2.5
0.0
7.8%
3%
1.1%

CD8 CD38+ (activated)

samples with too few events to calculate frequency: 0

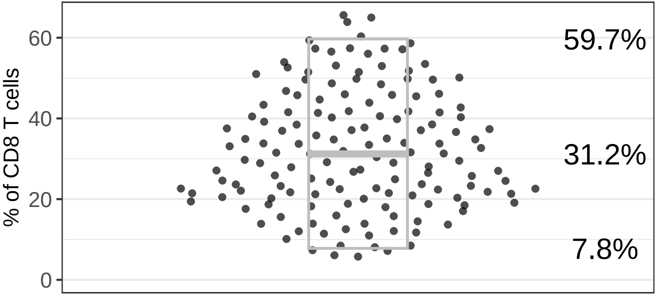

IgG Memory

samples with too few events to calculate frequency: 16

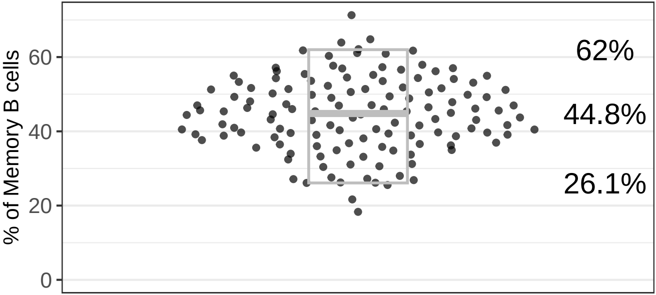

Ig−kappa light chain

samples with too few events to calculate frequency: 0

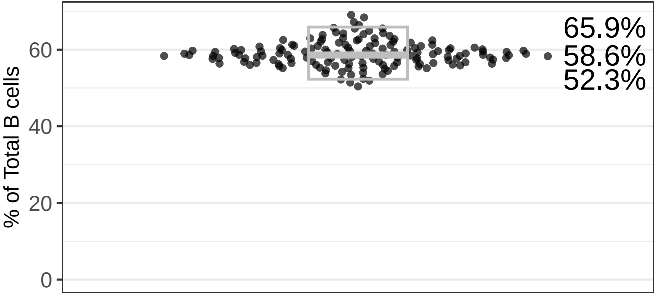

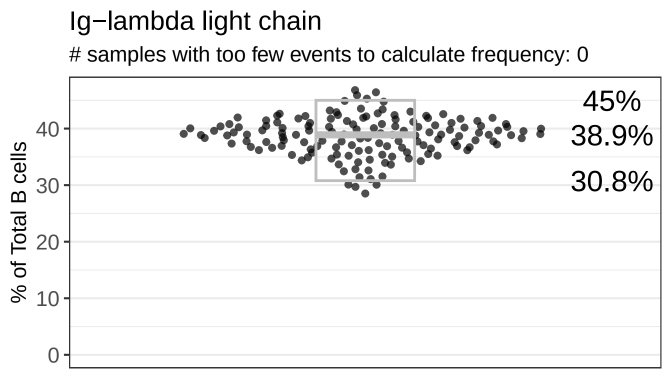

Ig−lambda light chain
samples with too few events to calculate frequency: 0
% of Total B cells
0
10
20
30
40
45%
38.9%
30.8%
